# Pathway-Driven Coordinated Telehealth System for Management of Patients With Single or Multiple Chronic Diseases in China: System Development and Retrospective Study

**DOI:** 10.2196/27228

**Published:** 2021-05-17

**Authors:** Zheyu Wang, Jiye An, Hui Lin, Jiaqiang Zhou, Fang Liu, Juan Chen, Huilong Duan, Ning Deng

**Affiliations:** 1 Ministry of Education Key Laboratory of Biomedical Engineering College of Biomedical Engineering and Instrument Science Zhejiang University Hangzhou China; 2 Sir Run-Run Shaw Hospital Zhejiang University School of Medicine Zhejiang University Hangzhou China; 3 General Hospital of Ningxia Medical University Yinchuan China

**Keywords:** chronic disease, telehealth system, integrated care, pathway, ontology

## Abstract

**Background:**

Integrated care enhanced with information technology has emerged as a means to transform health services to meet the long-term care needs of patients with chronic diseases. However, the feasibility of applying integrated care to the emerging “three-manager” mode in China remains to be explored. Moreover, few studies have attempted to integrate multiple types of chronic diseases into a single system.

**Objective:**

The aim of this study was to develop a coordinated telehealth system that addresses the existing challenges of the “three-manager” mode in China while supporting the management of single or multiple chronic diseases.

**Methods:**

The system was designed based on a tailored integrated care model. The model was constructed at the individual scale, mainly focusing on specifying the involved roles and responsibilities through a universal care pathway. A custom ontology was developed to represent the knowledge contained in the model. The system consists of a service engine for data storage and decision support, as well as different forms of clients for care providers and patients. Currently, the system supports management of three single chronic diseases (hypertension, type 2 diabetes mellitus, and chronic obstructive pulmonary disease) and one type of multiple chronic conditions (hypertension with type 2 diabetes mellitus). A retrospective study was performed based on the long-term observational data extracted from the database to evaluate system usability, treatment effect, and quality of care.

**Results:**

The retrospective analysis involved 6964 patients with chronic diseases and 249 care providers who have registered in our system since its deployment in 2015. A total of 519,598 self-monitoring records have been submitted by the patients. The engine could generate different types of records regularly based on the specific care pathway. Results of the comparison tests and causal inference showed that a part of patient outcomes improved after receiving management through the system, especially the systolic blood pressure of patients with hypertension (*P*<.001 in all comparison tests and an approximately 5 mmHg decrease after intervention via causal inference). A regional case study showed that the work efficiency of care providers differed among individuals.

**Conclusions:**

Our system has potential to provide effective management support for single or multiple chronic conditions simultaneously. The tailored closed-loop care pathway was feasible and effective under the “three-manager” mode in China. One direction for future work is to introduce advanced artificial intelligence techniques to construct a more personalized care pathway.

## Introduction

### Background

Chronic diseases are the most prevalent and costly health conditions worldwide [1]. Patient self-management combined with timely intervention from care providers are essential to control the progression of chronic diseases [2]. However, within traditional care settings, the disconnected and time-consuming management procedure is unable to meet the long-term care needs of patients [3,4]. Furthermore, several patients with chronic diseases live with more than one chronic condition (ie, multiple chronic conditions [MCC]), which create diverse, and sometimes contradictory, needs for health services [5,6].

Integrated care has been proposed as a means to meet the above challenges by transforming traditional health services [7]. In an integrated care setting, health services are provided by a coordinated multidisciplinary team of care providers. The core objective is to implement patient-centered health systems through comprehensive delivery of quality services across the life course [8]. Further, the advent of information technologies has promoted the delivery of integrated care services, which can be understood from different scales. From an individual scale, information technologies are crucial to facilitate the development of shared care plans [7], which clearly articulates the roles of care providers and patients in the care process to deliver more personalized and targeted care [9]. From a group scale, information technologies play a key role in achieving the goals of bidirectional communication within care provider teams and provision of continuous self-management support to patients [10].

As practical applications of information technologies in the health care domain, telehealth systems have demonstrated potential to improve the outcomes of chronic disease management [11-14]. Patient self-monitoring at home and remote guidance from care providers can be realized with the assistance of telehealth systems [15]. However, most of these systems focus on a single chronic disease, and few studies have attempted to integrate multiple types of chronic diseases into one system [16-19]. A telehealth system designed for managing multiple chronic illnesses simultaneously can not only support the management of patients with MCC but can also reduce the cost of developing multiple telehealth systems for managing different chronic diseases.

In China, the current health service system is in a three-tier form: community health service institutions at the bottom, secondary hospitals in the middle, and tertiary hospitals at the top [20]. General practitioners (GPs) are at the core of primary health care (ie, community level), providing basic treatment and long-term care for patients, especially in the management of chronic diseases [21]. In response to the government policy on promoting integrated care [22], specialists and case managers (CMs) are gradually involved in the management to form a coordinated multidisciplinary team called the “three-manager” mode [23]. The specialists are mainly from secondary or tertiary hospitals, providing more specialized and professional treatment [21]. CMs are mainly composed of nurses who work together with GPs to assist them in their daily work, similar to other countries [24,25].

The “three-manager” mode has been implemented in several provinces of China; however, there remain some practical challenges, which result in a significant gap between standards of care and medical practice [26-28]. First, current management guidelines [29-31] do not clearly specify the responsibility of each role in the “three-manager” mode. Second, the unbalanced allocation of medical resources in China leads to a difference in the abilities of primary care providers [32-34]. GPs and CMs in remote areas may rarely perform comprehensive and effective management following the guidelines.

### Objectives

In this paper, we present the design, development, and retrospective evaluation of a telehealth system that simultaneously supports the management of single or multiple chronic diseases. The proposed system aims to address the existing challenges of the “three-manager” mode in China through a tailored integrated care model, mainly focusing on specifying the responsibility of involved roles and providing a universal care pathway for common chronic diseases. Currently, the system supports three main chronic conditions in China [35]: hypertension (HTN), type 2 diabetes mellitus (T2DM), and chronic obstructive pulmonary disease (COPD).

## Methods

### System Overview

**Figure 1:**
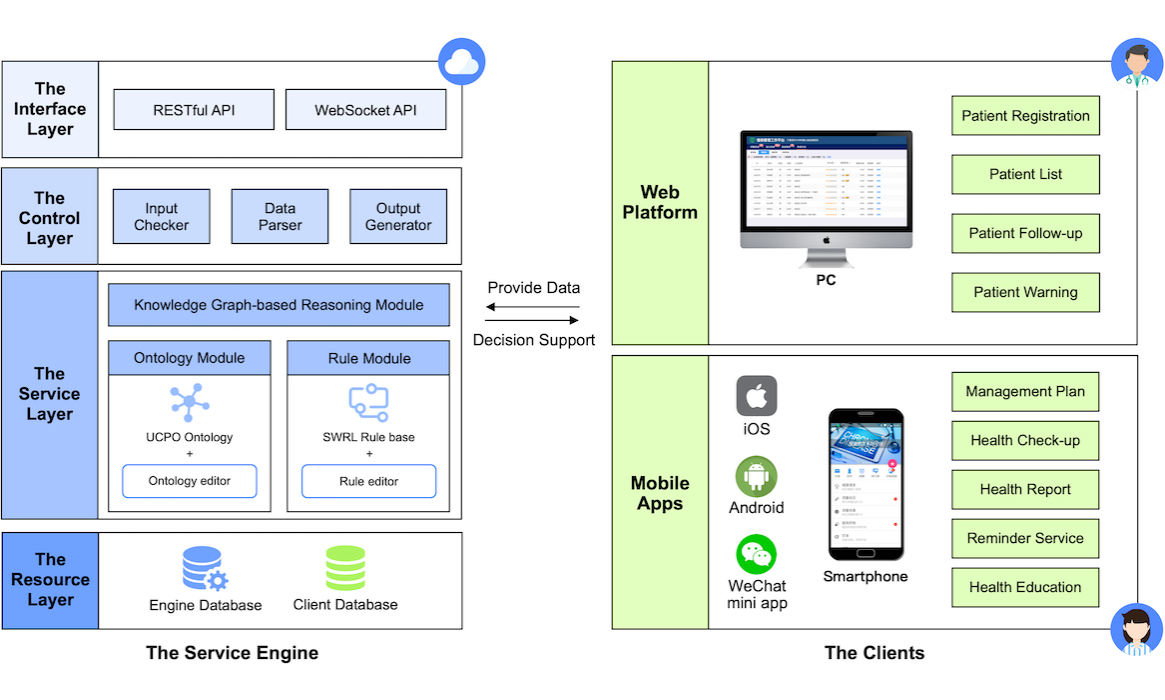
Architecture of the system. API: application programming interface; SWRL: Semantic Web Rule Language; UCPO: Universal Care Pathway Ontology.

Figure 1 illustrates the system architecture that consists of two components: (1) a service engine for data storage and decision support, and (2) clients for care providers and patients. Concretely, the service engine is a web service deployed on the cloud server, interacting with clients via several types of application programming interfaces (APIs). The core of the engine is a custom ontology called Universal Care Pathway Ontology (UCPO), which represents the knowledge contained in our pathway-driven integrated care model. Given specific patient data, the engine will generate a small-scale knowledge graph based on UCPO to provide personalized decision support.

The clients are represented in different forms for both care providers and patients. For care providers, the client is in the form of a website that can be accessed on their computers in the hospital or health center. Care providers can utilize the client to monitor patients and perform the intervention. For patients, the client is in the form of a mobile app that can be downloaded to their personal smartphones. Patients can use the client to check their self-management plans, perform self-monitoring, and receive health education. We provided three versions of our app: native apps, including Android and iOS versions, for patients who prefer a better user experience, and a WeChat mini program for patients who are more familiar with WeChat.

### Service Engine for Decision Support

#### Pathway Construction

**Figure 2:**
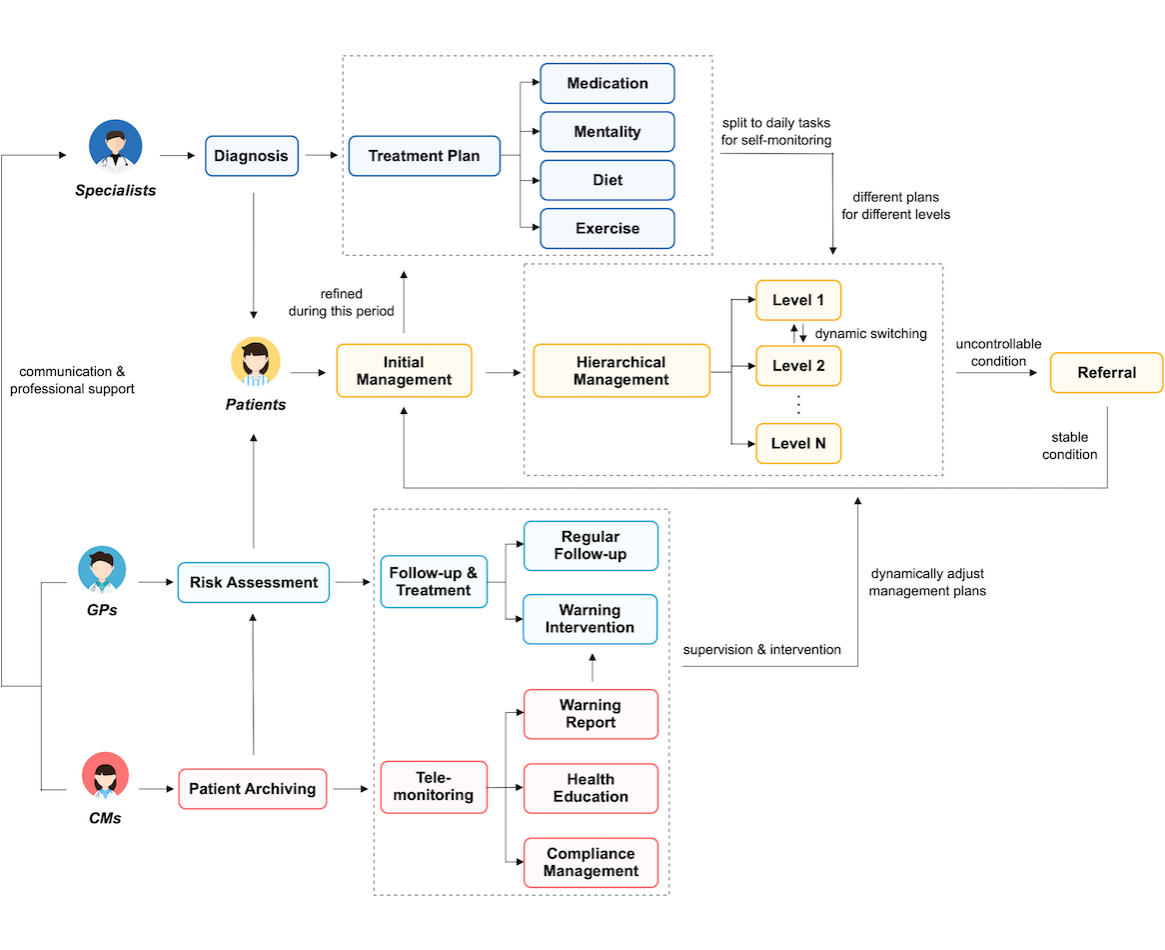
Pathway-driven integrated care model for the “three-manager” mode in China. CM: case manager; GP: general practitioner.

The implementation of the service engine was divided into three steps. First, we constructed a pathway-driven integrated care model for the management of common chronic diseases catering to the “three-manager” mode in China. Figure 2 demonstrates the diagram of the model. The tailored model was constructed at an individual scale, mainly concentrating on two aspects: the roles involved in the management process and their responsibilities. According to the “three-manager” mode, four roles participate in the management: specialists, GPs, CMs, and patients. The responsibility of each role was specified through a well-designed universal care pathway. To identify common parts in the management procedures of different diseases, we carried out a qualitative analysis on the management guidelines of the three most prevalent chronic conditions in China: HTN [29], T2DM [30], and COPD [31]. A total of 9 common tasks were defined in the pathway for long-term out-of-hospital management. Furthermore, we held several rounds of discussion with experienced physicians to specify the detailed contents of each task for specific diseases that are not mentioned in the guidelines. Table 1 summarizes the general definition of each task and their specific contents (adopted in our system) for the above three diseases. The detailed description of each disease-specific care pathway can be found in Multimedia Appendix 1.

A practical guideline for effective implementation based on the tailored integrated model can be described as a two-stage process: stage 1 involves the generation of a management plan and stage 2 involves the realization of long-term effective management. In stage 1, a patient should first be diagnosed with a specific disease (or multiple diseases) by specialists in secondary or tertiary hospitals. An initial treatment plan will be formulated for the patient, mainly focusing on drug therapy. If the patient’s clinical situation is stable with no indication for hospitalization, they will be sent to the affiliated primary care clinic or health center. GPs and CMs work collaboratively to perform the out-of-hospital management. The patient needs to register in the corresponding institution (ie, patient archiving in Figure 2) and undergo a risk assessment for the diagnosed disease before starting routine management. GPs should evaluate the associated risk factors of the patient to refine the treatment plan. Subsequently, the patient will enter the initial management period, during which CMs should help the patient to become familiar with self-management tools (eg, the smartphone app). Based on the self-monitoring records during this period as well as the risk assessment results, the patient will be classified into a specific level with a specific intensity of intervention (follow-up). The management level will be dynamically adjusted throughout the management process according to the up-to-date health status of the patient. Given the personalized management plan consisting of a treatment plan and follow-up plan, the patient will enter the formal management period (stage 2).

In stage 2, the patient needs to follow self-management regimens using the provided tools. GPs need to perform follow-ups regularly to obtain a detailed understanding of the patient’s situation (combined with their self-monitoring records) and adjust the treatment plan if necessary. The follow-up schedule should be adjusted according to the management level. CMs need to supervise the patient’s self-monitoring records through a telehealth terminal (eg, the web platform for care providers in our system). Once an unexpected condition occurs, such as low compliance or abnormal self-monitoring data, CMs should respond in a timely manner to the warning, including contacting the patient and reporting the condition to GPs. If the condition is out of control, GPs should suggest a referral for the patient to receive further treatment from specialists. Moreover, CMs also need to provide health education to improve patient awareness and self-management abilities.

**Table 1:** Common tasks extracted for out-of-hospital chronic disease management.

Common tasks	General definition	Hypertension	Type 2 diabetes	COPD^a^
Diagnosis	Diagnosis based on specific indicators of specific diseases	Based on clinical BP^b^ measurements	Based on clinical BG^c^ measurements	Based on pulmonary function test
Risk assessment	Evaluate specific risk factors of specific diseases to refine the treatment plan	Evaluate cardiovascular risk factors, target organ damage, and comorbidity	Evaluate BP, BG, and blood lipid levels	Evaluate PEF^d^ level and scales about COPD-specific health status (such as the CAT^e^)
Hierarchical management	Divide patients into different levels to effectively utilize existing resources	Divide into 2 levels according to whether the patients reach target BP	Divide into 3 levels according to whether the patients reach target BG	Divide into 4 levels based on the risk assessment results
Regular follow-up	Communicate with patients regularly to perform intervention	Once every 3 months for level I and once every 2-4 weeks for level II	Once every 3 months for level I, once every month for level II, and once every 2 weeks for level III	Once every 2 weeks for each level of patients
Abnormal condition intervention	Emergency treatment for abnormal self-monitoring data	Evaluate single BP values and weekly BP values	Evaluate single BG values and blood ketone levels	Evaluate single PEF values, scale results, and acute exacerbations
Medication guidance	Drug therapy for the specific disease	Select appropriate antihypertensive drugs for patients	Select appropriate hypoglycemic drugs or insulin injection	Select appropriate drugs such as SABA^f^, LAMA^g^, and LABA^h^
Lifestyle guidance	Nondrug therapy for the specific disease, generally include diet, exercise, and mentality	Reduce sodium intake, control body weight, avoid smoking and drinking, increase exercise, and reduce mental stress	Control body weight, balanced diet, reduce sodium intake, avoid smoking and drinking, moderate exercise, and reduce mental stress	Avoid smoking, increase regular exercise, and perform professional rehabilitation exercises
Health education	Provide knowledge of chronic diseases to increase patients’ awareness and self-management ability	Provide basic knowledge about hypertension to patients	Provide basic knowledge about diabetes to patients	Provide basic knowledge about COPD to patients
Compliance management	Enhance the motivation of patients with low self-management compliance	Perform extra follow-ups for patients with low self-management compliance	Perform extra follow-ups for patients with low self-management compliance	Perform extra follow-ups for patients with low self-management compliance

^a^COPD: chronic obstructive pulmonary disease.

^b^BP: blood pressure.

^c^BG: blood glucose.

^d^PEF: peak expiratory flow.

^e^CAT: COPD Assessment Test.

^f^SABA: short-acting beta-agonists.

^g^LAMA: long-acting muscarinic antagonists.

^h^LABA: long-acting beta-agonists.

#### Ontology-Based Model Representation

To incorporate the proposed model into our system, we utilized an ontological approach to implement pathway-driven decision support. A custom ontology called UCPO was constructed to represent the knowledge in our model, including structural information (ie, relationships among model elements) and medical knowledge (ie, task contents for a specific care pathway). Structural information is represented through a class hierarchy and based on the properties of ontology, whereas medical knowledge is represented through an external rule set compatible with ontology.

The construction of UCPO was divided into two phases. In the first phase, we represented structural information of the model following a widely used ontology engineering methodology [36]. In short, we first specified the domain and scope of UCPO using competency questions [37], and then defined the classes and class hierarchy of UCPO through a top-down approach based on existing ontologies and all terminologies contained in the model. Subsequently, we defined the properties of classes (including object properties and data properties) as well as property restrictions to describe the internal structure and precise semantics of concepts.

**Figure 3:**
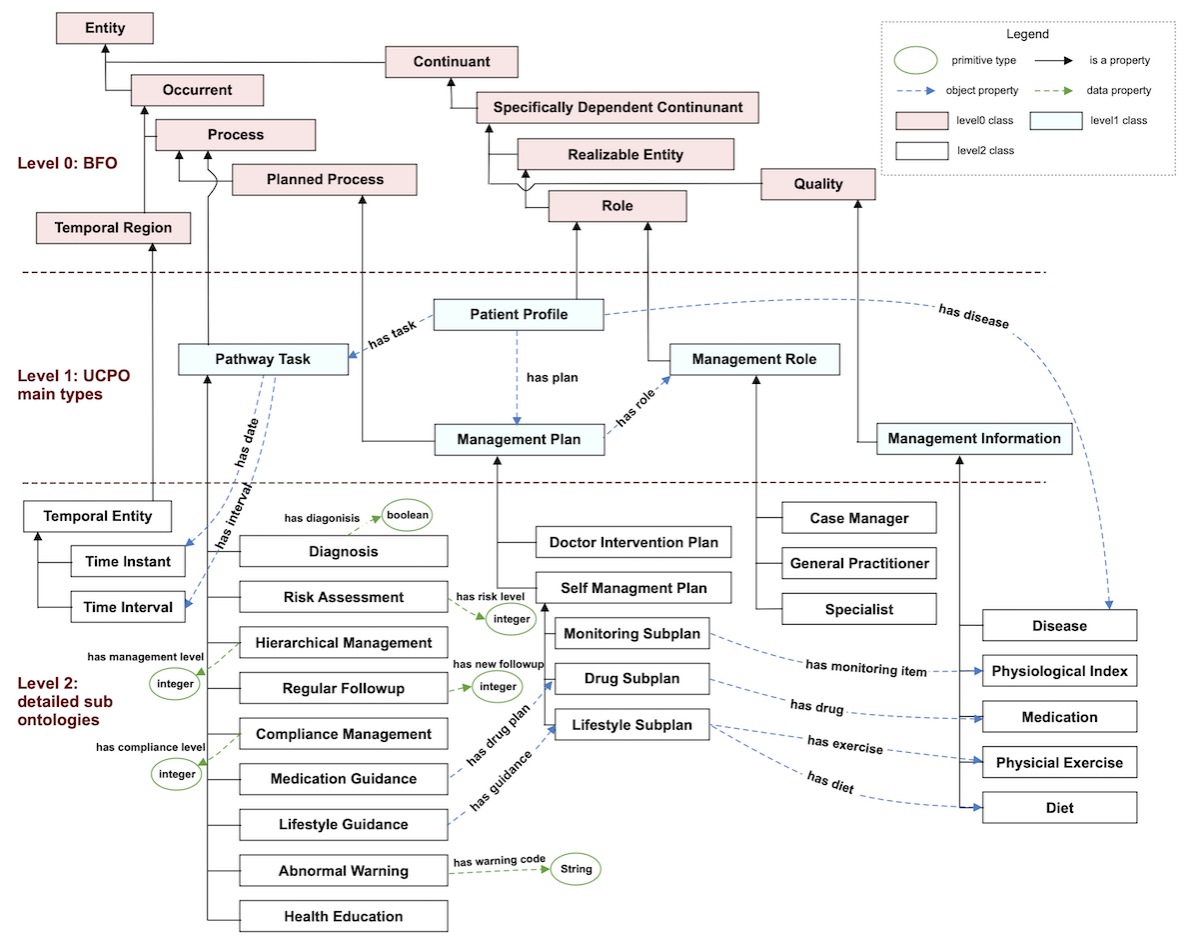
Class diagram of the main core of Universal Care Pathway Ontology (UCPO). BFO: basic formal ontology.

Figure 3 shows the class diagram and properties of the main UCPO core. UCPO was built in three levels of abstraction, inspired by a realistic ontology for diabetes treatment called DMTO [38]. Level 0 included several top-level universals from the most applicable top-level ontology (ie, basic formal ontology [39]). For all UCPO terms, subclasses of these universals were included to improve the interoperability for future extension and integration. Level 1 includes 5 terms that describe the core concepts in our model: pathway task, management plan, management role, patient profile, and management information. Level 2 includes the detailed elements for each Level 1 class. Classes are connected via various object properties. Several existing relevant ontologies were reused in UCPO, such as Ontology for General Medical Science [40] (for defining disease-related processes) and Ontology of Adverse Events [41] (for defining adverse events).

**Figure 4:**
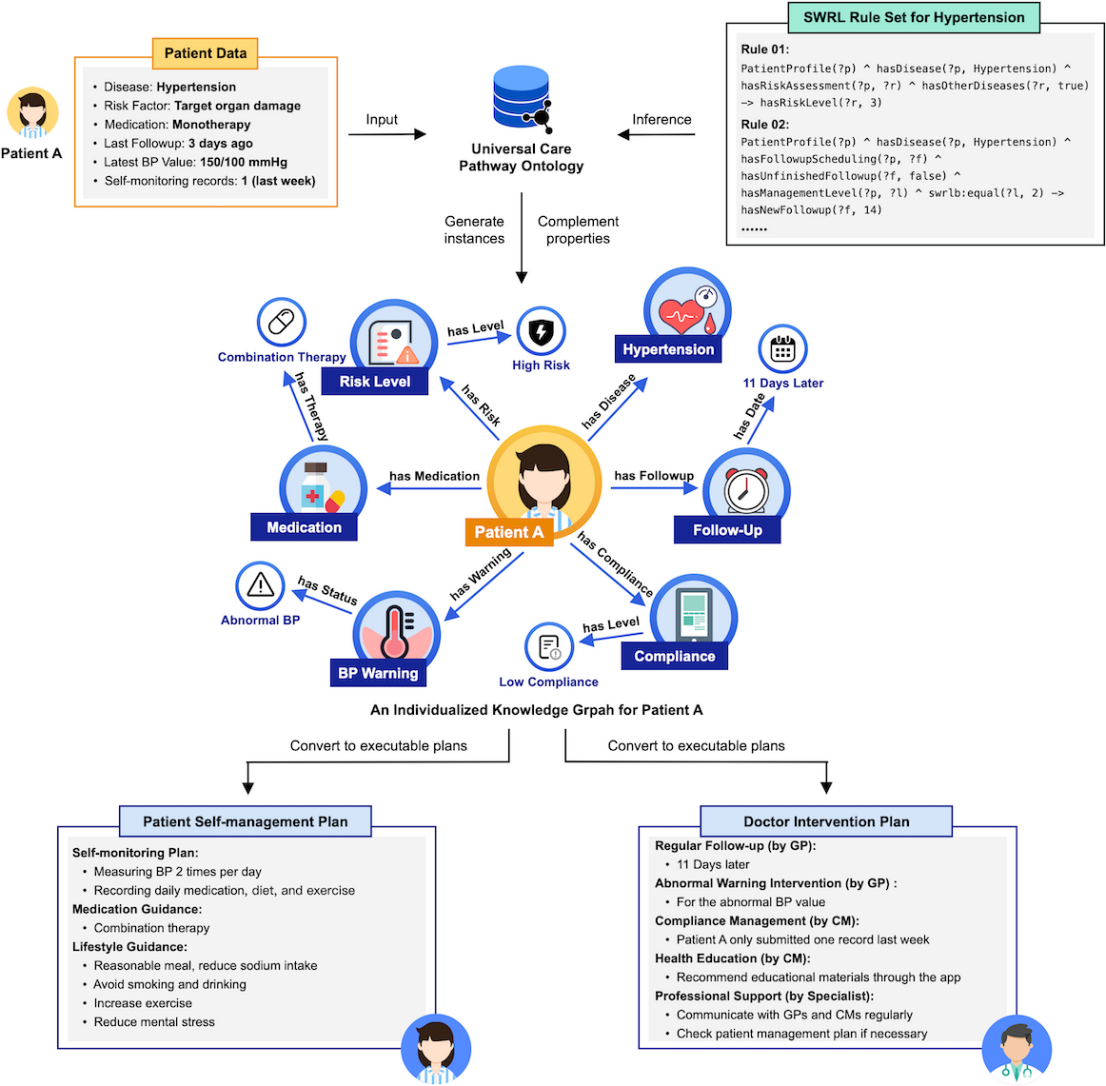
Illustrative example of the decision support process. BP: blood pressure; CM: case manager; GP: general practitioner; SWRL: Semantic Web Rule Language.

In the second phase, we incorporated medical knowledge of the model using class instances combined with rule-based reasoning. In this study, semantic web rule language (SWRL) [42] rules were utilized to perform the complex deductive inference required for decision support. The detailed description of UCPO is provided in Multimedia Appendix 2. Based on the basic UCPO and predefined SWRL rule set, a small-scale knowledge graph would be generated to incorporate patient data into UCPO for decision support. Figure 4 shows an illustrative example of the decision support process. First, patient data (Patient A in this example) are extracted from the database and then transmitted anonymously to the UCPO to generate instances of classes. Second, according to the disease type of Patient A, the corresponding rule set of the disease would be invoked to make an inference on properties of specific instances. By combining the related instances with the inference results, an individualized knowledge graph for Patient A would be established, which contains various tasks following the corresponding care pathway. Finally, the generated tasks would be converted to executable management plans, including the doctor intervention plan and patient self-management plan, via an independent rule set. The doctor intervention plan mainly involves a follow-up plan as well as intervention reminders for abnormal self-monitoring data and low compliance, whereas the patient self-management plan consists of a self-monitoring plan along with prescriptions for medication and lifestyle.

Several characteristics related to the above decision support process need to be mentioned. First, pathway tasks would be updated regularly at a task-specific frequency according to the patient data. A part of tasks will then be further assigned a valid duration defined by the Time Ontology [43]. Therefore, an incomplete subgraph might be established during a particular decision support process due to the different trigger timing of tasks. Moreover, the generation of one task might serve as a triggering condition for another task generation rule. Second, for patients diagnosed with multiple diseases, the rule set of each single disease would be executed separately. In such a case, an extra rule set for the corresponding MCC would be invoked to merge the management plans generated for different single diseases. Furthermore, the system would automatically deal with the potential redundancies and conflicts of properties in the merged management plan.

#### Engine Encapsulation

**Figure 5:**
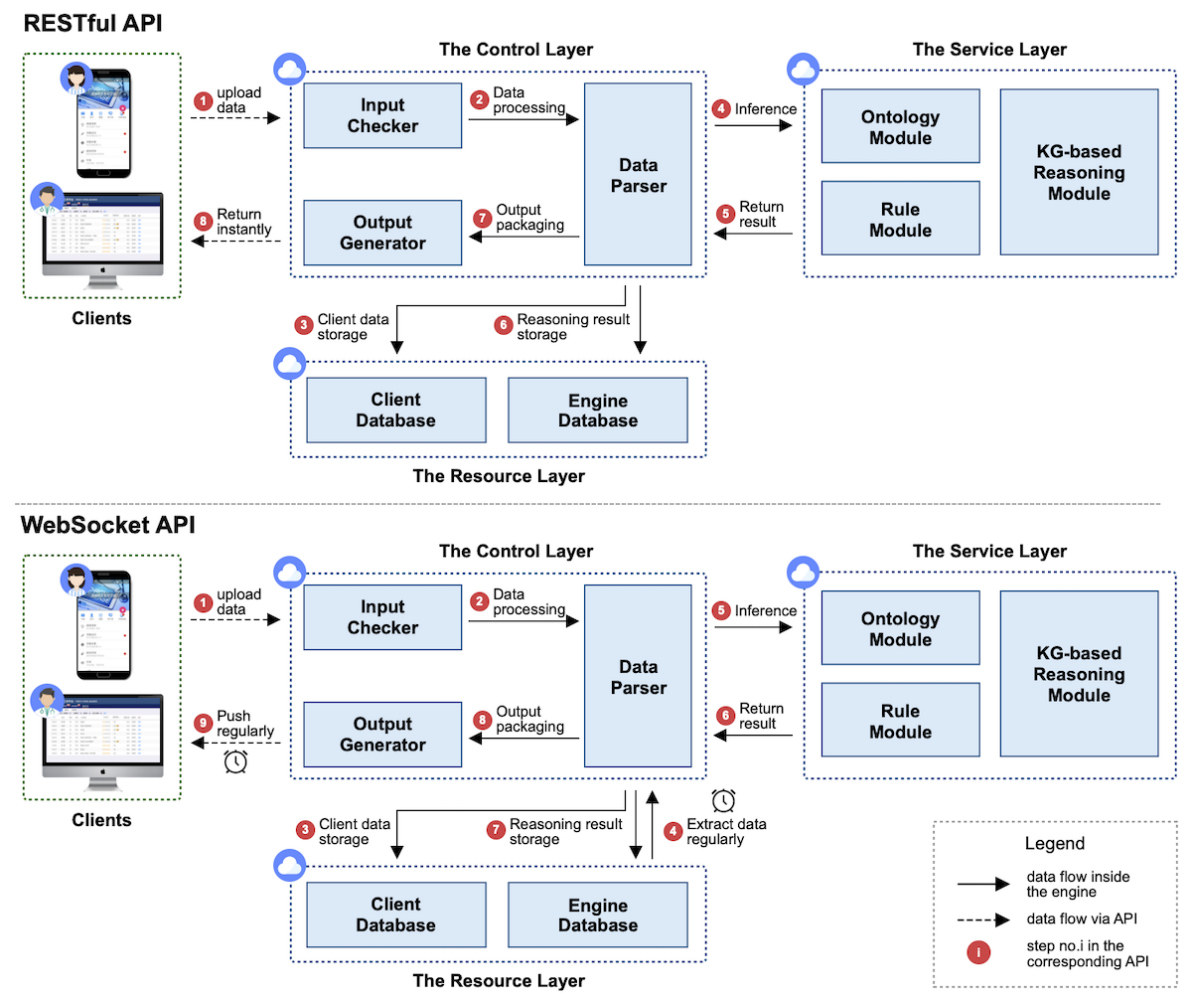
Schematic of the engine encapsulation. API: application programming interface. KG: knowledge graph.

Finally, we encapsulated the engine based on UCPO to connect to the database and interact with clients. Figure 5 shows a schematic of the encapsulation. According to the different characteristics of pathway tasks, we provided two types of web services for the engine to interact with clients: WebSocket APIs and RESTful APIs. The set of WebSocket APIs deals with the scenario for timing push notifications (eg, patient stratification), whereas the set of RESTful APIs deals with the scenario for immediate feedback (eg, abnormal condition warning). Specifically, the control layer of the engine serves as a transfer station of client data, transmitting the data to the service layer and the resource layer. Client data generally include patients’ self-monitoring data as well as intervention records from care providers, which would first be saved to the client database and then input into UCPO in different manners for different types of APIs. For WebSocket APIs, client data would be extracted regularly according to the task-specific frequency, whereas for RESTful APIs, client data would be directly transmitted into the ontology at the uploading time. All of the reasoning results would be saved in the engine database separately, with a portion of significant results also saved in the client database.

### Web Platform for Care Providers

**Figure 6:**
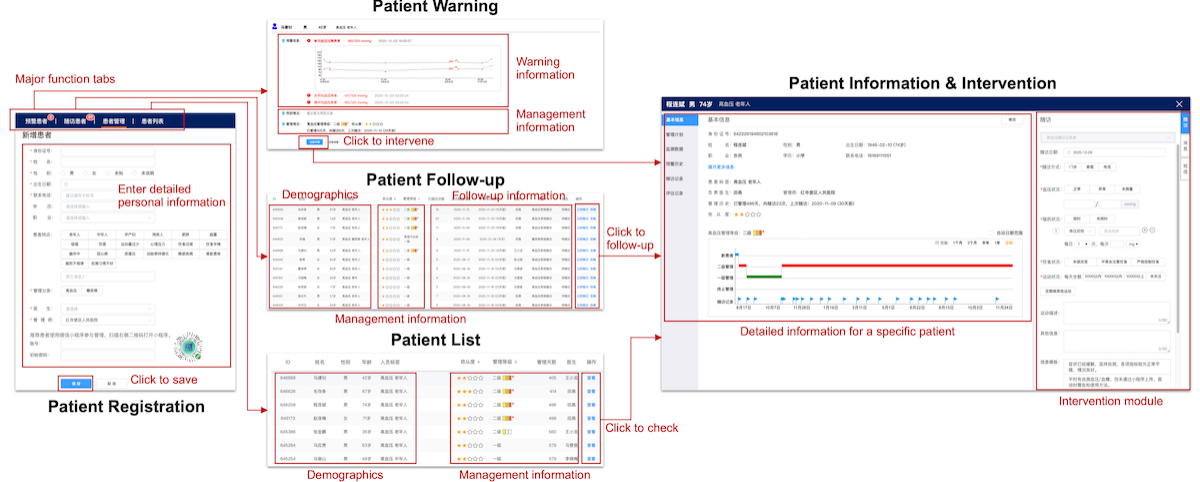
Overview of the main functions in the web platform for care providers.

The implementation of the website for care providers followed a widely used agile development methodology [44]. We iteratively added functional modules to the platform according to the proposed pathway-driven model. Figure 6 shows an overview of the main functions in the web platform. The primary users of the website are GPs and CMs. Specialists can also log in to check the status of their patients. We provided four major functional modules via tab-based navigation: patient registration, patient warning, patient follow-up, and patient list. The patient registration module is responsible for including new patients in the management, which is mainly performed by CMs. As shown in Figure 6, patients could formally receive the management after providing several types of information, including basic information (eg, demographics, phone number, ID number), disease information (eg, main disease type, associated symptoms), and the corresponding care provider information. For patients who enter the management period, the timing of intervention is determined by the other three major functional modules: (1) the patient warning module displaying all of the untreated warnings of patients’ self-monitoring data, with different types of displayed information for different types of warnings; (2) the patient follow-up module presented as a patient list in order of the next follow-up date; and (3) the patient list module, demonstrating information of patients with different diseases also in the form of a list, mainly focusing on checking compliance and searching for a specific patient.

Care providers could enter the interface of “patient information and intervention” by clicking on the corresponding buttons in the above three major functional modules. In this interface, care providers could check various types of patient information, including demographics, management plans, self-monitoring records, warning history, follow-up records, and assessment records. The platform supports three types of interventions for care providers: complete follow-up via telephone or a clinic visit, short message service text reminders, and message push via the app. Various types of templates and options are provided to simplify and normalize the intervention procedure. Moreover, care providers could edit and push educational materials to patients in another separate interface. The detailed screenshots of the web platform can be found in Multimedia Appendix 3.

### Mobile App for Patients

**Figure 7:**
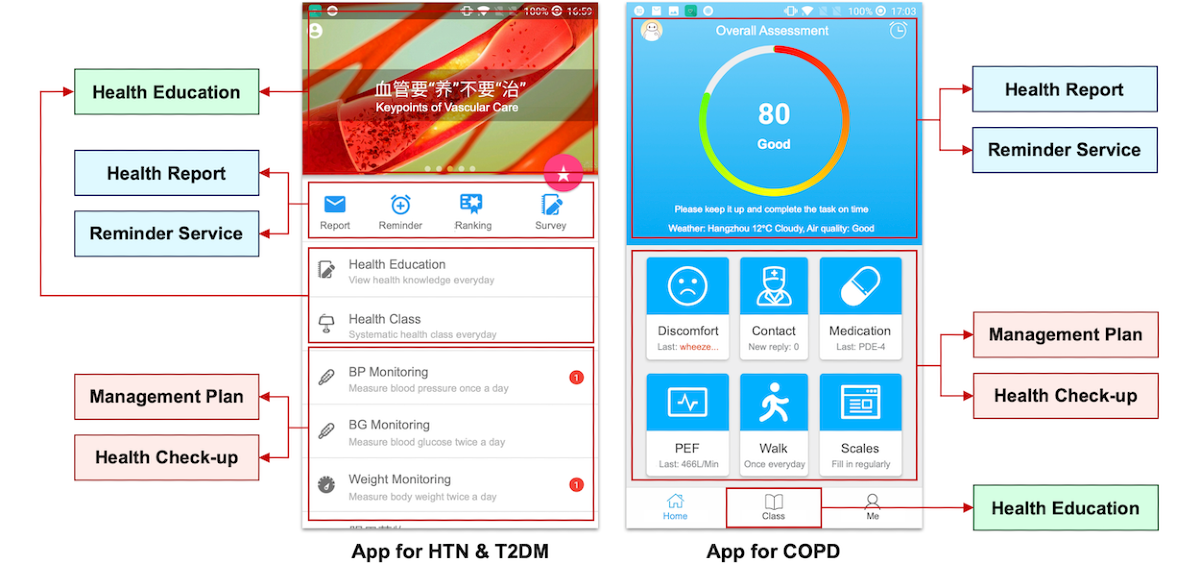
Overview of the main functions in the mobile app for patients. COPD: chronic obstructive pulmonary disease; HTN: hypertension; T2DM: type 2 diabetes mellitus; PEF: peak expiratory flow.

We utilized a goal-directed design [45] to develop the mobile app for patients. Patients were engaged in the design process to identify their needs. The concrete design process has been described in our previous study [46]; Figure 7 shows an overview of the main functions in the mobile app. Owing to the distinction between the HTN and T2DM care pathway and the COPD care pathway, we applied different user interface designs to the app for HTN/T2DM and the app for COPD. The two apps share the same underlying framework and provide similar functional modules. As shown in Figure 7, the app includes 5 major functional modules that can be accessed from the main interface: management plan, health checkup, health report, reminder service, and health education. The management plan module is the core function of the app that can be checked directly in the main interface. Currently, the initial self-management plan for patients is generated by the engine based on the management level of patients, and is then manually adjusted by care providers according to patients’ specific conditions. The management plan on the patient app is shown in the form of daily tasks along with control targets. Each task is required to be accomplished at a designated time during each day. Patients could click on the corresponding task and input the required data in a new interface. The submitted data would then be uploaded to the engine for further analysis.

The other four major functional modules were designed for patients with different needs of self-management, aiming to further improve their compliance. Concretely, the health checkup module would analyze patients’ self-monitoring data, and provide immediate and understandable feedback with the aid of the engine; the health report module would summarize the recent completion status of provided tasks and change trends in health data; the reminder service module would set reminders for the execution of daily tasks; and the health education module would display various types of educational materials selected by care providers. The detailed screenshots of the app can be found in Multimedia Appendix 4.

### Development Tools

The service engine was developed based on the Spring Boot Framework, an open-source micro service framework for the Java platform. The connected database uses MySQL 8.0, an open-source relational database management system. The clients were developed by a team of experienced programmers collaboratively. All patients, specialists, GPs, and CMs have unique IDs, and their login passwords are encrypted and kept anonymous to the database administrator.

UCPO was constructed using the Protégé 5.5.0 open-source ontology editor in W3C Web Ontology Language (OWL) standard format (second edition). We integrated UCPO into the service engine through the OWL API, a Java API implementation for manipulating OWL ontologies. For the rule-based reasoning, the SWRL Rule Engine Bridge in the SWRL API [47] was used to invoke the execution of SWRL rules through a third-party rule engine. The official implementation currently adopts the Drools rule engine owing to its good execution speed and compatibility with Java programs.

### Retrospective Study

#### Study Design

**Figure 8:**
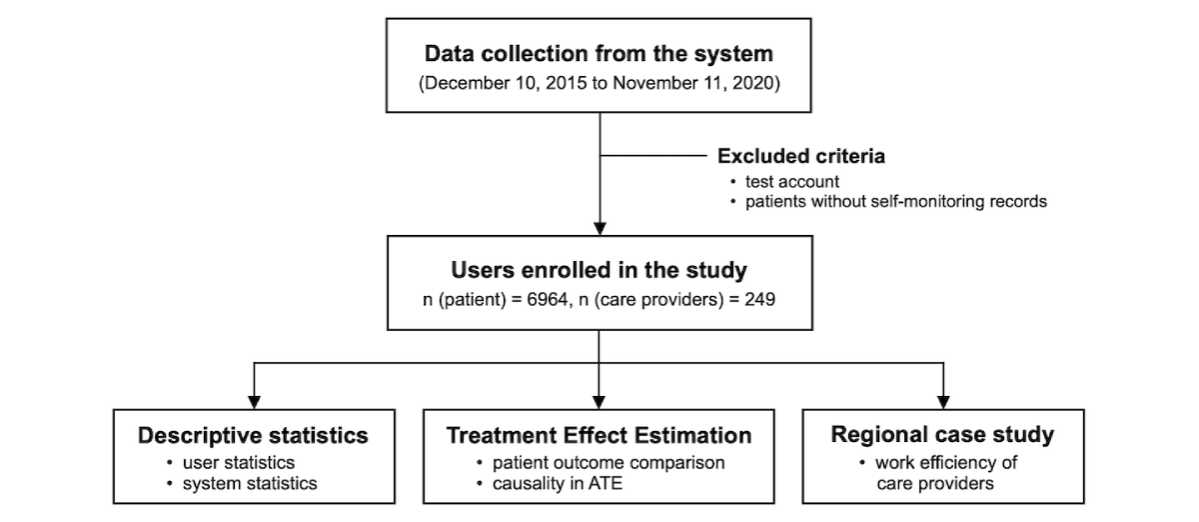
Overall retrospective study design. ATE: average treatment effect.

The first version of our system was deployed in Ningxia Hui Autonomous Region in 2015, which only supported HTN management at that time. Currently, the system supports management of three single chronic diseases (HTN, T2DM, and COPD) and one type of MCC (HTN with T2DM). To investigate the effect of our system, we collected almost all of the data generated through the system since its deployment in 2015. A retrospective analysis was then performed based on the collected data to evaluate system usability, treatment effect, and quality of care. Figure 8 illustrates the overall study design. We first screened a portion of user accounts that had no self-monitoring records or were created for testing purposes. The remaining users for retrospective analysis consisted of 6964 patients with chronic diseases and 249 care providers. We then performed three types of analyses: (1) descriptive statistical analysis for user information and system usage, (2) treatment effect estimation for patient outcome changes after receiving the management, and (3) a regional case study for understanding the work efficiency of care providers.

Specifically, for the descriptive statistics, we mainly analyzed patient usage of the system and decision support abilities of the engine. For the treatment effect estimation, physiological indices from patients’ self-monitoring data were selected as patient outcomes to evaluate the treatment effect. We first compared the change of patient outcomes over different time spans from a traditional statistical perspective, and then estimated the average treatment effect (ATE) from a causal perspective. For the case study, we selected several primary care institutions in different districts of Ningxia Hui Autonomous Region to evaluate the work efficiency of care providers over a long-term horizon. Owing to the limitation of data acquisition, we only analyzed the work efficiency of GPs for patients with HTN and diabetes from two aspects: the frequency of follow-ups in one day and the handling time of a follow-up request. The frequency of follow-ups in one day demonstrates how our system reduces the time cost of a single follow-up, whereas the response days of a follow-up request represents the time duration before a generated follow-up request is handled.

#### Informed Consent and Ethical Considerations

Patients registered in the telehealth system have signed inform consent forms for accessing and using their personal data. The care providers signed informed consent forms as well. All procedures were performed in accordance with the ethical guidelines for biomedical research involving human subjects at Ningxia Medical University.

#### Data Analysis

Python 3.7 was used for data preprocessing, including data extraction from the database and descriptive analysis. Statistical analysis was performed using SPSS version 23.0. A paired Student *t* test was used for analyzing changes in patient outcomes. All statistical tests are reported at a two-sided significance level of 5%. For the causal inference between pathway-driven intervention and patient outcomes, we adopted DoWhy—an end-to-end Python library—to estimate the causal effect of our intervention [48]. In short, DoWhy follows four key steps to perform causal inference: (1) model the problem as a causal graphical model based on user-defined assumptions; (2) identify a desired causal effect estimand based on the model; (3) estimate the identified causal effect using statistical methods such as matching or stratification; and (4) verify the validity of the estimate using a variety of robustness checks. In this study, we adopted two classic causal inference methods to estimate ATE: propensity score matching (PSM) [49,50] and propensity score stratification (PSS) [51]. Both methods utilize propensity scores to achieve comparability of treatment groups and control groups in terms of their pretreatment covariates, thereby eliminating confounding bias in estimating treatment effects [52].

## Results

### Descriptive Statistics

#### User Statistics

**Figure 9:**
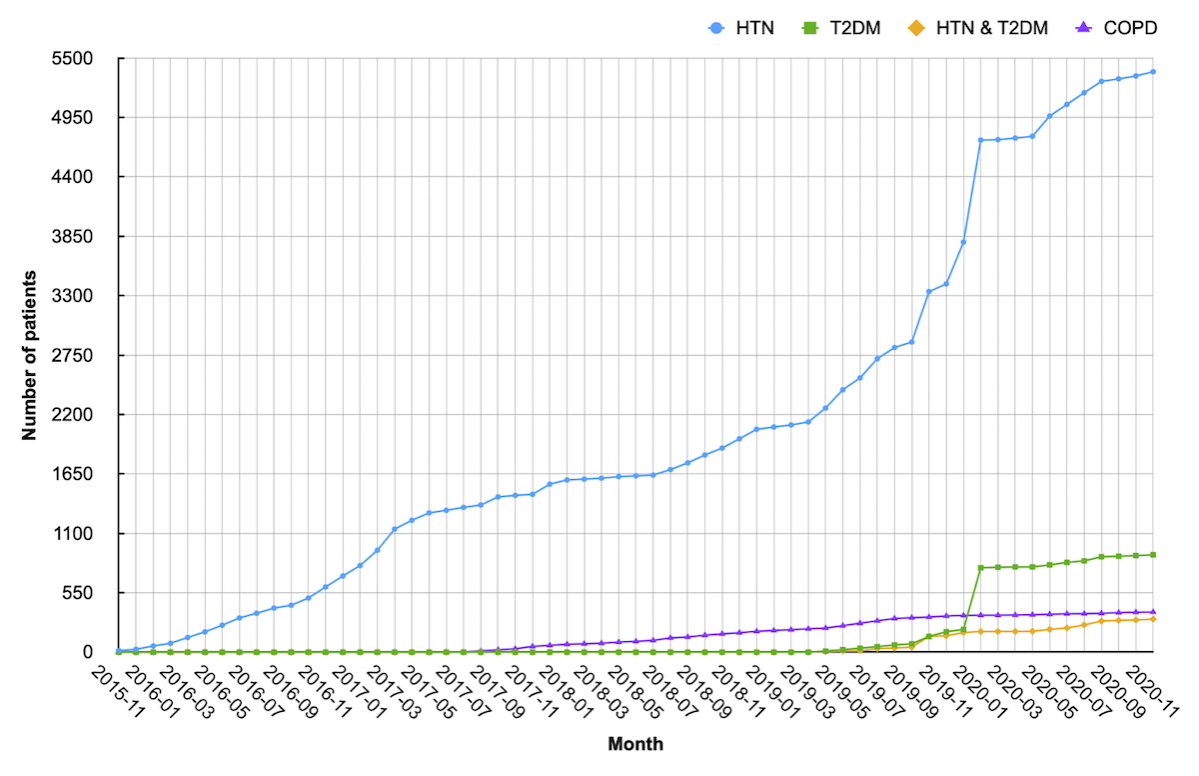
Changing trends in the number of patients with different diseases over time. COPD: chronic obstructive pulmonary disease; HTN: hypertension; T2DM: type 2 diabetes mellitus.

As described above, since its deployment in 2015, a total of 6964 patients with chronic diseases and 249 care providers have registered in our system and actually used the system. Table 2 summarizes the demographics of patients and the detailed information of care providers. The average age of the patients was 58 years. Among the 6964 patients, 55.41% (n=3859) reported a relatively low educational level (high school and below), and only approximately 20% had a college degree or above; one-quarter of the patients did not provide their educational attainment at the time of registration. In terms of disease type, a substantial proportion of enrolled patients (81.7%) were diagnosed with HTN, the majority of whom had HTN alone with the remaining patients having coexisting T2DM. The other patients had a clinical diagnosis of T2DM (not with HTN) or COPD. The changing trends in the number of patients with different diseases over time are shown in Figure 9. The care providers consisted of 56 specialists, 107 GPs, and 86 CMs from different departments in different levels of hospitals.

Table 3 provides simple descriptive summary statistics of patients’ self-monitoring data. Patients could submit various types of records through the mobile app, mainly including physiological indices, lifestyle records, medication, and discomfort. Physiological indices included blood pressure (BP, together with heart rates) for patients with HTN, blood glucose (BG) for patients with diabetes, and peak expiratory flow (PEF) for patients with COPD. All three indices could be measured at home via different devices [53-55]. Patients who had medication orders were required to record their medications regularly. Patients with HTN and/or T2DM were recommended to record their daily diet and exercise. For patients with COPD, psychological conditions were monitored through several validated scales [56,57]. From the statistical results, medication records and BP records were the most frequently submitted data by patients using the system. Moreover, for patients with different diseases, the emphasis of their records was also different, as shown in Table 4.

**Table 2:** Patient demographics and care provider information (N=6964).

Characteristics				Value
**Patient demographics**				
	**Sex, n (%)**			
		Male	3973 (57.1)	
		Female	2991 (42.9)	
	Age (years), mean (SD)			58 (12.3)
	**Educational level, n (%)**			
		Secondary school and below	2988 (42.9)	
		High school	871 (12.5)	
		Graduate and above	1388 (19.9)	
		Unknown	1717 (24.7)	
	**Disease type, n (%)**			
		Hypertension (single)	5384 (77.3)	
		Type 2 diabetes (single)	901 (12.9)	
		Hypertension with type 2 diabetes	306 (4.4)	
		COPD^a^	373 (5.4)	
**Care provider information**				
	**Position, n (%)**			
		Specialist	56 (22.5)	
		General practitioner	107 (43)	
		Case manager	86 (34.5)	
	**Department, n (%)**			
		Cardiology	28 (11.3)	
		Endocrinology	15 (6.0)	
		Pneumology	13 (5.2)	
		General practice	193 (77.5)	

^a^COPD: chronic obstructive pulmonary disease.

**Table 3:** Descriptive statistics of patients’ self-monitoring data through the system.

Data type		Patients, N		Records, N
**Physiological indices**				
	Blood pressure		6379	139,234
	Blood glucose		1195	4599
	Peak expiratory flow		119	9511
**Lifestyle records**				
	Diet		316	46,246
	Exercise		1430	50,721
	Psychological status		274	11,900
Medication		2260		222,055
Discomfort		493		35,332
Total counts		6964		519,598

**Table 4:** Self-monitoring data for patients with different diseases.

Data type		Records for patients with different diseases, n (%)						
		HTN^a^		T2DM^b^		HM^c^		COPD^d^
**Physiological indices**								
	Blood pressure	135,512 (36.4)	2299 (42.1)		1423 (47.0)		0 (0)	
	Blood glucose	480 (0.1)	3003 (55)		1116 (36.8)		0 (0)	
	Peak expiratory flow	0 (0)	0 (0)		0 (0)		9511 (6.8)	
**Lifestyle records**								
	Diet	46,189 (12.4)	30 (0.5)		27 (0.9)		0 (0)	
	Exercise	39,979 (10.8)	14 (0.3)		18 (0.6)		10,710 (7.7)	
	Psychological status	0 (0)	0 (0)		0 (0)		11,900 (8.5)	
Medication		149,291 (40.2)		102 (1.9)		441 (14.6)		72,221 (51.8)
Discomfort		335 (0.1)		8 (0.1)		5 (0.2)		34,984 (25.1)

^a^HTN: hypertension.

^b^T2DM: type 2 diabetes mellitus.

^c^HM: Hypertension with type 2 diabetes mellitus.

^d^COPD: chronic obstructive pulmonary disease.

#### System Statistics

Table 5 presents an overview of intervention records through the system following the four different care pathways. From the perspective of engine workflow, we classified the records in accordance with the proposed 9 common tasks (diagnosis was not involved in the system) into three categories: automatic evaluation, patient self-management support, and care provider intervention. Automatic evaluation included risk assessment and hierarchical management, which would be automatically calculated by the engine. Patient self-management support included lifestyle guidance and medication guidance, which were initially formulated by the engine and can be adjusted by care providers through the system (ie, the self-management plan). Care provider intervention included regular follow-up and abnormal condition intervention by GPs, as well as compliance management and health education by CMs. The engine would automatically schedule the follow-ups and detect the abnormal condition or low compliance, and then care providers would need to contact patients through the system to deliver the actual intervention. Health education was performed in the form of electronic materials on the patient app.

From the statistical analysis, the engine was able to generate different types of records regularly according to the specific care pathway. The content and frequency of each task were different for different diseases. For example, patients with COPD would directly be classified based on the risk evaluation results without an extra classification task. For medication guidance, we only counted the medication adjustment records generated by the engine. Moreover, medication guidance for patients with COPD have not yet been incorporated into the engine (conducted manually by care providers). Since patient compliance was updated every day by the engine, the number of records was relatively larger than that for other types of records. In terms of health education, we counted the number of articles and videos that can be viewed on the patient app [58]. Notably, several types of interventions for the COPD care pathway only involved a small number of patients due to relatively late deployment of relevant functional modules.

**Table 5:** Descriptive statistics of intervention records through the system following different care pathways.

Intervention type		Patients receiving intervention, N					Records generated by the engine, N			
		HTN^a^	T2DM^b^	HM^c^	COPD^d^	HTN		T2DM	HM	COPD
**Automatic evaluation**										
	Risk assessment	3372	841	306	74	3933		912	788	4615
	Hierarchical management	5383	901	306	NA^e^	301,735		6356	15,287	NA
**Patient self-management support**										
	Lifestyle guidance	5376	901	306	30	20,459		1851	1417	1065
	Medication guidance	1381	609	69	NE^f^	5200		2048	490	NE
**Care provider intervention**										
	Regular follow-up	5322	892	304	339	18,217		2983	2064	1621
	Abnormal condition intervention	1657	60	94	59	8385		218	318	2317
	Compliance management	5379	900	306	30	2,099,894		274,718	175,538	6930
	Health education	5384	901	306	373	199		199	199	115

^a^HTN: hypertension.

^b^T2DM: type 2 diabetes mellitus.

^c^HM: Hypertension with type 2 diabetes mellitus.

^d^COPD: chronic obstructive pulmonary disease.

^e^NA: Not applicable in the current pathway.

^f^NE: Not currently incorporated into the engine.

### Treatment Effect Estimation

#### Patient Outcome Comparison Over Multiple Time Spans

**Figure 10:**
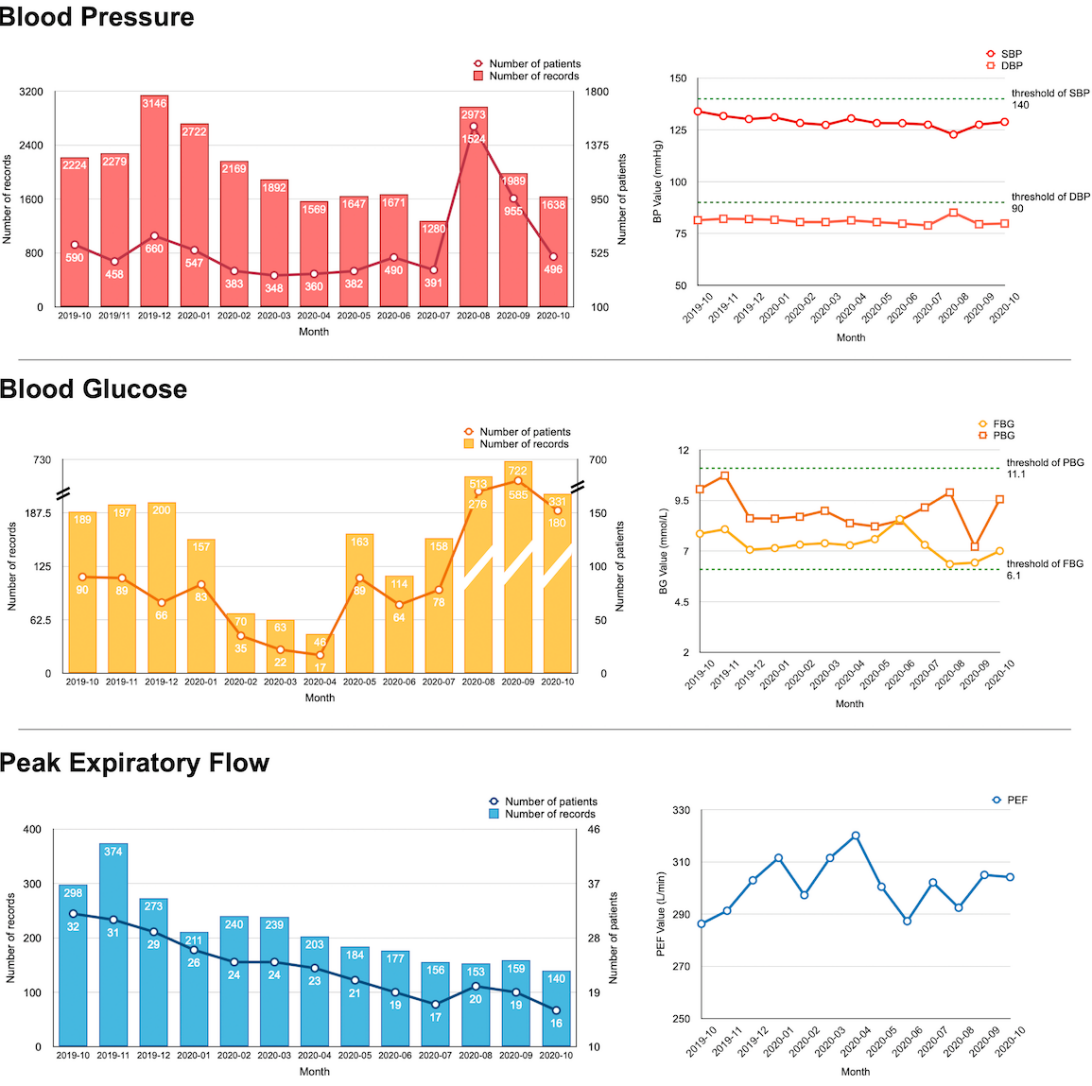
Monthly records of patient outcomes from October 2019 to October 2020. DBP: diastolic blood pressure; FBG: fasting blood glucose; PBG: plasma blood glucose; PEF: peak expiratory flow; SBP: systolic blood pressure.

Self-measured physiological indices submitted by patients were regarded as patient outcomes to evaluate the effect of our system. Figure 10 shows the monthly records of different patient outcomes during the most recent year. For BP, both systolic BP (SBP) and diastolic BP are presented; for BG, the system provided two options for BG self-monitoring: fasting blood glucose (FBG) and postprandial blood glucose. Compared with BP, the numbers of records for BG and PEF were relatively small due to the large proportion of patients with HTN in our system. From the trends of monthly mean value of these indices, the BP value remained basically stable at a normal level, whereas BG and PEF values fluctuated within a certain range.

We then compared the change of patient outcomes over different time spans (from 1 month to 1 year). The mean value of an outcome for a specific patient before and after a time span was calculated based on the submitted records at the first 2 weeks when the patient was enrolled and the 2 weeks after the specific time span. In terms of BP and BG values, we only analyzed SBP and FBG. From the comparison tests shown in Table 6, there were significant differences in the change of SBP values over all time spans, as well as a change of FBG values over 2 months. The change of FBG values over 1 month to 4 months showed a nonstatistically but clinically considerable decrease. No significant difference was found for the change of PEF values in these comparisons. A detailed subgroup analysis on patients with different diseases and in different age or gender groups is provided in Multimedia Appendix 5.

**Table 6:** Comparison of patient outcomes over different time spans.

Patient outcome		Patients who have records, N	Mean value before	Mean value after	*P* value
**SBP^a^ (mmHg)**					
	30 days	1140	132.13	128.43	<.001
	60 days	1263	128.77	125.78	<.001
	90 days	1008	130.52	128.14	<.001
	120 days	725	132.56	128.86	<.001
	150 days	446	130.8	127.6	<.001
	180 days	403	130.94	128.18	<.001
	360 days	214	130.44	128.23	.02
**FBG^b^ (mmol/L)**					
	30 days	76	8.34	6.76	.32
	60 days	457	5.53	4.92	.045
	90 days	58	6.74	6.58	.31
	120 days	28	7.17	6.77	.24
	150 days	12	6.94	7.38	.52
	180 days	10	7.15	6.78	.55
	360 days	10	7.08	7.11	.95
**PEF^c^ (L/min)**					
	30 days	55	315.78	320.59	.67
	60 days	47	320.51	323.13	.84
	90 days	44	325.77	310.69	.98
	120 days	45	326.61	327.76	.95
	150 days	40	316.67	309.55	.73
	180 days	41	314.81	311.16	.86
	360 days	29	318.3	299.04	.51

^a^SBP: systolic blood pressure.

^b^FBG: fast blood glucose.

^c^PEF: peak expiratory flow.

#### Causality in ATE

**Figure 11:**
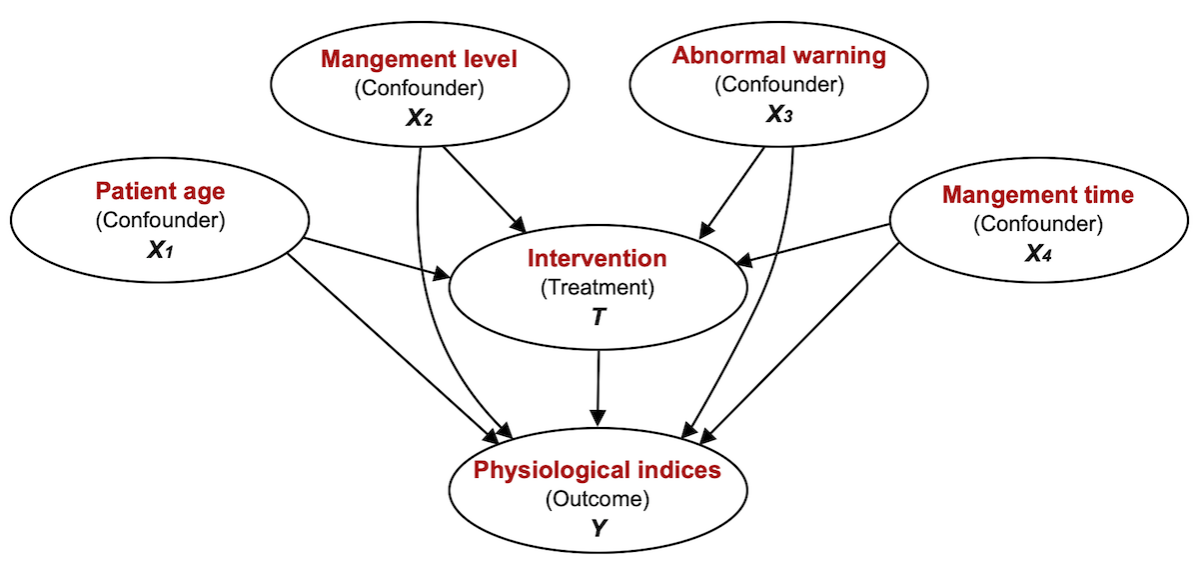
Causal graphical model for average treatment effect.

For the observational data, treatment effect estimation may be affected by the potential existing confounders. A confounder is a type of covariate that affects both the treatment assignment and the outcome. Spurious effect and selection bias are two main challenges brought about by confounders [59]. To estimate the true treatment effect behind our intervention, we utilized several causal inference methods to eliminate the influence of confounders. Concretely, we first constructed a causal graphical model for our problem based on prior knowledge (confirmed by physicians), as shown in Figure 11. Four confounders were considered in this study: patient age, management level, abnormal warning, and management time. The treatment variable was the intervention performed by care providers, mainly including regular follow-up and abnormal condition interventions. The outcome variables were physiological indices submitted by patients, including SBP, FBG, and PEF.

Based on the causal graph, we extracted a subdataset specifically for causal inference. For the treatment group (ie, T=True), we selected the mean value of patient outcomes within 1 month after receiving the intervention as the potential outcome (ie, Y), whereas for the control group (ie, T=False), we extracted the records of patients who did not receive any intervention within 2 weeks and regarded the mean value of self-monitoring records as the outcome. For the confounders, “management level” was the latest level of the patient at the initial time point of the record, “abnormal warning” was a Boolean variable demonstrating whether the patient has reported any abnormal condition during the corresponding period of the record, and “management time” was the time since the patient started to receive the management. The sizes of the screened dataset for the three types of patient outcomes are listed in Table 7.

Subsequently, two propensity score-based methods were adopted for evaluating the ATE, namely PSM and PSS. The estimated results are also presented in Table 7. The value of the causal estimate represents the change in the outcome value when performing the intervention (ie, if we change the treatment from “False” to “True,” then the outcome value will change by the value of “estimate”). A positive value means that the outcome increases with treatment, whereas a negative value means that the outcome decreases with treatment. As expected, the values of SBP and FBG decreased significantly after receiving the intervention. The value of PEF increased after the intervention, which might be interpreted as an improvement in pulmonary function. Further, we utilized multiple refutation methods to validate the obtained estimates, which confirmed that our assumptions and results were reliable. The detailed results of refutation can be found in Multimedia Appendix 6.

**Table 7:** Causal inference with propensity score matching (PSM) and propensity score stratification (PSS) for different patient outcomes.

Patient outcome	Records for inference, N	Estimated ATE^a^	
		PSM	PSS
SBP^b^ (mmHg)	20,535	–5.24	–5.51
FBG^c^ (mmol/L)	1765	–1.82	–1.27
PEF^d^ (L/min)	1196	10.08	2.36

^a^ATE: average treatment effect.

^b^SBP: systolic blood pressure.

^c^FBG: fast blood glucose.

^d^PEF: peak expiratory flow.

### Regional Case Study

**Figure 12:**
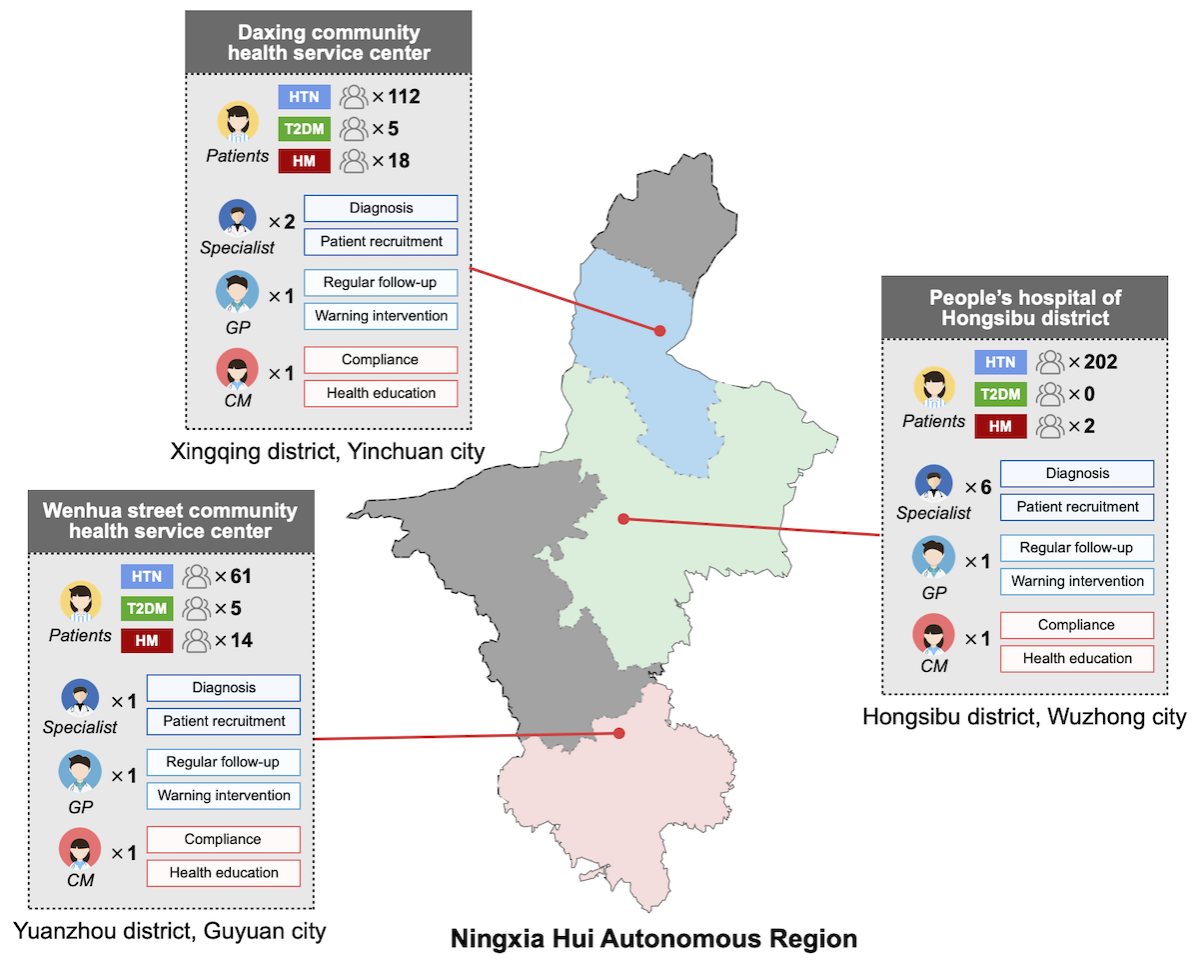
Overview of the selected three primary health care facilities in different districts of Ningxia. HTN: hypertension; T2DM: type 2 diabetes mellitus. HM: hypertension and diabetes.

We selected three primary health care facilities that registered in our system at the same time (in the first half of 2019) from different districts of Ningxia Hui Autonomous Region. All three institutions were staffed with one GP and one CM to participate in the management. Moreover, several specialists from the nearest secondary or tertiary hospitals aided with patient diagnosis and recruitment. Figure 12 shows an overview of the selected three facilities. Patients mainly comprised those with HTN, with a small proportion of patients with diabetes. Patients with COPD were not included in these three institutions.

**Figure 13:**
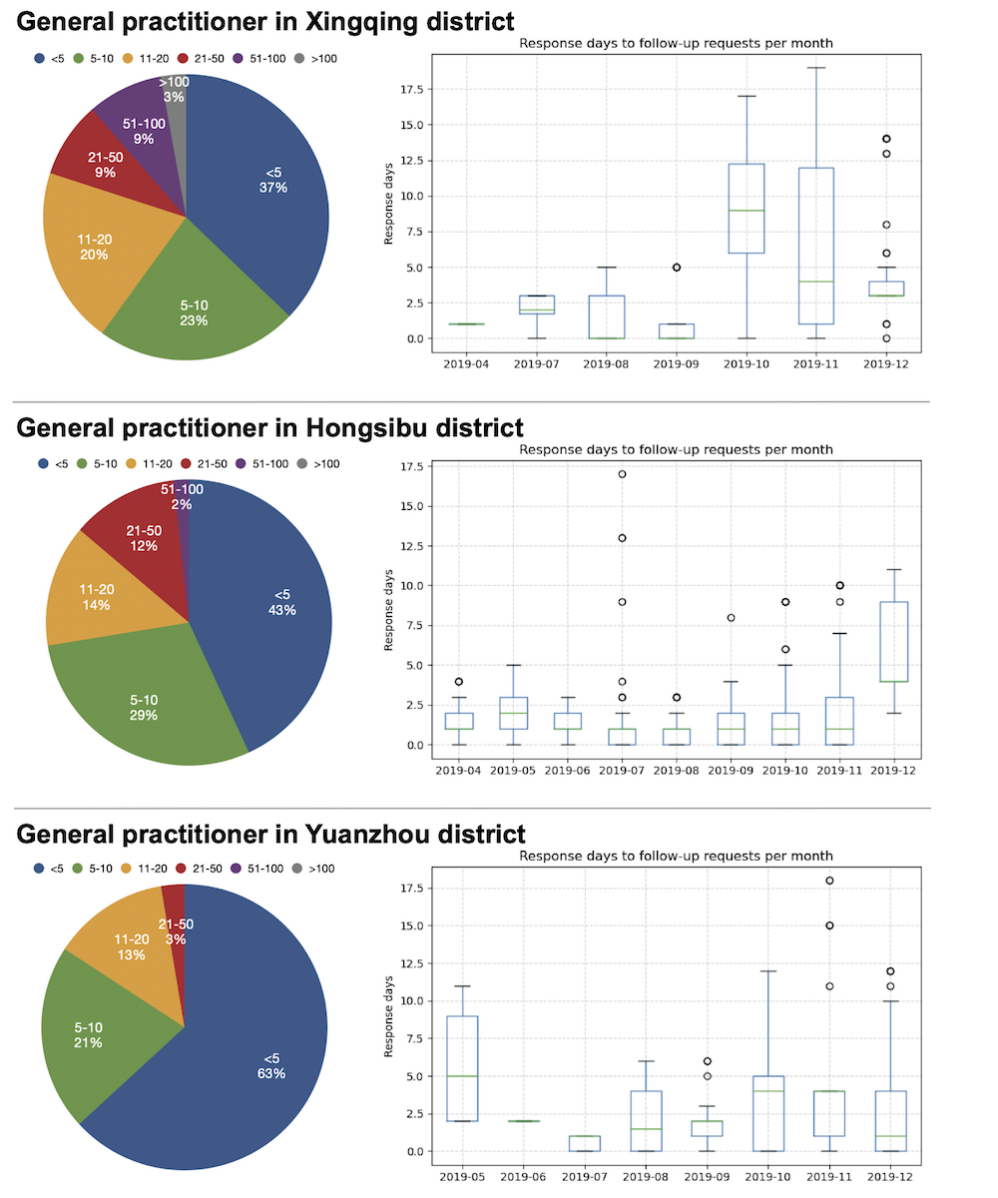
Frequency of follow-ups in one day and response days to a follow-up request for the three general practitioners.

GPs and CMs worked collaboratively to perform pathway-driven management through the system. Table 8 presents the descriptive statistics of the records of interventions performed by GPs and CMs. Further, we calculated the work efficiency of GPs based on their regular follow-up records, as shown in Figure 13. The pie chart demonstrates the percentage of different numbers of follow-up records in a day, whereas the box plot presents the distribution of response days to a follow-up request per month. From the pie charts, all three GPs performed less than 20 follow-ups in over 80% of the follow-up days, and the average number of follow-ups in a day was 16.4, 10, and 5.3, respectively. From the box plots, almost all of the medians of monthly response days were maintained within 5 days, with a couple of outliers in several months. As time progressed, the trends of response days differed for different GPs. Due to the pandemic outbreak of COVID-19 in China in early 2020 [60], for this case study, we only evaluated the intervention records in 2019 to ensure credibility of the results.

**Table 8:** Descriptive statistics of records of interventions performed by care providers in the selected three institutions.

Institution location		Regular follow-up (GP^a^), N	Warning intervention (GP), N	Compliance (CM^b^), N
**Xingqing district**				
	Patients	116	114	82
	Records	306	148	104
**Hongsibu district**				
	Patients	196	48	103
	Records	54	149	118
**Yuanzhou district**				
	Patients	69	73	53
	Records	203	301	86

^a^GP: general practitioner.

^b^CM: case manager.

## Discussion

### Principal Findings

In this study, we designed and implemented a coordinated telehealth system that supports the management of multiple chronic diseases based on a tailored integrated care model for the emerging “three-manager” mode in China. The system could provide pathway-driven decision support throughout the management process via an ontology-based approach. According to the retrospective analyses on long-term system usage data, our system was able to link patients’ self-management to care providers’ interventions through semiautomatic decision support following the predefined care pathway. Furthermore, patient outcomes showed a certain degree of improvement after receiving management through the system.

Several interesting aspects can be found from the system evaluation results. First, in terms of patients’ self-monitoring data, the different emphasis of records for patients with different diseases reflects the focus of out-of-hospital management regimens on different chronic conditions. The management regimen of HTN focuses on lowering BP through medication and lifestyle changes simultaneously, whereas the management regimen of T2DM tends to focus first on lifestyle intervention instead of medication. Moreover, the management regimen of COPD requires persistent medication and close attention to acute exacerbation and mental condition of patients.

Second, in terms of comparison tests, the different levels of statistical significance among patient outcomes could be mainly attributed to the difference in cardinality among these three types of records. Compared to patients with diabetes or COPD, patients with HTN constituted the majority of the registered patients. Moreover, the self-measurement of BG at home was slightly more complicated than that for BP due to its invasiveness [61], which would potentially lower patient compliance. The self-monitoring of PEF relied on having a peak flow meter that has not been massively promoted for patients with COPD in China. Furthermore, from a clinical viewpoint, both the SBP and FBG values showed an expected decrease over most time spans, whereas the PEF value only increased over short time periods (30 days and 60 days). We considered that the relatively large SDs for the PEF value and the irreversibility of pulmonary function for patients with COPD might account for the absence of differences.

Third, in terms of causal effect estimation, the estimated ATE for SBP and FBG was consistent with the comparison tests (decreased after intervention), and the results derived by PSS and PSM were similar. The SBP value showed a relatively greater change than the FBG value under causality assumptions. By contrast, the estimated effect of PEF by the two causal inference methods showed a great degree of dissimilarity (a part of refutation tests for PEF also showed sensitivity). A possible explanation for this is that the distribution of outcomes and selected confounders among patients with COPD had a large discrepancy, which led to different intermediate results when performing stratification and matching. Moreover, certain bias might exist in the extraction strategy itself for causal data. Therefore, the true effect of our intervention for patients with COPD remains a question for further investigation. In another prospective study, we found an improvement in COPD-specific quality of life and mental health status of patients after 6-month pathway-driven management, with no significant difference in the mean PEF value [62].

Fourth, according to the regional case study, the system was able to generate different intervention tasks based on patients’ health status, and then assigned them to the corresponding care providers. In this case study, the intervention tasks referred to regular follow-up and abnormal condition intervention performed by GPs, as well as compliance intervention performed by CMs. GPs and CMs were able to work with each other to provide comprehensive management support for patients. In terms of work efficiency, intuitively, the more follow-ups care providers perform in one day and the less handling time a follow-up request costs, the more effective their work will be. However, in practice, considering the workload and work arrangement of care providers, we believe that for one care provider, approximately 10 to 20 follow-ups in a single day and response within one work week to a follow-up request are reasonable, and can guarantee the quality and timeliness of follow-up. According to these criteria, the GP in Hongsibu district did a good job from both aspects, whereas the GP in Xingqing district had a relatively long duration of response to follow-up requests in the last few months of 2019. The GP in Yuanzhou district had a small average number of follow-ups in a single day, which might be attributed to the small cardinality of patients compared with the other two regions.

### Comparison With Prior Work

To better delineate the contribution of this study, we compared our work with prior research from multiple aspects. In terms of model construction, the integrated care model proposed in this study can be considered as an individual-level customization of the well-known chronic care model (CCM) [63-66]. Interventions that incorporated one or more elements of the CCM have shown benefits for primary care outcomes, with large effect sizes for self-management support, delivery system design, and decision support [2]. The core of our model is the management plan combined with pathway-driven coordinated intervention, which adequately represent the above three elements. Further, the system itself is an implementation of the clinical information system in the CCM. In addition, several elements of other models can be found in our system, such as a complete eHealth-based feedback loop between patients and care providers mentioned in the eHealth Enhanced Chronic Care Model [10], effective use of health care personnel mentioned in the Innovative Care for Chronic Conditions model [67], and utilization of remote patient monitoring mentioned in the Transitional Care model [68,69].

We then compared our research with 4 prior studies that explored the comanagement of multiple chronic diseases using information technologies. The results are shown in Table 9. Among these studies, three ([16,17] and our study) utilized ontology to provide decision support abilities during the care process. Four of the studies ([16-18] and ours) supported the management of MCC through different mechanisms. Three studies ([18,19] and ours) designed an individual platform for both care providers and patients, respectively. In terms of evaluation, Riaño et al [16], Lasierra et al [17], and Laleci et al [18] only performed a technical evaluation or pilot application on their solutions, whereas Omboni et al [19] and our study deployed the system in a real-world setting for a relatively long period to test the effectiveness. In addition, due to the limitations of labor and time costs, our system currently only supports three types of chronic conditions and one type of MCC.

**Table 9:** Comparison of recent studies using information technologies on comanagement of multiple chronic diseases.

Study	Country	Target users	Technology for decision support	Disease types	Approach for management of MCC^a^	System implementation	Evaluation
This study	China	Patients and care providers	Ontology-based rule reasoning driven by the care pathway	3	Automatic integration via manually formulated extra rule set	Full-featured telehealth system (with mobile app)	Multidimensional retrospective study
Riaño et al [16]	Italy	Care providers	Case profile ontology combined with SDA^b^ diagram	19	Semiautomatic integration of several individual plans	Wrapper system integrated into the K4CARE project	Technical evaluation and ground test involving health care professionals
Lasierra et al [17]	Spain	Patients	Ontology-driven patient profile specification	11	Manual specification of multichronic patient profiles	Semantic autonomic agent prototype	Technical evaluation without end users
Laleci et al [18]	Spain, Sweden, and United Kingdom	Patients and care providers	Decision logic encoded in GDL^c^ version 2	4	Manually designed reconciled rules	C3-Cloud web platform for both MDT^d^ and patients	Usability studies involving patients and clinicians
Omboni et al [19]	Italy	Patients and care providers	Analysis algorithms for generating a medical report	4	Not mentioned	Web-based telehealth platform in the context of IoMT^e^ (with mobile app)	Different observational studies in various settings

^a^MCC: multiple chronic conditions.

^b^SDA: state-decision-action.

^c^GDL: Guideline Definition Language.

^d^MDT: multidisciplinary care team.

^e^IoMT: Internet of medical things.

Compared with these prior studies, our study was innovative from several aspects. To the best of our knowledge, this study is the first to construct a theoretical model for care delivery under the “three-manager” mode in China. The proposed model utilizes the concept to address the challenges of the limited ability of GPs and CMs in the primary care setting. Through refinement of a universal care pathway and specification on different chronic conditions, care providers from primary health care facilities were able to perform effective management following the practice of evidence-based medicine. Further, our model fully embodies the characteristics of coordination and a “closed loop.” Conclusively, the coordination was mainly reflected in the cooperation of different management roles and inherent associations among different pathway tasks (eg, the frequency of regular follow-up is determined by the results of hierarchical management). The “closed-loop” feature was reflected in the feedback mechanism between patients and care providers, which was implemented via dynamically adjustable management plans with the aid of information technologies.

Based on the constructed model, we implemented a telehealth system that is highly applicable for practical deployment in Chinese rural areas. The system has been carefully designed with comprehensive functions and a user-friendly interface. Care providers and patients can easily grasp the operational methods of the system after brief training. Moreover, we evaluated our system through a multidimensional retrospective study. Long-term observational data from the real world were utilized to investigate the effect of our system from several aspects, including system usability, clinical validity, and quality of care.

### Strengths and Limitations

Our study has several strengths. First, in terms of system implementation, we provided two forms of mobile apps for patients: the native app and the WeChat mini program. In practical use, we found that compared with the native version, the WeChat mini program did not require installation and was easy to access from WeChat, which is one of the most frequently used apps in China. A majority of enrolled patients (especially elderly patients) tended to use the mini program, which we believe might potentially improve their compliance. Second, benefitting from the design of the universal care pathway, the system can be readily generalizable to other chronic diseases through modification of concrete task contents and definition of the corresponding rule set. The backend service and user interface also need to be updated to complete the full extension of the system. Third, we explored a new approach for evaluating patients’ long-term management effect based on causal inference methods. Although the methods and assumptions adopted in this study were preliminary, we believe that compared with simple comparison tests, the evaluation methods from a causal perspective might be more appropriate for long-term observational data directly extracted from the real world instead of clinical trials. Fourth, to evaluate the quality of care under computer-based management, we proposed a simple assessment method based on the timing and numbers of follow-ups according to our previous study [70]. The proposed method is a type of process measure for care providers from a system usage perspective. The evaluation result was relatively objective and could present a quick understanding of care providers’ work efficiency.

Several potential weaknesses of this study also need to be acknowledged. First, although the ontology-based implementation of the pathway can represent the knowledge in a shareable and elegant way, the adjustment and expansion of ontology need to be completed by knowledge engineers. Care providers encountered some degree of difficulty in understanding the logical rules contained in the ontology. Second, the number of current patients with MCC in our system is relatively small, and we only provide support for one kind of MCC (HTN with T2DM) in the current service engine. The effect of our system on a large scale of patients with diverse MCC requires further exploration. Moreover, several parts of medical knowledge in the guidelines were not integrated in the present management plan, such as proper handling methods for severe acute complications and detailed drug dosage guidance. Third, the long-term compliance remains low in patients. More effective strategies need to be considered to enhance patients’ intrinsic motivation. The long-term work efficiency of care providers also needs to be improved through further medical education. Finally, the evaluation on work efficiency of care providers only considered the regular follow-up task of GPs, and the assessment method did not involve analysis on concrete intervention content.

### Future Work

In future work, we will keep optimizing the usability of the system and support other common chronic conditions such as asthma, stroke, and chronic kidney disease. More elements concerned with health behavior theory (eg, behavior change technologies [71]) could be incorporated into the system to further improve patient compliance. We also plan to deploy our system in more regions of China and perform the evaluation at a larger scale. The evaluation methods will also be refined to provide more comprehensive and credible evidence, such as cost-effectiveness analysis. Another direction for future work is to explore a more personalized care pathway for a specific patient through advanced artificial intelligence technologies such as using reinforcement learning techniques to schedule the follow-up for patients and generate more precise self-management suggestions based on their self-monitoring data.

### Conclusions

This study revealed the commonality in the management of different chronic diseases and explored the feasibility of integrating multiple chronic conditions into a single telehealth system. Management models could be customized for specific policy and challenges in different areas to maximize effectiveness. The tailored closed-loop care pathway proved to be feasible and effective under the “three-manager” mode in China. A part of patient outcomes improved after receiving management through the system, whereas the work efficiency of care providers differed individually. Further research might investigate the effect of such systems in a higher evidence level or introduce state-of-the-art machine learning techniques for a more individualized care pathway.

## Appendix

Multimedia Appendix 1 Detailed description of each disease-specific care pathway.

Multimedia Appendix 2 Detailed information of the Universal Care Pathway Ontology.

Multimedia Appendix 3 Detailed screenshots of the web platform for care providers.

Multimedia Appendix 4 Detailed screenshots of the mobile app for patients.

Multimedia Appendix 5 Subgroup analysis on patients with different diseases and in different age or gender for the comparison tests.

Multimedia Appendix 6 Detailed results of refutation in causal inference.

## Abbreviations

API: application programming interface
ATE: average treatment effect
BG: blood glucose
BP: blood pressure
CCM: chronic care model
CM: case manager
COPD: chronic obstructive pulmonary disease
FBG: fasting blood glucose
GP: general practitioner
HTN: hypertension
MCC: multiple chronic conditions
OWL: W3C Web Ontology Language
PEF: peak expiratory flow
PSM: propensity score matching
PSS: propensity score stratification
SBP: systolic blood pressure
SWRL: semantic web rule language
T2DM: type 2 diabetes mellitus
UCPO: Universal Care Pathway Ontology
