# Supplementary material for: Pathway-Driven Coordinated Telehealth System for Management of Patients With Single or Multiple Chronic Diseases in China: System Development and Retrospective Study
Source: JMIR Med Inform. 2021 May 17;9(5):e27228. doi: 10.2196/27228 (PMC8167615; doi:10.2196/27228)
Supplement: Multimedia Appendix 1 [file medinform_v9i5e27228_app1.docx]

**Detailed description of each disease-specific care pathway**

In this supplementary material, we provided a detailed description of three disease-specific care pathways­—hypertension (HTN), type 2 diabetes mellitus (T2DM), and chronic obstructive pulmonary disease (COPD).

**1 Hypertension**

For HTN, an overview of the designed care pathway is presented in Figure 1.

**Figure 1**. Overview of the HTN care pathway.


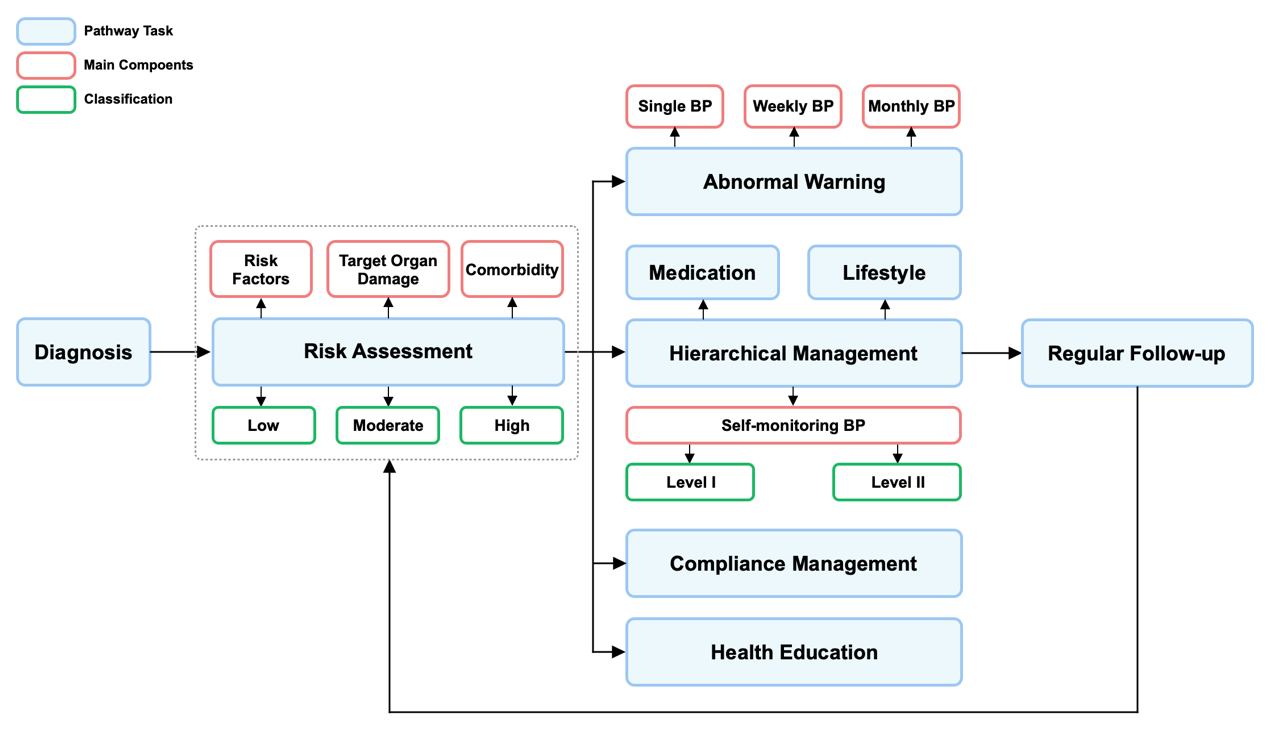


The description of each pathway task is as follow:

**Diagnosis**. Diagnosis of hypertension should be conducted by physicians within the hospital based on the clinical BP measurement. Hypertension is defined as a clinic systolic BP >= 140 mmHg and/or diastolic BP >= 90 mmHg without the use of anti-hypertensive medications at three visits on different days. Table 1 shows the definitions and classification of BP levels.

**Table 1**. Definitions and classifications of BP levels.

| **Category** | | **SBP (mmHg)** | **DBP (mmHg)** |
| --- | --- | --- | --- |
| Normal | | < 120 | < 80 |
| High normal | | 120-139 and (or) | <140/90 |
| Hypertension | | >= 140 and (or) | >= 90 |
|  | Grade 1 (mild) | 140-159 and (or) | 90-99 |
|  | Grade 2 (moderate) | 160-179 and (or) | 100-109 |
|  | Grade 3 (severe) | >= 180 and (or) | >= 110 |
| Isolated systolic hypertension | | >= 140 and | < 90 |

**Risk assessment**. We provide a cardiovascular risk assessment for HTN patients. Patients will be divided into three levels of cardiovascular risk: low risk, moderate risk, and high risk, as shown in Figure 2. We simplify the original assessment provided in the guideline [1] for better practical execution. Generally, the assessment should be conducted at least once per three months.

**Figure 2**. Risk assessment for HTN patients.


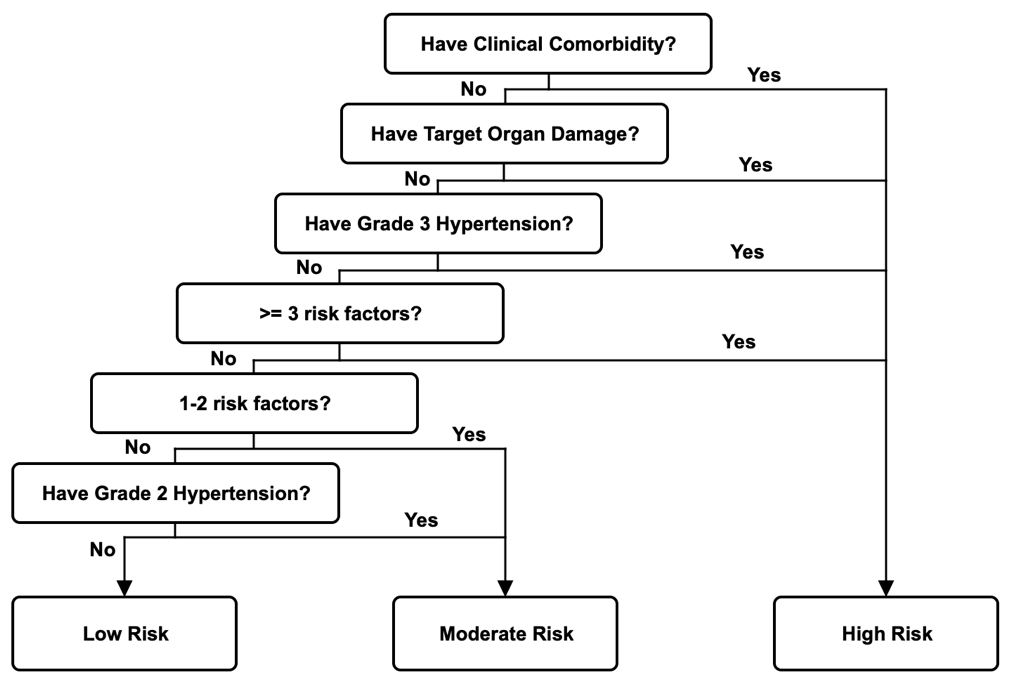


**Hierarchical management**. According to whether patients reach target BP (weekly average BP), we divided them into two levels. The risk level will be used for the initial classification. For HTN patients, the control target of BP is 140/90 mmHg (in T2DM pathway, the control target of BP differs for different risk levels). Figure 3 shows the flow chart of hierarchical management. The recommended self-monitoring frequency of BP for Level I and Level II patients is at least once per day and twice per day (for level II week 3, the frequency can be increased to three times per day), respectively. Body weight and heart rate are also required for self-monitoring.

**Figure 3**. Hierarchical management for HTN patient.


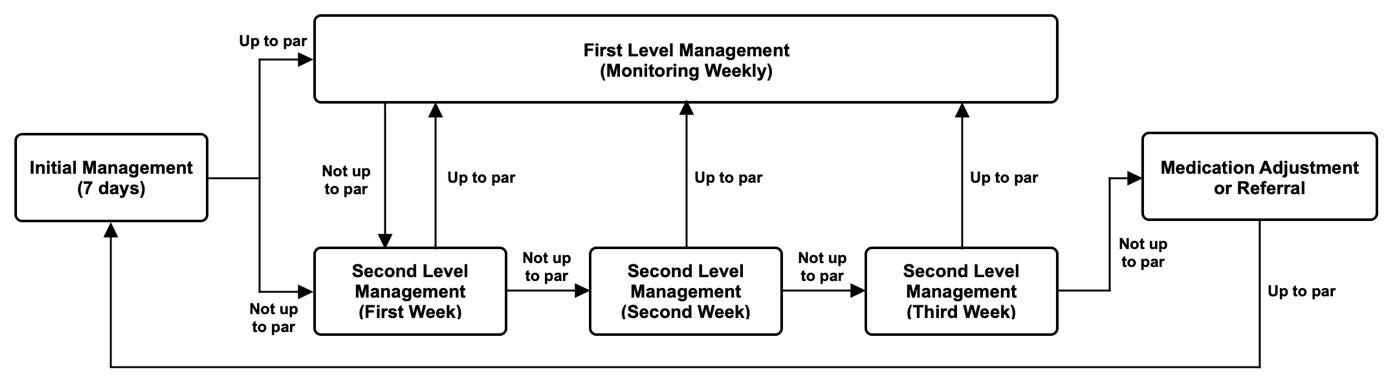


**Regular Follow-up**. For different levels of patients, the regular follow-up frequency also differs and will be adjusted in response to the change of levels. For level I patients, the follow-up frequency is once every three months; for Level II patients, the follow-up frequency is once every two weeks. Figure 4 shows the flow chart of regular follow-up scheduling.

**Figure 4**. Regular follow-up scheduling for HTN patients.


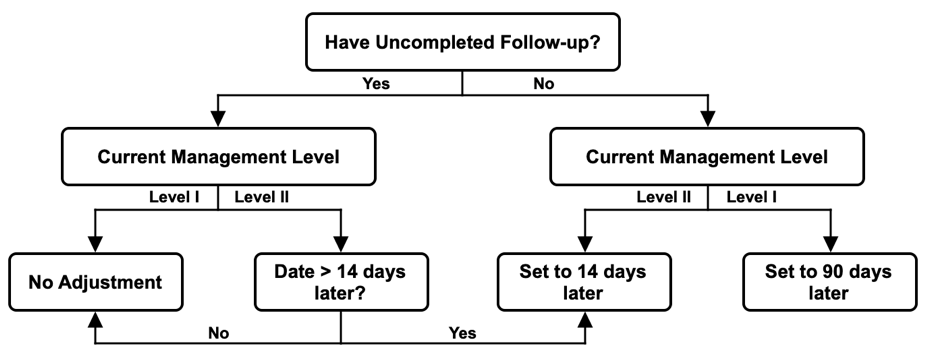


**Medication guidance**. For HTN patients, we provide a personalized medication plan based on the risk assessment results as well as the control of BP. If the patient is in low risk or moderate risk level, we recommend performing lifestyle first for at most three months. When lifestyle change alone is unable to reach the target BP, drug therapy should be initiated. The flow chart of medication guidance is shown in Figure 5.

**Figure 5**. Medication guidance for HTN patients.


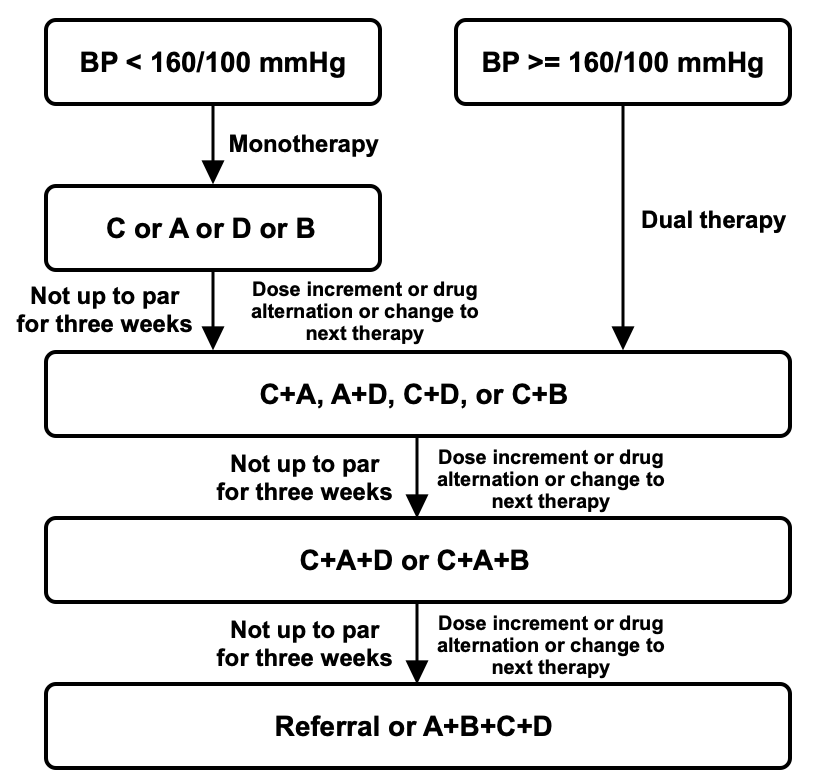


**Lifestyle guidance**. For HTN patients, the lifestyle intervention mainly focuses on diet suggestion (reduce sodium intake, no drinking and smoking), exercise suggestion (medium intensity; 4 to 7 times per week; 30 to 60 min each time), and mentality suggestion (reduce mental stress and maintain psychological balance).

**Abnormal condition intervention**. Table 2 shows the thresholds of abnormal warnings for HTN patients. We mainly focus on three indices: single BP, weekly BP, and monthly BP. The abnormal heart rate and discomfort are also involved. The warning with higher priority will overwrite the warning with lower priority. Care providers should pay attention to the particular patient or conduct the intervention immediately for the warning.

**Table 2**. Abnormal warning for HTN patients.

| **Warning Type** | **Thresholds** | **Intervention** |
| --- | --- | --- |
| High BP (Mild) | 140 mmHg <= SBP <= 160 mmHg or  90 mmHg <= DBP <= 100 mmHg | Close attention |
| High BP (Moderate) | 160 mmHg < SBP <= 180 mmHg or  100 mmHg < DBP <= 110 mmHg | Close attention |
| High BP (Severe) | SBP > 180 mmHg or DBP > 110 mmHg | Immediate contact |
| Low BP | SBP < 90 mmHg or DBP < 60 mmHg | Immediate contact |
| Weekly High BP | SBP > 160 mmHg or DBP > 100 mmHg | Immediate contact |
| Monthly High BP | SBP > 140 mmHg or DBP > 90 mmHg | Immediate contact |
| Abnormal HR | HR > 100 bpm or HR < 50 bpm | Immediate contact |
| Discomfort | Any unexpectable symptoms | Immediate contact |

**Health education**. Educational materials for HTN patients include videos and articles concerned with the general knowledge of HTN, diet suggestion (recipes), physical exercise guidance, and so on. Further, we provide a systematic educational curriculum concerning based on guidelines and books, which is more professional and suitable for patients who want to learn more about their disease.

**Compliance management**. Patient compliance will be calculated every week (actually, the engine calculated it every day based on the records of last 7 days). We define the compliance as the ratio of actual frequency of records, to the prescribed number of records for the blood pressure. The prescribed number is personalized according to the management level (for Level I patients, the prescribed number is 7 times a week; for Level II patients, the prescribed number is unified into 14 times a week). Patient compliance will be divided into 5 levels according to the calculated value. An extra follow-up or reminder will be arranged for low compliance.

$$compliance= min(1, \frac{N\left（ Blood Pressure \right）}{N\left（ prescribed number of records \right）}\times100\%)$$

**2 Diabetes mellitus**

For T2DM, an overview of the designed care pathway is presented in Figure 6.

**Figure 6**. Overview of the T2DM care pathway.


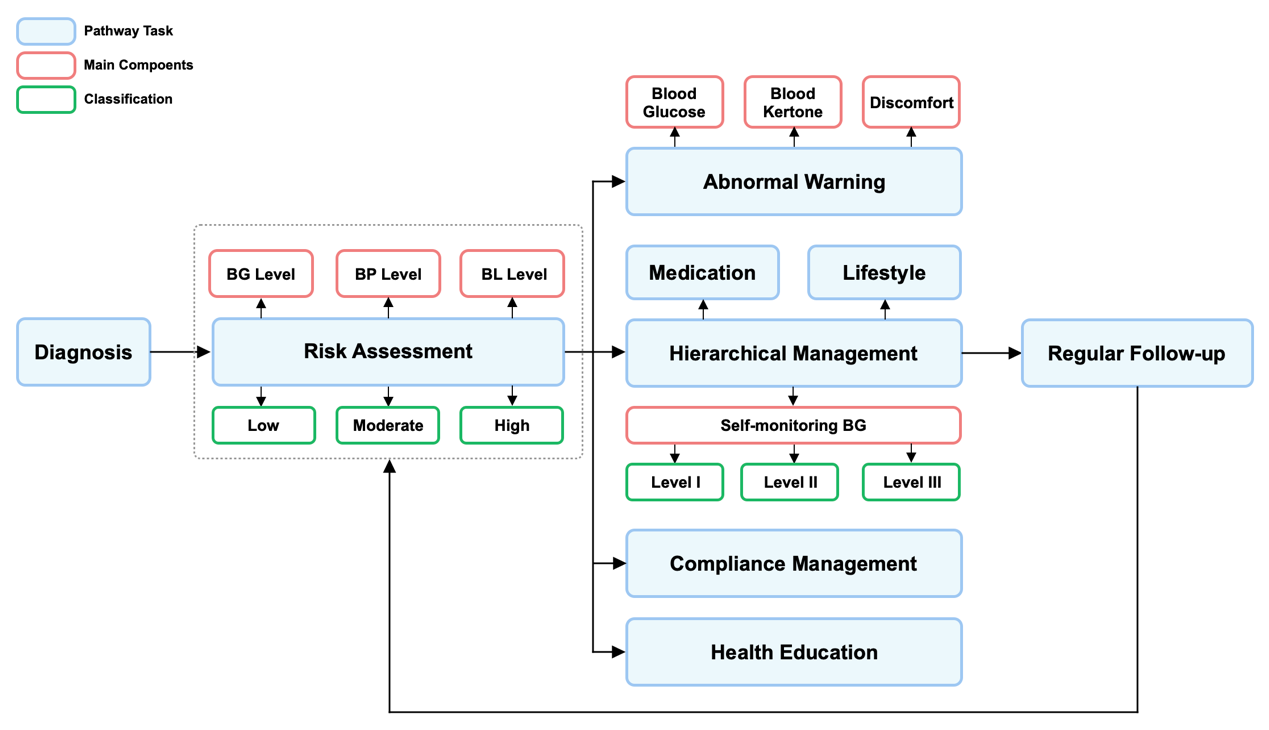


**Diagnosis**. Diagnosis of T2DM should be conducted by physicians within the hospital. Fasting plasma glucose (FPG), random plasma glucose, oral glucose tolerance test (OGTT) 2-hour plasma glucose (2hPG) can be used to diagnose diabetes. The diagnostic criteria are: (a) typical symptoms of diabetes plus random plasma glucose >= 11.1 mmol/L; (b) FPG >= 7.0 mmol/L; (c) OGTT 2hPG >= 11.1 mmol/L [2]. Further, the concrete type of diabetes should be identified based on the typical clinical features.

**Risk assessment**. We provide a comprehensive assessment for T2DM patients involving blood glucose (BG) level, blood pressure (BP) level, and blood lipid (BL) level. The concrete flow chart of each assessment is presented in Figure 7. Generally, the assessments should be conducted at least once per three months. Moreover, for the high and moderate risk of BP level, patients will be suggested to further receive diagnosis of hypertension. Once diagnosed, the patient will also enter the HTN pathway for joint management.

**Figure 7**. Risk assessment for T2DM patients.


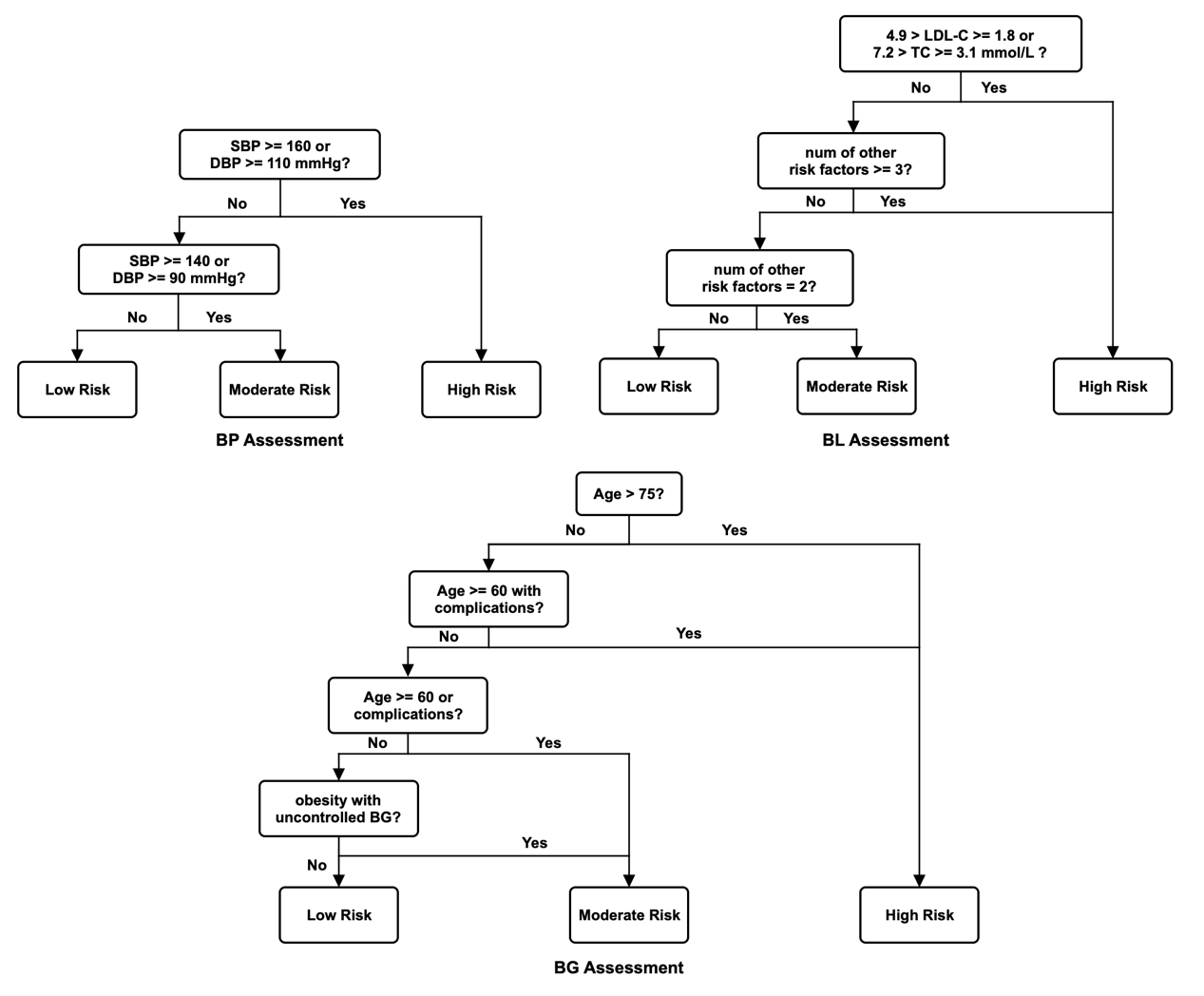


Based on the evaluation results, we provide a personalized control target for each risk level, which is summarized in Table 3.

**Table 3**. Control targets for T2DM patients.

| **Evaluation Level** | **Capillary BG**  **(mmol/L)** | **HbA1c**  **(%)** | **BP**  **(mmHg)** | **LDL-C**  **(mmol/L)** |
| --- | --- | --- | --- | --- |
| High | FBG: 7.8-10  PBG: 7.8-13.9 | 7.0-8.0 | <150/90 | <3.4 |
| Moderate | FBG: 6.1-7.8  PBG: 7.8-10 | 6.5-7.0 | <140/90 | <2.6 |
| Low | FBG: 4.4-6.1  PBG: 6.1-7.8 | <6.5 | <120/80 | <1.8 |

**Hierarchical management**. According to whether patients reach target BG, we divided them into three levels. For different level of patients, the re-classification frequency and the regular follow-up arrangement also differs, as shown in Figure 8.

**Figure 8**. Hierarchical management for T2DM patients.


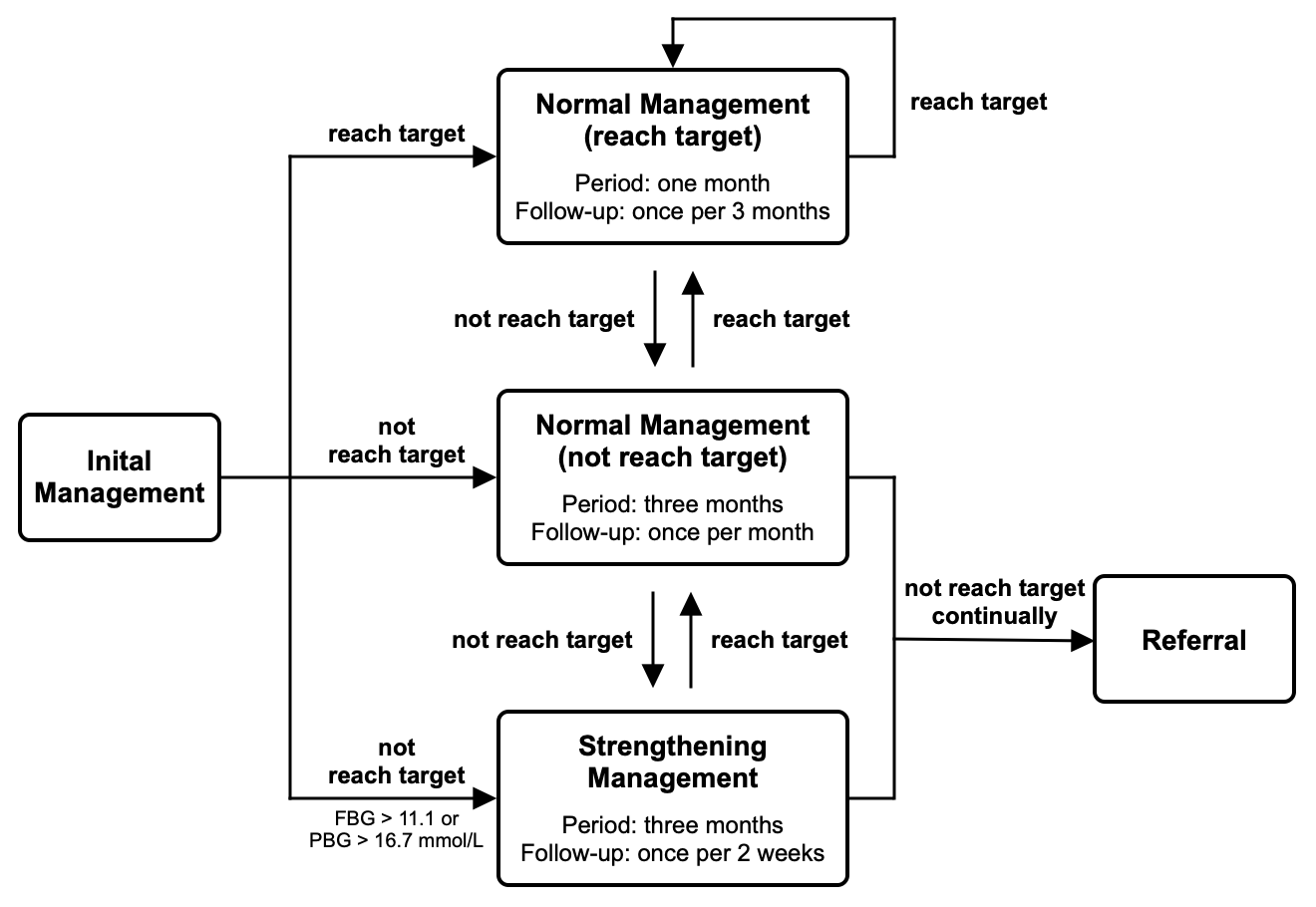


Furthermore, the recommended self-monitoring frequency is also personalized according to the management level as well as the medication plan, as shown in Table 4. Body weight is also recommended to be self-monitored (not presented in the table).

**Table 4**. Self-monitoring plan for T2DM patients (for BG and BP).

| **Management Level**  **Medication**  **Plan** | **Level I** | **Level II** | **Level III** |
| --- | --- | --- | --- |
| Lifestyle intervention or  Monotherapy | BG: three times a week  BP: once a day | BG: six times a week  BP: once a day |  |
| Dual therapy | BG: once a day  BP: once a day | BG: six times a week  BP: once a day |  |
| Triple therapy | BG: nine times a week  BP: once a day | BG: nine times a week  BP: twice a day |  |
| Multiple daily insulin injections | BG: 1-2 times a day  BP: once a day | BG: 2-4 times a day  BP: twice a day | BG: 4-7 times a day  BP: twice a day |

**Regular Follow-up**. As mentioned before, the follow-up frequency differs for different management levels. Further, based on the change of levels, the follow-up schedule will also be dynamically adjusted.

**Medication guidance**. We provide an individualized medication plan for T2DM patients. The plan will be adjusted according to whether patients reached target BG. Figure 9 show the flow chart of medication guidance (along with life intervention). Moreover, the initial mediation plan will be determined based on the Hb1Ac level of patients.

**Figure 9**. Treatment guidance for T2DM patients.


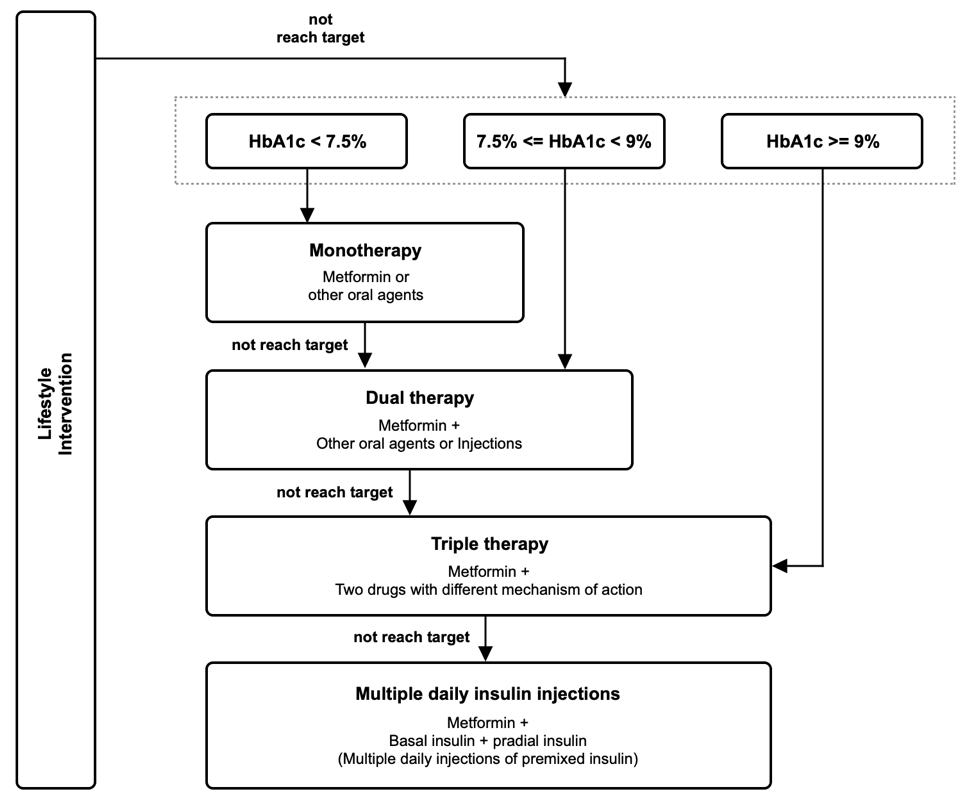


**Lifestyle guidance**. Lifestyle intervention is the basis for T2DM treatment and should be applied throughout the diabetes treatment process. When lifestyle change alone is unable to reach BG target, monotherapy should be initiated (as shown in Figure 9). We provide a suggestion of diet and exercise for T2DM patients, focusing on the daily caloric intake (diet suggestion) and the daily caloric consumption (exercise suggestion).

**Abnormal condition intervention**. Table 5 shows the thresholds of abnormal warnings for T2DM patients. We mainly focus on three indices: blood glucose, blood ketone, and the discomfort. Care providers should pay attention to the particular patient or conduct the intervention immediately for the warning.

**Table 5**. Abnormal warning for T2DM patients.

| **Warning Type** | **Thresholds** | **Intervention** |
| --- | --- | --- |
| Hypoglycemia | BG <= 3.9 mmol/L | Immediate contact |
| Low BG | 3.9 < BG <= 4.4 mmol/L | Close attention |
| High BG | BG > 16.7 mmol/L, Ketone < 0.6 mmol/L | Close attention |
| Ketosis warning | BG > 16.7 mmol/L, Ketone >= 0.6 mmol/L | Immediate contact |
| Extremely high BG | BG > 22.2 mmol/L | Immediate contact |
| Discomfort | polyuria, polydipsia, polyphagia, and any other unexpectable symptoms | Immediate contact |

**Health education**. Educational materials for T2DM patients include videos and articles concerned with the general knowledge of T2DM, diet suggestion (recipes), physical exercise guidance, the damage of complication, and so on.

**Compliance management**. Patient compliance will be calculated every week (same as HTN patients). We define the compliance as the ratio of actual frequency of records, to the prescribed number of records for the blood glucose. The prescribed number is personalized according to the management level and the medication (see Table 4). An extra follow-up or reminder will be arranged for low compliance.

$$compliance= min(1, \frac{N\left（ \mathrm{Blood}\mathrm{Glucose} \right）}{N\left（ prescribed number of records \right）}\times100\%)$$

**3 Chronic obstructive pulmonary disease**

For COPD, an overview of the designed care pathway is presented in Figure 10.

**Figure 10**. Overview of the COPD care pathway.


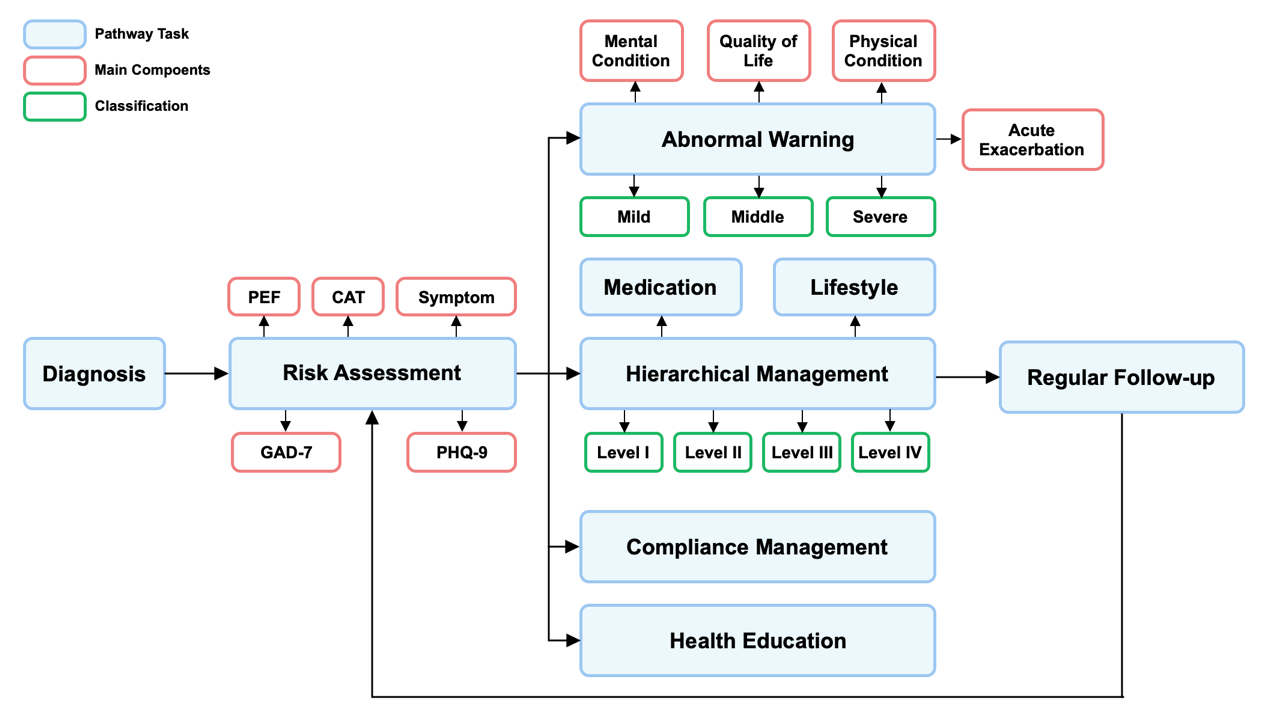


The description of each pathway task is as follow:

**Diagnosis**. Diagnosis of COPD should be conducted by physicians within the hospital. Any patient who has dyspnea, chronic cough or sputum production, and/or a history of exposure to risk factors should be considered for diagnosis. Spirometry is required to make the diagnosis in the hospital. The presence of a post-bronchodilator FEV1/FVC < 0.70 confirms the COPD in patients [3].

**Risk assessment**. We provide a comprehensive assessment of patients conditions with exacerbation risk (symptoms), quality of life (COPD Assessment Test, CAT [4]), mental health (Patient Health Questionnaire-9 scale, PHQ-9 [5], and Generalized Anxiety Disorder 7 scale, GAD-7 [6]) and pulmonary function (peak expiratory flow, PEF) every two weeks and patients were classified into 4 levels, as shown in Table 6. Patients who satisfy all the five conditions are evaluated as level Ⅰ. If any one of five condition meets the threshold of level Ⅱ, Ⅲ or Ⅳ, patient will be classified into the particular level. It should be noted that in the COPD pathway there is no extra patient classification. The frequency of risk assessment for COPD patients is once every two weeks. Patients are also required to submit each type of records at least one time every two weeks.

**Table 6**. Risk assessment of COPD patients.

| **Evaluation Level** | **Symptoms** | **CAT** | **PEF%pred** | **PHQ-9** | **GAD-7** |
| --- | --- | --- | --- | --- | --- |
| I | None | ≤ 10 | ≥ 80% | ≤ 4 | ≤ 4 |
| II | Wheeze, cough, nasal discharge, sore throat | 11-20 | 60-80% | 5-9 | 5-9 |
| III | Sputum purulence or increased, dyspnea | 21-30 | 60-80% | 10-14 | 10-14 |
| IV | Fever, drowsiness | >30 | < 60% | 15-27 | 15-21 |

**Hierarchical management**. According to the risk assessment results, patients will be classified into different levels and receive appropriate management plans accordingly. The management plans mainly consisted of medication guidance and lifestyle guidance, along with intervention from care providers. The details of each part are described in the following paragraphs.

**Medication guidance**. Patients in different levels will receive a tailored two-stage medication plan as shown in Figure 11. The medication plans are formulated according to the guidelines [3] and consent from physicians or GPs is necessary for final prescription. Hospital admission is recommended if patients remain unstable after switching to the second medication plan. Currently, the medication guidance module of COPD patients has not been incorporated into the engine (only coded in the ontology).

**Figure 11**. Medication guidance for COPD patients.


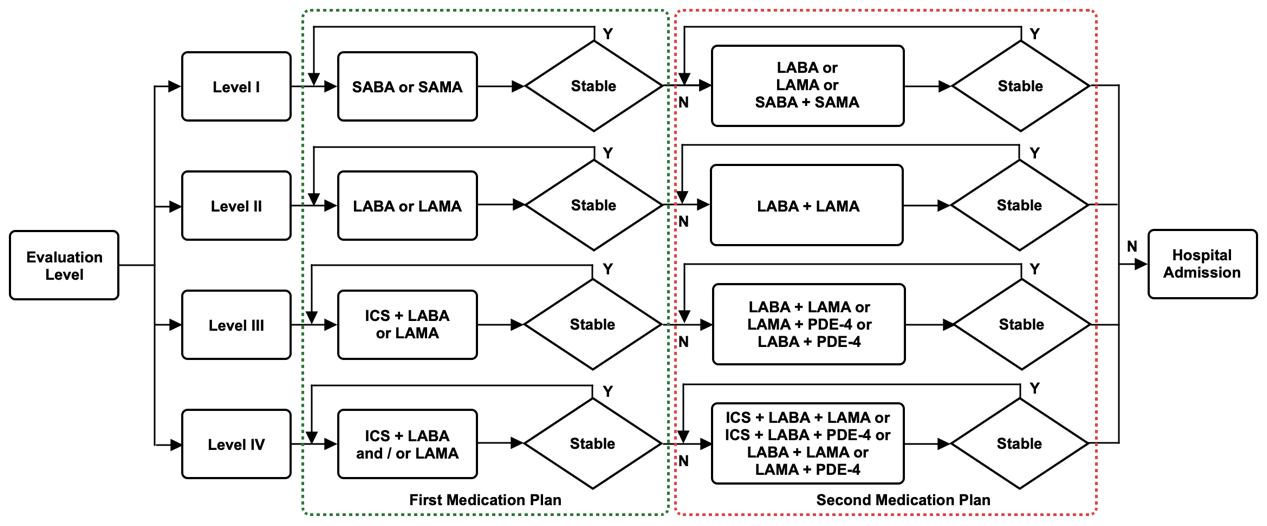


**Lifestyle guidance**. Patients are asked to perform proper pulmonary rehabilitation based on the evaluation level, as shown in Table 7. Videos are provided on the app for each kind of rehabilitation exercise by CMs, demonstrating the details step by step. Moreover, COPD patients are suggested to quit smoking in daily life.

**Table 7**. Pulmonary rehabilitation based on the evaluation level.

| **Evaluation Level** | | I | II | III | IV |
| --- | --- | --- | --- | --- | --- |
| **Limb exercise** | | + | + | + | + |
| **Pursed lips breathing** | | + | + | + | + |
| **Abdominal breathing** | | + | + | + | + |
| **Breathing exercise** | **Seated** | + | + | + | - |
|  | **Stood** | + | + | - | - |
|  | **Laying** | + | + | + | - |
| **Oxygen therapy** | | If patients have indications for oxygen therapy | | | |

**Regular Follow-up**. For each level of patients, regular follow-up is conducted every two weeks. GPs can conduct the follow-up using telephone (the traditional tool) or WeChat—the most popular instant messaging app in China.

**Abnormal condition intervention**. Table 8 shows the thresholds of abnormal condition defined in the pathway. The standard of CAT, PHQ-9, and GAD-7 warnings are defined according to the original paper or guideline of the three scales. The two thresholds of PEF warning—60% and 80% of the standard value—are proposed in our previous study [7]. Moreover, we provide an automatic screening of acute exacerbations based on self-reported discomfort symptoms. Concretely, according to a previously validated definition [8], we considered dyspnea, sputum purulence, and sputum volume as major symptoms, each scored as 5 points, and nasal discharge or congestion, sore throat, cough, and wheeze as minor symptoms, each scored as 1 point. A suspected exacerbation was automatically detected by the system if the summed symptom score was >6 points for ≥ 2 consecutive days. In case of a warning event, an extra follow-up will be arranged.

**Table 8**. Abnormal condition definition for COPD patients.

| **Warning** | **PEF%pred** | **CAT** | **PHQ-9** | **GAD-7** |
| --- | --- | --- | --- | --- |
| Mild | 60-80% | 11-20 | 10-14 | 5-9 |
| Middle | 60-80% | 21-30 | 15-19 | 10-14 |
| Severe | <60% | 31-40 | 20-27 | 15-21 |

**Health education**. Educational materials for COPD patients include videos and articles concerned with the general knowledge of COPD, the damage of smoking, the operation method of peak flow meter, and so on.

**Compliance management**. Patient compliance will be calculated every month. We define the compliance as the ratio of actual frequency of records, to the prescribed number of records for the PEF, CAT scale, PHQ-9 scale, and GAD-7 scale. Patients were required to submit these 4 types of data every 2 weeks. An extra follow-up or reminder will be arranged for low compliance.

$$compliance= min(1, \frac{N\left（ \mathrm{PEF} \right）+N\left（ \mathrm{CAT} \right）+N\left（ \mathrm{PHQ} \right）+N\left（ \mathrm{GAD} \right）}{N\left（ prescribed number of records \right）}\times100\%)$$

**Reference**

1. Joint Committee for Guideline Revision. 2018 Chinese Guidelines for Prevention and Treatment of Hypertension-A report of the Revision Committee of Chinese Guidelines for Prevention and Treatment of Hypertension. *J Geriatr Cardiol*. 2019;16(3):182-241. PMID:31080465

2. World Health Organization. *Definition, Diagnosis and Classification of Diabetes Mellitus and Its Complications : Report of a WHO Consultation. Part 1, Diagnosis and Classification of Diabetes Mellitus*.; 1999. https://apps.who.int/iris/handle/10665/66040.

3. Disease 2020 Global Initiative for Chronic Obstructive Lung. *Global Initiative for Chronic Obstructive Lung Disease (GOLD). Global Strategy for the Diagnosis, Management and Prevention of COPD, (2020 Report)*.; 2020.

4. Jones PW, Harding G, Berry P, Wiklund I, Chen W-H, Kline Leidy N. Development and first validation of the COPD Assessment Test. *Eur Respir J*. 2009;34(3):648-654. PMID:19720809

5. Kroenke K, Spitzer RL, Williams JB. The PHQ-9: validity of a brief depression severity measure. *J Gen Intern Med*. 2001;16(9):606-613. PMID:11556941

6. Spitzer RL, Kroenke K, Williams JBW, Löwe B. A brief measure for assessing generalized anxiety disorder: the GAD-7. *Arch Intern Med*. 2006;166(10):1092-1097. PMID:16717171

7. Deng N, Chen J, Liu Y, et al. Using Mobile Health Technology to Deliver a Community-Based Closed-Loop Management System for Chronic Obstructive Pulmonary Disease Patients in Remote Areas of China: Development and Prospective Observational Study. *JMIR mHealth uHealth*. 2020;8(11):e15978. PMID:33237036

8. Aaron SD, Donaldson GC, Whitmore GA, Hurst JR, Ramsay T, Wedzicha JA. Time course and pattern of COPD exacerbation onset. *Thorax*. 2012;67(3):238-243. PMID:22008189
