# Supplementary material for: Pathway-Driven Coordinated Telehealth System for Management of Patients With Single or Multiple Chronic Diseases in China: System Development and Retrospective Study
Source: JMIR Med Inform. 2021 May 17;9(5):e27228. doi: 10.2196/27228 (PMC8167615; doi:10.2196/27228)
Supplement: Multimedia Appendix 2 [file medinform_v9i5e27228_app2.docx]

**Detailed information of the Universal Care Pathway Ontology**

In this supplementary material, we provide detailed information of the constructed ontology (UCPO), including the ontology metrics and the description of SWRL rule sets for disease-specific pathway. UCPO was implemented based on an OWL 2 profile called OWL RL, which enables the implementation of polynomial time reasoning algorithms using rule-extended database technologies operating directly on RDF triples.

**Ontology metrics**

The metrics of UCPO derived by protégé are presented in Table 1. Screenshots of the constructed class hierarchy, properties, and individuals are presented in Figure 1.

**Table 1**. UCPO ontology metrics.

| Metric | | Count |
| --- | --- | --- |
| **Overall Metrics** | |  |
|  | Classes | 97 |
|  | Axioms | 1356 |
|  | Logical Axioms | 911 |
|  | Declaration Axioms | 333 |
|  | Object Properties | 16 |
|  | Data Properties | 64 |
|  | Individuals | 155 |
|  | SWRL Rules | 307 |
| **Class Axioms** | |  |
|  | SubClassOf Axioms | 103 |
|  | DisjointClasses Axioms | 10 |
| **Object Property Axioms** | |  |
|  | ObjectPropertyDomain Axioms | 3 |
|  | ObjectPropertyRange Axioms | 15 |
| **Data Property Axioms** | |  |
|  | DataPropertyDomain Axioms | 20 |
|  | DataPropertyRange Axioms | 64 |
| **Individual Axioms** | |  |
|  | ClassAssertion Axioms | 155 |
|  | ObjectPropertyAssertion Axioms | 147 |
|  | DataPropertyAssertion Axioms | 83 |

**Figure 1**. Screenshots of the constructed class hierarchy, properties, and indivifuals.


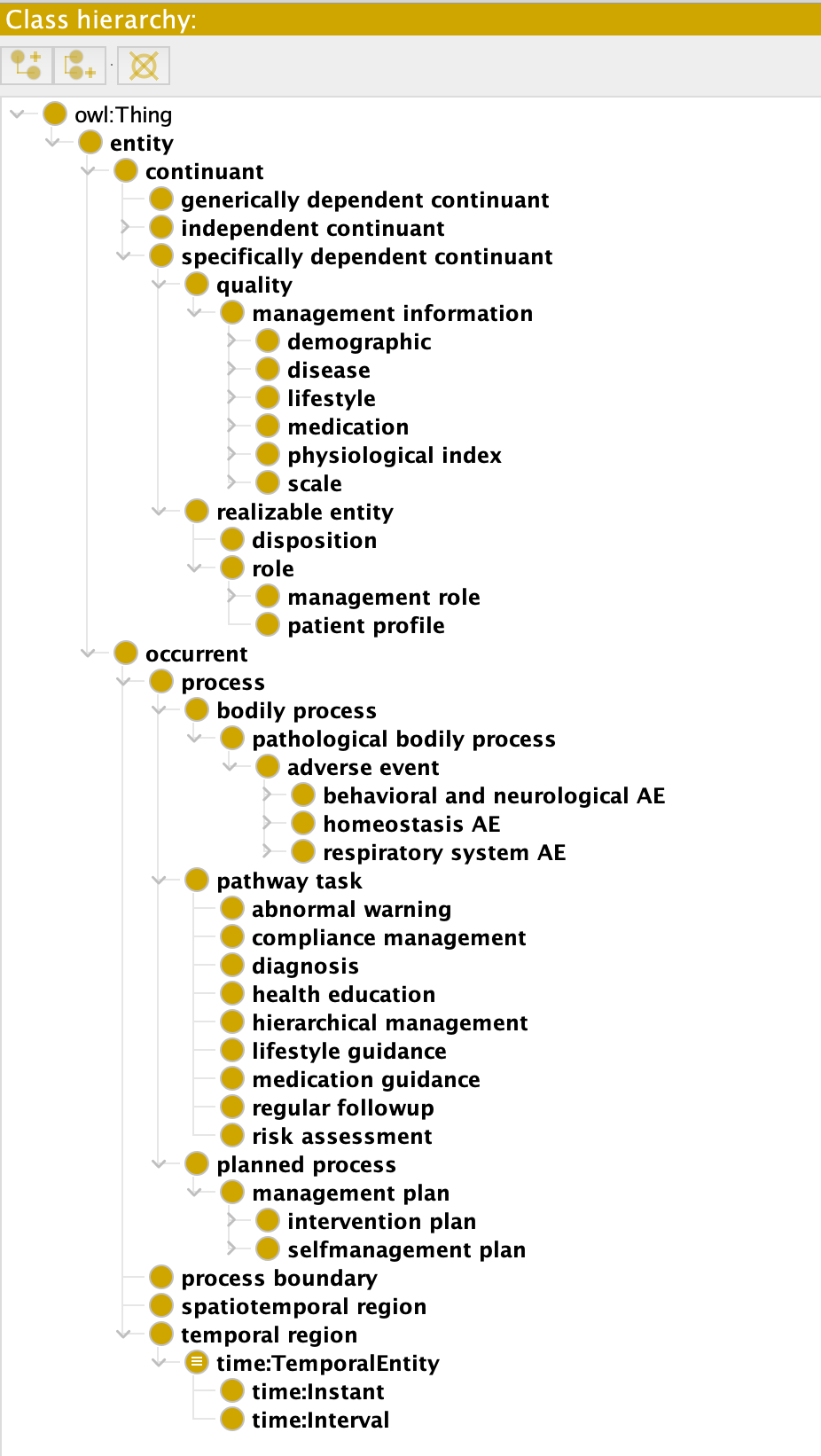

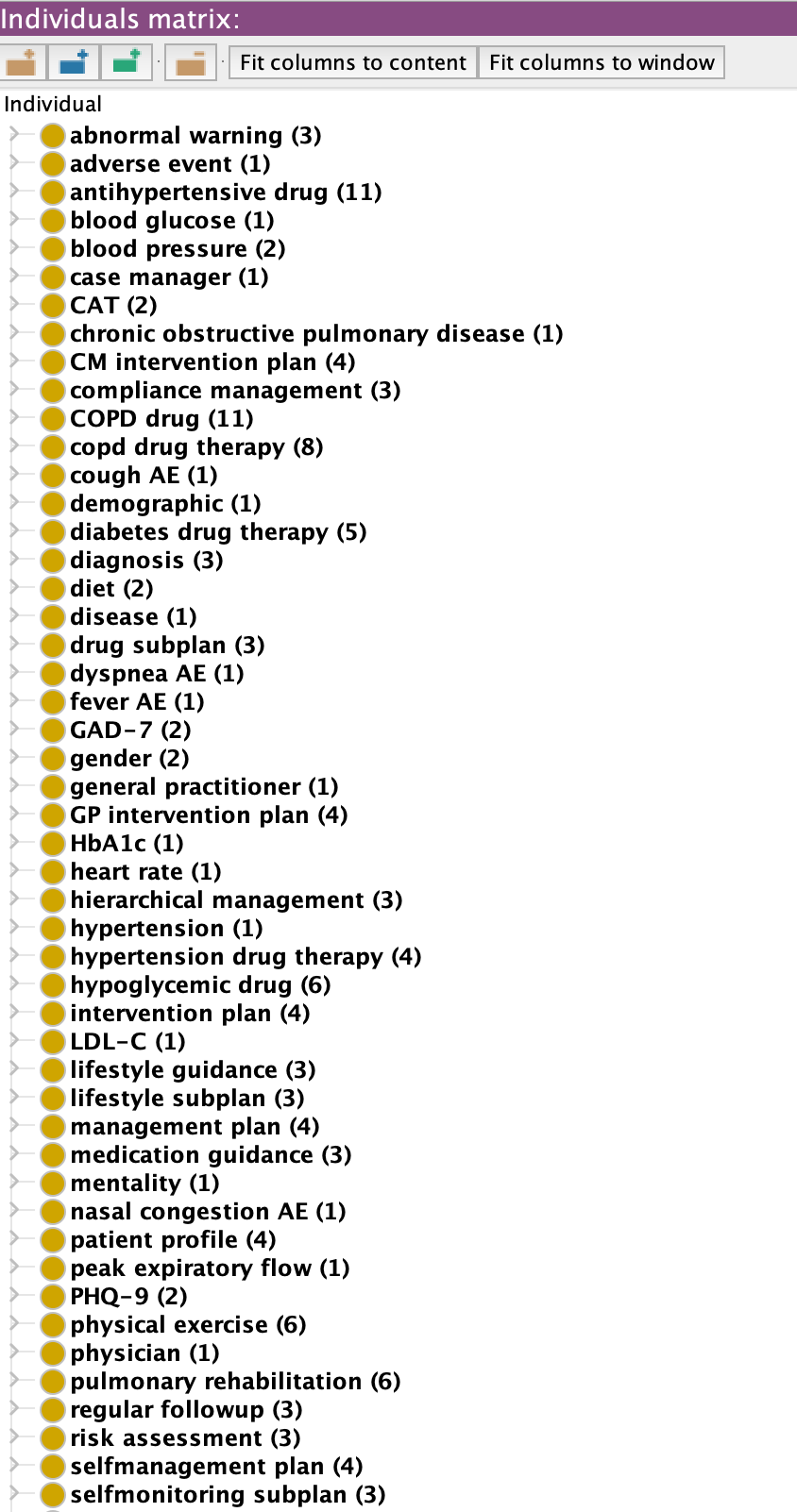


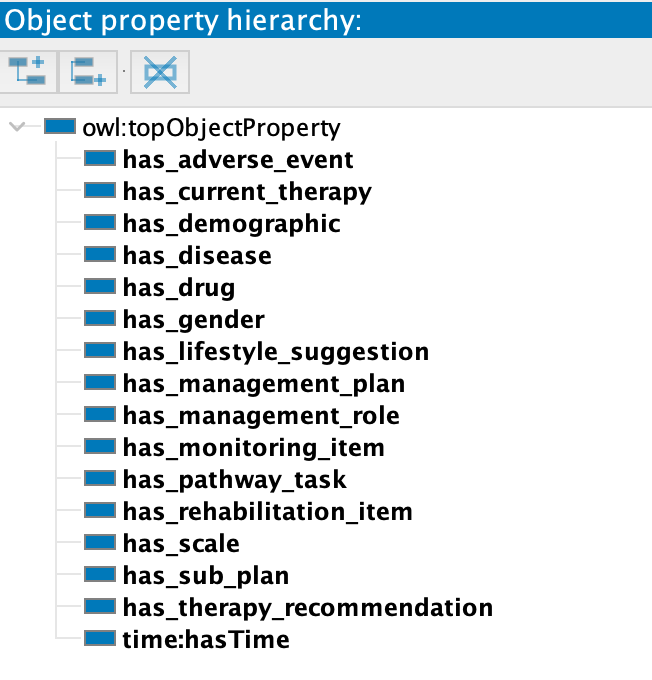

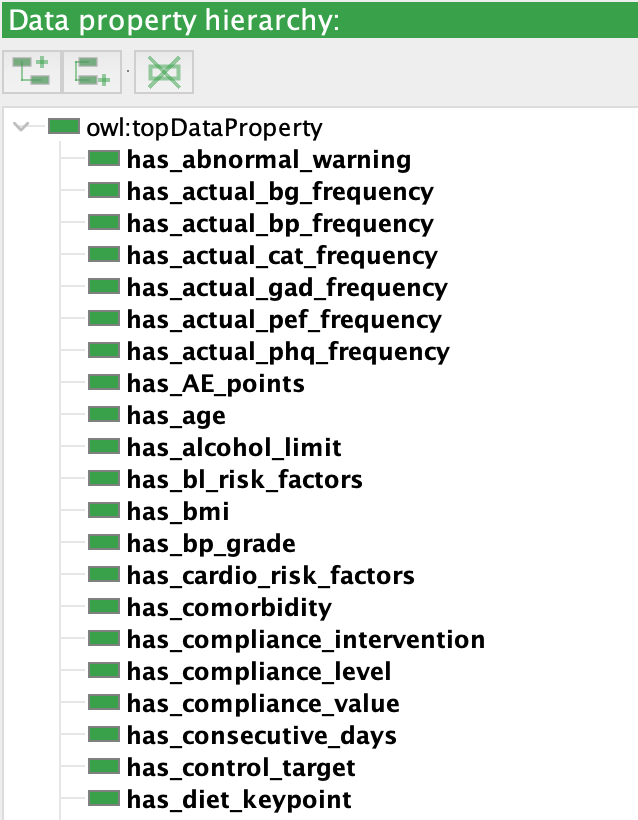


**SWRL rule set description**

The rule set of a single disease pathway can be divided into 2 parts: pathway task rule and management plan rule. Table 2 presents the overall metrics of the constructed SWRL rules for the three diseases. For multiple chronic conditions (MCC), an extra rule set will be defined to merge the management plans generated for different single diseases. Moreover, the system will automatically deal with the potential redundancies and conflicts of properties in the merged management plan. One other thing to note is that SWRL rules only support monotonic inference, therefore the control layer of the engine will perform external non-monotonic inference to implement the complete decision support. We will present the SWRL rule set using the HTN pathway as an example (see Table 3 and Table 4).

**Table 2**. SWRL rule metrics.

| Metric | | | Count (rules) |
| --- | --- | --- | --- |
| **Hypertension** | | | 63 |
|  | **Pathway Task** | | 52 |
|  |  | Diagnosis | 1 |
|  |  | Risk Assessment | 7 |
|  |  | Hierarchical Management | 7 |
|  |  | Regular Follow-up | 5 |
|  |  | Medication Guidance | 6 |
|  |  | Lifestyle Guidance | 1 |
|  |  | Abnormal Warning | 15 |
|  |  | Compliance Management | 10 |
|  | Management Plan | | 11 |
| **Type 2 Diabetes Mellitus** | | | 174 |
|  | **Pathway Task** | | 133 |
|  |  | Diagnosis | 1 |
|  |  | Risk Assessment | 17 |
|  |  | Hierarchical Management | 25 |
|  |  | Regular Follow-up | 8 |
|  |  | Medication Guidance | 6 |
|  |  | Lifestyle Guidance | 15 |
|  |  | Abnormal Warning | 6 |
|  |  | Compliance Management | 55 |
|  | Management Plan | | 41 |
| **Chronic Obstructive Pulmonary Disease** | | | 65 |
|  | **Pathway Task** | | 55 |
|  |  | Diagnosis | 1 |
|  |  | Risk Assessment | 22 |
|  |  | Hierarchical Management | 0 |
|  |  | Regular Follow-up | 2 |
|  |  | Medication Guidance | 8 |
|  |  | Lifestyle Guidance | 4 |
|  |  | Abnormal Warning | 13 |
|  |  | Compliance Management | 5 |
|  | Management Plan | | 10 |
| **Hypertension with Type 2 Diabetes Mellitus** | | | 5 |
|  | Management Plan (Merge) | | 5 |
| Total | | | 307 |

**Table 3**. Pathway task rule for hypertension.

| Category | Rule Number | SWRL Rule |
| --- | --- | --- |
| **Diagnosis** | **HTN-diagnosis-01** | patient_profile(?p) ^ has_disease(?p, Hypertension) ^ has_pathway_task(?p, ?d) ^ diagnosis(?d) -> is_diagnosed_hypertension(?d, true) |
| **Risk Assessment** | **HTN-risk_assessment-01** | patient_profile(?p) ^ has_disease(?p, Hypertension) ^ has_pathway_task(?p, ?r) ^ risk_assessment(?r) ^ has_comorbidity(?r, true) -> has_risk_level(?r, "high risk") |
|  | **HTN-risk_assessment-02** | patient_profile(?p) ^ has_disease(?p, Hypertension) ^ has_pathway_task(?p, ?r) ^ risk_assessment(?r) ^ has_comorbidity(?r, false) ^ has_target_organ_damage(?r, true) -> has_risk_level(?r, "high risk") |
|  | **HTN-risk_assessment-03** | patient_profile(?p) ^ has_disease(?p, Hypertension) ^ has_pathway_task(?p, ?r) ^ risk_assessment(?r) ^ has_comorbidity(?r, false) ^ has_target_organ_damage(?r, false) ^ has_bp_grade(?r, 3) -> has_risk_level(?r, "high risk") |
|  | **HTN-risk_assessment-04** | patient_profile(?p) ^ has_disease(?p, Hypertension) ^ has_pathway_task(?p, ?r) ^ risk_assessment(?r) ^ has_comorbidity(?r, false) ^ has_target_organ_damage(?r, false) ^ has_bp_grade(?r, ?v) ^ swrlb:notEqual(?v, 3) ^ has_cardio_risk_factors(?r, ?c) ^ swrlb:greaterThanOrEqual(?c, 3) -> has_risk_level(?r, "high risk") |
|  | **HTN-risk_assessment-05** | patient_profile(?p) ^ has_disease(?p, Hypertension) ^ has_pathway_task(?p, ?r) ^ risk_assessment(?r) ^ has_comorbidity(?r, false) ^ has_target_organ_damage(?r, false) ^ has_bp_grade(?r, ?v) ^ swrlb:notEqual(?v, 3) ^ has_cardio_risk_factors(?r, ?c) ^ swrlb:lessThan(?c, 3) ^ swrlb:greaterThanOrEqual(?c, 1) -> has_risk_level(?r, "moderate risk") |
|  | **HTN-risk_assessment-06** | patient_profile(?p) ^ has_disease(?p, Hypertension) ^ has_pathway_task(?p, ?r) ^ risk_assessment(?r) ^ has_comorbidity(?r, false) ^ has_target_organ_damage(?r, false) ^ has_bp_grade(?r, 2) ^ has_cardio_risk_factors(?r, ?c) ^ swrlb:lessThan(?c, 1) -> has_risk_level(?r, "moderate risk") |
|  | **HTN-risk_assessment-07** | patient_profile(?p) ^ has_disease(?p, Hypertension) ^ has_pathway_task(?p, ?r) ^ risk_assessment(?r) ^ has_comorbidity(?r, false) ^ has_target_organ_damage(?r, false) ^ has_bp_grade(?r, 1) ^ has_cardio_risk_factors(?r, ?c) ^ swrlb:lessThan(?c, 1) -> has_risk_level(?r, "low risk") |
| **Hierarchical Management** | **HTN-hierarchical_management-01** | patient_profile(?p) ^ has_disease(?p, Hypertension) ^ has_pathway_task(?p, ?h) ^ hierarchical_management(?h) ^ is_initial_classified(?h, true) ^ has_risk_level(?h, "high risk") -> has_management_level(?h, 2) |
|  | **HTN-hierarchical_management-02** | patient_profile(?p) ^ has_disease(?p, Hypertension) ^ has_pathway_task(?p, ?h) ^ hierarchical_management(?h) ^ is_initial_classified(?h, true) ^ has_risk_level(?h, ?r) ^ swrlb:notEqual(?r, "high risk") ^ has_weekly_sbp(?h, ?sbp) ^ swrlb:lessThan(?sbp, 140) ^ has_weekly_dbp(?h, ?dbp) ^ swrlb:lessThan(?dbp, 90) -> has_management_level(?h, 1) |
|  | **HTN-hierarchical_management-03** | patient_profile(?p) ^ has_disease(?p, Hypertension) ^ has_pathway_task(?p, ?h) ^ hierarchical_management(?h) ^ is_initial_classified(?h, true) ^ has_risk_level(?h, ?r) ^ swrlb:notEqual(?r, "high risk") ^ has_weekly_sbp(?h, ?sbp) ^ swrlb:greaterThanOrEqual(?sbp, 140) -> has_management_level(?h, 2) |
|  | **HTN-hierarchical_management-04** | patient_profile(?p) ^ has_disease(?p, Hypertension) ^ has_pathway_task(?p, ?h) ^ hierarchical_management(?h) ^ is_initial_classified(?h, true) ^ has_risk_level(?h, ?r) ^ swrlb:notEqual(?r, "high risk") ^ has_weekly_dbp(?h, ?dbp) ^ swrlb:greaterThanOrEqual(?dbp, 90) -> has_management_level(?h, 2) |
|  | **HTN-hierarchical_management-05** | patient_profile(?p) ^ has_disease(?p, Hypertension) ^ has_pathway_task(?p, ?h) ^ hierarchical_management(?h) ^ is_initial_classified(?h, false) ^ has_weekly_sbp(?h, ?sbp) ^ swrlb:lessThan(?sbp, 140) ^ has_weekly_dbp(?h, ?dbp) ^ swrlb:lessThan(?dbp, 90) -> has_management_level(?h, 1) |
|  | **HTN-hierarchical_management-06** | patient_profile(?p) ^ has_disease(?p, Hypertension) ^ has_pathway_task(?p, ?h) ^ hierarchical_management(?h) ^ is_initial_classified(?h, false) ^ has_weekly_sbp(?h, ?sbp) ^ swrlb:greaterThanOrEqual(?sbp, 140) -> has_management_level(?h, 2) |
|  | **HTN-hierarchical_management-07** | patient_profile(?p) ^ has_disease(?p, Hypertension) ^ has_pathway_task(?p, ?h) ^ hierarchical_management(?h) ^ is_initial_classified(?h, false) ^ has_weekly_dbp(?h, ?dbp) ^ swrlb:greaterThanOrEqual(?dbp, 90) -> has_management_level(?h, 2) |
| **Regular Followup** | **HTN-regular_followup-01** | patient_profile(?p) ^ has_disease(?p, Hypertension) ^ has_pathway_task(?p, ?f) ^ regular_followup(?f) ^ has_uncompleted_followup(?f, true) ^ time:hasTime(?f, ?t) ^ has_management_level(?f, 1) -> has_new_followup_interval(?t, -1) |
|  | **HTN-regular_followup-02** | patient_profile(?p) ^ has_disease(?p, Hypertension) ^ has_pathway_task(?p, ?f) ^ regular_followup(?f) ^ has_uncompleted_followup(?f, true) ^ time:hasTime(?f, ?t) ^ has_management_level(?f, 2) ^ has_followup_interval(?t, ?i) ^ swrlb:greaterThan(?i, 14) -> has_new_followup_interval(?t, 14) |
|  | **HTN-regular_followup-03** | patient_profile(?p) ^ has_disease(?p, Hypertension) ^ has_pathway_task(?p, ?f) ^ regular_followup(?f) ^ has_uncompleted_followup(?f, true) ^ time:hasTime(?f, ?t) ^ has_management_level(?f, 2) ^ has_followup_interval(?t, ?i) ^ swrlb:lessThanOrEqual(?i, 14) -> has_new_followup_interval(?t, -1) |
|  | **HTN-regular_followup-04** | patient_profile(?p) ^ has_disease(?p, Hypertension) ^ has_pathway_task(?p, ?f) ^ regular_followup(?f) ^ has_uncompleted_followup(?f, false) ^ time:hasTime(?f, ?t) ^ has_management_level(?f, 1) -> has_new_followup_interval(?t, 90) |
|  | **HTN-regular_followup-05** | patient_profile(?p) ^ has_disease(?p, Hypertension) ^ has_pathway_task(?p, ?f) ^ regular_followup(?f) ^ has_uncompleted_followup(?f, false) ^ time:hasTime(?f, ?t) ^ has_management_level(?f, 2) -> has_new_followup_interval(?t, 14) |
| **Medication Guidance** | **HTN-medication_guidance-01** | patient_profile(?p) ^ has_disease(?p, Hypertension) ^ has_pathway_task(?p, ?m) ^ medication_guidance(?m) ^ is_initial_medication(?m, true) ^ has_weekly_sbp(?m, ?sbp) ^ swrlb:lessThan(?sbp, 160) ^ has_weekly_dbp(?m, ?dbp) ^ swrlb:lessThan(?dbp, 110) -> has_therapy_recommendation(?m, HTN_monotherapy) |
|  | **HTN-medication_guidance-02** | patient_profile(?p) ^ has_disease(?p, Hypertension) ^ has_pathway_task(?p, ?m) ^ medication_guidance(?m) ^ is_initial_medication(?m, true) ^ has_weekly_sbp(?m, ?sbp) ^ swrlb:greaterThanOrEqual(?sbp, 160) -> has_therapy_recommendation(?m, HTN_dual_therapy) |
|  | **HTN-medication_guidance-03** | patient_profile(?p) ^ has_disease(?p, Hypertension) ^ has_pathway_task(?p, ?m) ^ medication_guidance(?m) ^ is_initial_medication(?m, true) ^ has_weekly_dbp(?m, ?dbp) ^ swrlb:greaterThanOrEqual(?dbp, 110) -> has_therapy_recommendation(?m, HTN_dual_therapy) |
|  | **HTN-medication_guidance-04** | patient_profile(?p) ^ has_disease(?p, Hypertension) ^ has_pathway_task(?p, ?m) ^ medication_guidance(?m) ^ is_initial_medication(?m, false) ^ is_bp_controlled(?m, false) ^ has_current_therapy(?m, HTN_monotherapy) -> has_therapy_recommendation(?m, HTN_dual_therapy) |
|  | **HTN-medication_guidance-05** | patient_profile(?p) ^ has_disease(?p, Hypertension) ^ has_pathway_task(?p, ?m) ^ medication_guidance(?m) ^ is_initial_medication(?m, false) ^ is_bp_controlled(?m, false) ^ has_current_therapy(?m, HTN_dual_therapy) -> has_therapy_recommendation(?m, HTN_triple_therapy) |
|  | **HTN-medication_guidance-06** | patient_profile(?p) ^ has_disease(?p, Hypertension) ^ has_pathway_task(?p, ?m) ^ medication_guidance(?m) ^ is_initial_medication(?m, false) ^ is_bp_controlled(?m, false) ^ has_current_therapy(?m, HTN_triple_therapy) -> has_therapy_recommendation(?m, HTN_quadruple_therapy) |
| **Lifestyle Guidance** | **HTN-lifestyle_guidance-01** | patient_profile(?p) ^ has_disease(?p, Hypertension) ^ has_pathway_task(?p, ?lg) ^ lifestyle_guidance(?lg) -> has_lifestyle_suggestion(?lg, HTN_diet) ^ has_lifestyle_suggestion(?lg, HTN_exercise) ^ has_lifestyle_suggestion(?lg, HTN_mentality) |
| **Abnormal Warning** | **HTN-abnormal_warning-01** | patient_profile(?p) ^ has_disease(?p, Hypertension) ^ has_pathway_task(?p, ?w) ^ abnormal_warning(?w) ^ has_single_sbp(?w, ?sbp) ^ swrlb:greaterThanOrEqual(?sbp, 140) ^ swrlb:lessThanOrEqual(?sbp, 160) -> has_abnormal_warning(?w, "mild high BP") |
|  | **HTN-abnormal_warning-02** | patient_profile(?p) ^ has_disease(?p, Hypertension) ^ has_pathway_task(?p, ?w) ^ abnormal_warning(?w) ^ has_single_dbp(?w, ?dbp) ^ swrlb:greaterThanOrEqual(?dbp, 90) ^ swrlb:lessThanOrEqual(?dbp, 100) -> has_abnormal_warning(?w, "mild high BP") |
|  | **HTN-abnormal_warning-03** | patient_profile(?p) ^ has_disease(?p, Hypertension) ^ has_pathway_task(?p, ?w) ^ abnormal_warning(?w) ^ has_single_sbp(?w, ?sbp) ^ swrlb:greaterThan(?sbp, 160) ^ swrlb:lessThanOrEqual(?sbp, 180) -> has_abnormal_warning(?w, "moderate high BP") |
|  | **HTN-abnormal_warning-04** | patient_profile(?p) ^ has_disease(?p, Hypertension) ^ has_pathway_task(?p, ?w) ^ abnormal_warning(?w) ^ has_single_dbp(?w, ?dbp) ^ swrlb:greaterThan(?dbp, 100) ^ swrlb:lessThanOrEqual(?dbp, 110) -> has_abnormal_warning(?w, "moderate high BP") |
|  | **HTN-abnormal_warning-05** | patient_profile(?p) ^ has_disease(?p, Hypertension) ^ has_pathway_task(?p, ?w) ^ abnormal_warning(?w) ^ has_single_sbp(?w, ?sbp) ^ swrlb:greaterThan(?sbp, 180) -> has_abnormal_warning(?w, "severe high BP") |
|  | **HTN-abnormal_warning-06** | patient_profile(?p) ^ has_disease(?p, Hypertension) ^ has_pathway_task(?p, ?w) ^ abnormal_warning(?w) ^ has_single_dbp(?w, ?dbp) ^ swrlb:greaterThan(?dbp, 110) -> has_abnormal_warning(?w, "severe high BP") |
|  | **HTN-abnormal_warning-07** | patient_profile(?p) ^ has_disease(?p, Hypertension) ^ has_pathway_task(?p, ?w) ^ abnormal_warning(?w) ^ has_single_sbp(?w, ?sbp) ^ swrlb:lessThan(?sbp, 90) -> has_abnormal_warning(?w, "low BP") |
|  | **HTN-abnormal_warning-08** | patient_profile(?p) ^ has_disease(?p, Hypertension) ^ has_pathway_task(?p, ?w) ^ abnormal_warning(?w) ^ has_single_dbp(?w, ?dbp) ^ swrlb:lessThan(?dbp, 60) -> has_abnormal_warning(?w, "low BP") |
|  | **HTN-abnormal_warning-09** | patient_profile(?p) ^ has_disease(?p, Hypertension) ^ has_pathway_task(?p, ?w) ^ abnormal_warning(?w) ^ has_weekly_sbp(?w, ?sbp) ^ swrlb:greaterThan(?sbp, 160) -> has_abnormal_warning(?w, "high weekly BP") |
|  | **HTN-abnormal_warning-10** | patient_profile(?p) ^ has_disease(?p, Hypertension) ^ has_pathway_task(?p, ?w) ^ abnormal_warning(?w) ^ has_weekly_dbp(?w, ?dbp) ^ swrlb:greaterThan(?dbp, 100) -> has_abnormal_warning(?w, "high weekly BP") |
|  | **HTN-abnormal_warning-11** | patient_profile(?p) ^ has_disease(?p, Hypertension) ^ has_pathway_task(?p, ?w) ^ abnormal_warning(?w) ^ has_monthly_sbp(?w, ?sbp) ^ swrlb:greaterThan(?sbp, 140) -> has_abnormal_warning(?w, "high monthly BP") |
|  | **HTN-abnormal_warning-12** | patient_profile(?p) ^ has_disease(?p, Hypertension) ^ has_pathway_task(?p, ?w) ^ abnormal_warning(?w) ^ has_monthly_dbp(?w, ?dbp) ^ swrlb:greaterThan(?dbp, 90) -> has_abnormal_warning(?w, "high monthly BP") |
|  | **HTN-abnormal_warning-13** | patient_profile(?p) ^ has_disease(?p, Hypertension) ^ has_pathway_task(?p, ?w) ^ abnormal_warning(?w) ^ has_heart_rate(?w, ?hr) ^ swrlb:greaterThan(?hr, 100) -> has_abnormal_warning(?w, "abnormal HR") |
|  | **HTN-abnormal_warning-14** | patient_profile(?p) ^ has_disease(?p, Hypertension) ^ has_pathway_task(?p, ?w) ^ abnormal_warning(?w) ^ has_heart_rate(?w, ?hr) ^ swrlb:lessThan(?hr, 50) -> has_abnormal_warning(?w, "abnormal HR") |
|  | **HTN-abnormal_warning-15** | patient_profile(?p) ^ has_disease(?p, Hypertension) ^ has_pathway_task(?p, ?w) ^ abnormal_warning(?w) ^ has_discomfort(?w, true) -> has_abnormal_warning(?w, "Discomfort") |
| **Compliance Management** | **HTN-compliance_management-01** | patient_profile(?p) ^ has_disease(?p, Hypertension) ^ has_pathway_task(?p, ?c) ^ compliance_management(?c) ^ has_management_level(?c, 1) ^ has_actual_bp_frequency(?c, ?f) ^ swrlb:divide(?res, ?f, 7) ^ swrlb:greaterThanOrEqual(?res, 1) -> has_compliance_value(?c, 1) ^ has_compliance_level(?c, 5) |
|  | **HTN-compliance_management-02** | patient_profile(?p) ^ has_disease(?p, Hypertension) ^ has_pathway_task(?p, ?c) ^ compliance_management(?c) ^ has_management_level(?c, 1) ^ has_actual_bp_frequency(?c, ?f) ^ swrlb:divide(?res, ?f, 7) ^ swrlb:greaterThan(?res, 0.8) ^ swrlb:lessThan(?res, 1) -> has_compliance_value(?c, ?res) ^ has_compliance_level(?c, 4) |
|  | **HTN-compliance_management-03** | patient_profile(?p) ^ has_disease(?p, Hypertension) ^ has_pathway_task(?p, ?c) ^ compliance_management(?c) ^ has_management_level(?c, 1) ^ has_actual_bp_frequency(?c, ?f) ^ swrlb:divide(?res, ?f, 7) ^ swrlb:greaterThan(?res, 0.5) ^ swrlb:lessThanOrEqual(?res, 0.8) -> has_compliance_value(?c, ?res) ^ has_compliance_level(?c, 3) |
|  | **HTN-compliance_management-04** | patient_profile(?p) ^ has_disease(?p, Hypertension) ^ has_pathway_task(?p, ?c) ^ compliance_management(?c) ^ has_management_level(?c, 1) ^ has_actual_bp_frequency(?c, ?f) ^ swrlb:divide(?res, ?f, 7) ^ swrlb:greaterThan(?res, 0) ^ swrlb:lessThanOrEqual(?res, 0.5) -> has_compliance_value(?c, ?res) ^ has_compliance_level(?c, 2) |
|  | **HTN-compliance_management-05** | patient_profile(?p) ^ has_disease(?p, Hypertension) ^ has_pathway_task(?p, ?c) ^ compliance_management(?c) ^ has_management_level(?c, 1) ^ has_actual_bp_frequency(?c, ?f) ^ swrlb:divide(?res, ?f, 7) ^ swrlb:equal(?res, 0) ^ -> has_compliance_value(?c, 0) ^ has_compliance_level(?c, 1) |
|  | **HTN-compliance_management-06** | patient_profile(?p) ^ has_disease(?p, Hypertension) ^ has_pathway_task(?p, ?c) ^ compliance_management(?c) ^ has_management_level(?c, 2) ^ has_actual_bp_frequency(?c, ?f) ^ swrlb:divide(?res, ?f, 14) ^ swrlb:greaterThanOrEqual(?res, 1) -> has_compliance_value(?c, 1) ^ has_compliance_level(?c, 5) |
|  | **HTN-compliance_management-07** | patient_profile(?p) ^ has_disease(?p, Hypertension) ^ has_pathway_task(?p, ?c) ^ compliance_management(?c) ^ has_management_level(?c, 2) ^ has_actual_bp_frequency(?c, ?f) ^ swrlb:divide(?res, ?f, 14) ^ swrlb:greaterThan(?res, 0.8) ^ swrlb:lessThan(?res, 1) -> has_compliance_value(?c, ?res) ^ has_compliance_level(?c, 4) |
|  | **HTN-compliance_management-08** | patient_profile(?p) ^ has_disease(?p, Hypertension) ^ has_pathway_task(?p, ?c) ^ compliance_management(?c) ^ has_management_level(?c, 2) ^ has_actual_bp_frequency(?c, ?f) ^ swrlb:divide(?res, ?f, 14) ^ swrlb:greaterThan(?res, 0.5) ^ swrlb:lessThanOrEqual(?res, 0.8) -> has_compliance_value(?c, ?res) ^ has_compliance_level(?c, 3) |
|  | **HTN-compliance_management-09** | patient_profile(?p) ^ has_disease(?p, Hypertension) ^ has_pathway_task(?p, ?c) ^ compliance_management(?c) ^ has_management_level(?c, 2) ^ has_actual_bp_frequency(?c, ?f) ^ swrlb:divide(?res, ?f, 14) ^ swrlb:greaterThan(?res, 0) ^ swrlb:lessThanOrEqual(?res, 0.5) -> has_compliance_value(?c, ?res) ^ has_compliance_level(?c, 2) |
|  | **HTN-compliance_management-10** | patient_profile(?p) ^ has_disease(?p, Hypertension) ^ has_pathway_task(?p, ?c) ^ compliance_management(?c) ^ has_management_level(?c, 2) ^ has_actual_bp_frequency(?c, ?f) ^ swrlb:divide(?res, ?f, 14) ^ swrlb:equal(?res, 0) -> has_compliance_value(?c, 0) ^ has_compliance_level(?c, 1) |

**Table 4**. Management plan rule for hypertension.

| Category | Rule Number | SWRL Rule |
| --- | --- | --- |
| **Management Plan** | **HTN-management_plan-01** | patient_profile(?p) ^ has_disease(?p, Hypertension) ^ has_management_plan(?p, ?mp) ^ has_sub_plan(?mp, ?sp) ^ selfmanagement_plan(?sp) ^ has_sub_plan(?sp, ?mop) ^ selfmonitoring_subplan(?mop) ^ has_monitoring_item(?mop, ?bp) ^ blood_pressure(?bp) ^ has_pathway_task(?p, ?h) ^ hierarchical_management(?h) ^ has_management_level(?h, ?level) ^ swrlb:equal(?level, 1) -> has_control_target(?bp, "140/90 mmHg") ^ has_monitoring_frequency(?bp, "once per day") |
|  | **HTN-management_plan-02** | patient_profile(?p) ^ has_disease(?p, Hypertension) ^ has_management_plan(?p, ?mp) ^ has_sub_plan(?mp, ?sp) ^ selfmanagement_plan(?sp) ^ has_sub_plan(?sp, ?mop) ^ selfmonitoring_subplan(?mop) ^ has_monitoring_item(?mop, ?hr) ^ heart_rate(?hr) ^ has_pathway_task(?p, ?h) ^ hierarchical_management(?h) ^ has_management_level(?h, ?level) ^ swrlb:equal(?level, 1) -> has_control_target(?hr, "50-100 bpm") ^ has_monitoring_frequency(?hr, "once per day") |
|  | **HTN-management_plan-03** | patient_profile(?p) ^ has_disease(?p, Hypertension) ^ has_management_plan(?p, ?mp) ^ has_sub_plan(?mp, ?sp) ^ selfmanagement_plan(?sp) ^ has_sub_plan(?sp, ?mop) ^ selfmonitoring_subplan(?mop) ^ has_monitoring_item(?mop, ?we) ^ weight(?we) -> has_control_target(?we, "BMI < 24") ^ has_monitoring_frequency(?we, "once per day") |
|  | **HTN-management_plan-04** | patient_profile(?p) ^ has_disease(?p, Hypertension) ^ has_management_plan(?p, ?mp) ^ has_sub_plan(?mp, ?sp) ^ selfmanagement_plan(?sp) ^ has_sub_plan(?sp, ?mop) ^ selfmonitoring_subplan(?mop) ^ has_monitoring_item(?mop, ?bp) ^ blood_pressure(?bp) ^ has_pathway_task(?p, ?h) ^ hierarchical_management(?h) ^ has_management_level(?h, ?level) ^ swrlb:equal(?level, 2) -> has_control_target(?bp, "140/90 mmHg") ^ has_monitoring_frequency(?bp, "twice per day") |
|  | **HTN-management_plan-05** | patient_profile(?p) ^ has_disease(?p, Hypertension) ^ has_management_plan(?p, ?mp) ^ has_sub_plan(?mp, ?sp) ^ selfmanagement_plan(?sp) ^ has_sub_plan(?sp, ?mop) ^ selfmonitoring_subplan(?mop) ^ has_monitoring_item(?mop, ?hr) ^ heart_rate(?hr) ^ has_pathway_task(?p, ?h) ^ hierarchical_management(?h) ^ has_management_level(?h, ?level) ^ swrlb:equal(?level, 2) -> has_control_target(?hr, "50-100 bpm") ^ has_monitoring_frequency(?hr, "twice per day") |
|  | **HTN-management_plan-06** | patient_profile(?p) ^ has_disease(?p, Hypertension) ^ has_management_plan(?p, ?mp) ^ has_sub_plan(?mp, ?sp) ^ selfmanagement_plan(?sp) ^ has_sub_plan(?sp, ?mep) ^ drug_subplan(?mep) ^ has_pathway_task(?p, ?m) ^ medication_guidance(?m) ^ has_therapy_recommendation(?m, ?r) -> has_therapy_recommendation(?mep, ?r) |
|  | **HTN-management_plan-07** | patient_profile(?p) ^ has_disease(?p, Hypertension) ^ has_management_plan(?p, ?mp) ^ has_sub_plan(?mp, ?sp) ^ selfmanagement_plan(?sp) ^ has_sub_plan(?sp, ?lp) ^ lifestyle_subplan(?lp) ^ has_pathway_task(?p, ?lg) ^ lifestyle_guidance(?lg) ^ has_lifestyle_suggestion(?lg, ?ls) -> has_lifestyle_suggestion(?lp, ?ls) |
|  | **HTN-management_plan-08** | patient_profile(?p) ^ has_disease(?p, Hypertension) ^ has_management_plan(?p, ?mp) ^ has_sub_plan(?mp, ?ip) ^ intervention_plan(?ip) ^ has_sub_plan(?ip, ?gp) ^ GP_intervention_plan(?gp) ^ has_pathway_task(?p, ?f) ^ regular_followup(?f) ^ time:hasTime(?f, ?t) ^ has_new_followup_interval(?t, ?fi) -> has_new_followup_interval(?gp, ?fi) |
|  | **HTN-management_plan-09** | patient_profile(?p) ^ has_disease(?p, Hypertension) ^ has_management_plan(?p, ?mp) ^ has_sub_plan(?mp, ?ip) ^ intervention_plan(?ip) ^ has_sub_plan(?ip, ?gp) ^ GP_intervention_plan(?gp) ^ has_pathway_task(?p, ?w) ^ abnormal_warning(?w) ^ has_abnormal_warning(?w, ?aw) -> has_abnormal_warning(?gp, ?aw) |
|  | **HTN-management_plan-10** | patient_profile(?p) ^ has_disease(?p, Hypertension) ^ has_management_plan(?p, ?mp) ^ has_sub_plan(?mp, ?ip) ^ intervention_plan(?ip) ^ has_sub_plan(?ip, ?cm) ^ CM_intervention_plan(?cm) ^ has_pathway_task(?p, ?c) ^ compliance_management(?c) ^ has_compliance_level(?c, ?cl) ^ swrlb:lessThan(?c, 3) -> has_compliance_intervention(?cm, true) |
|  | **HTN-management_plan-11** | patient_profile(?p) ^ has_disease(?p, Hypertension) ^ has_management_plan(?p, ?mp) ^ has_sub_plan(?mp, ?ip) ^ intervention_plan(?ip) ^ has_sub_plan(?ip, ?cm) ^ CM_intervention_plan(?cm) ^ has_pathway_task(?p, ?c) ^ compliance_management(?c) ^ has_compliance_level(?c, ?cl) ^ swrlb:greaterThanOrEqual(?cl, 3) -> has_compliance_intervention(?cm, false) |
