# Supplementary material for: Pathway-Driven Coordinated Telehealth System for Management of Patients With Single or Multiple Chronic Diseases in China: System Development and Retrospective Study
Source: JMIR Med Inform. 2021 May 17;9(5):e27228. doi: 10.2196/27228 (PMC8167615; doi:10.2196/27228)

**Detailed screenshots of the web platform for care providers**

In this supplementary material, we present the detailed screenshots of the web platform for care providers.

**Patient Registration**

**
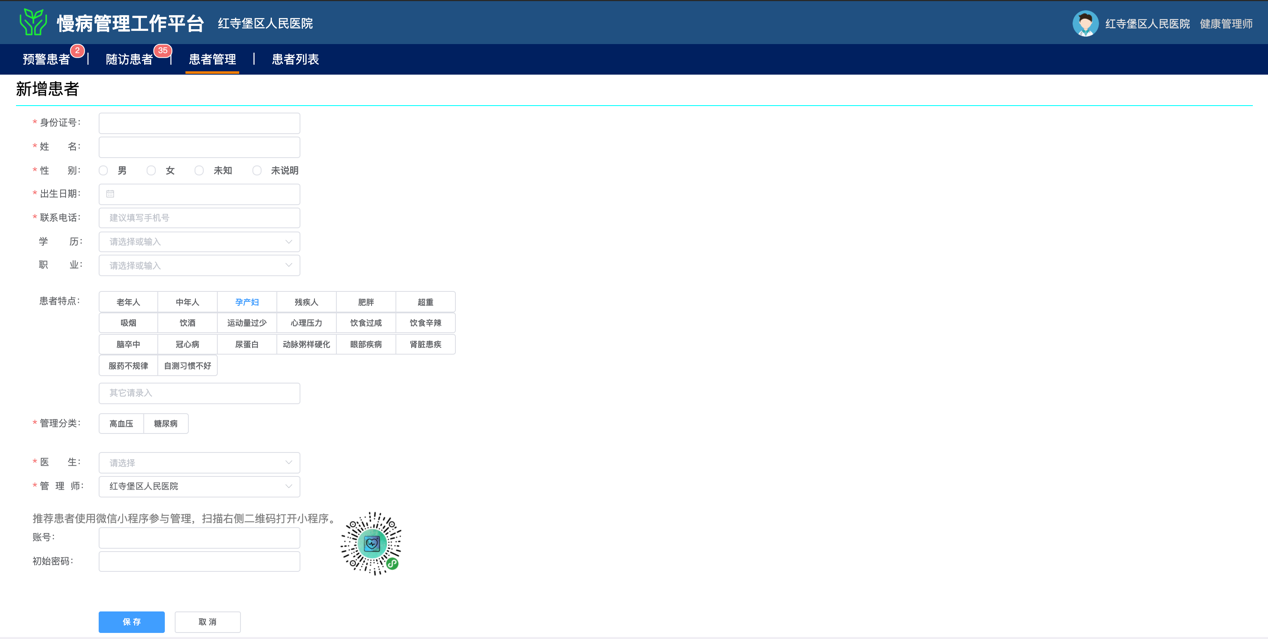
**

**Patient Warning**


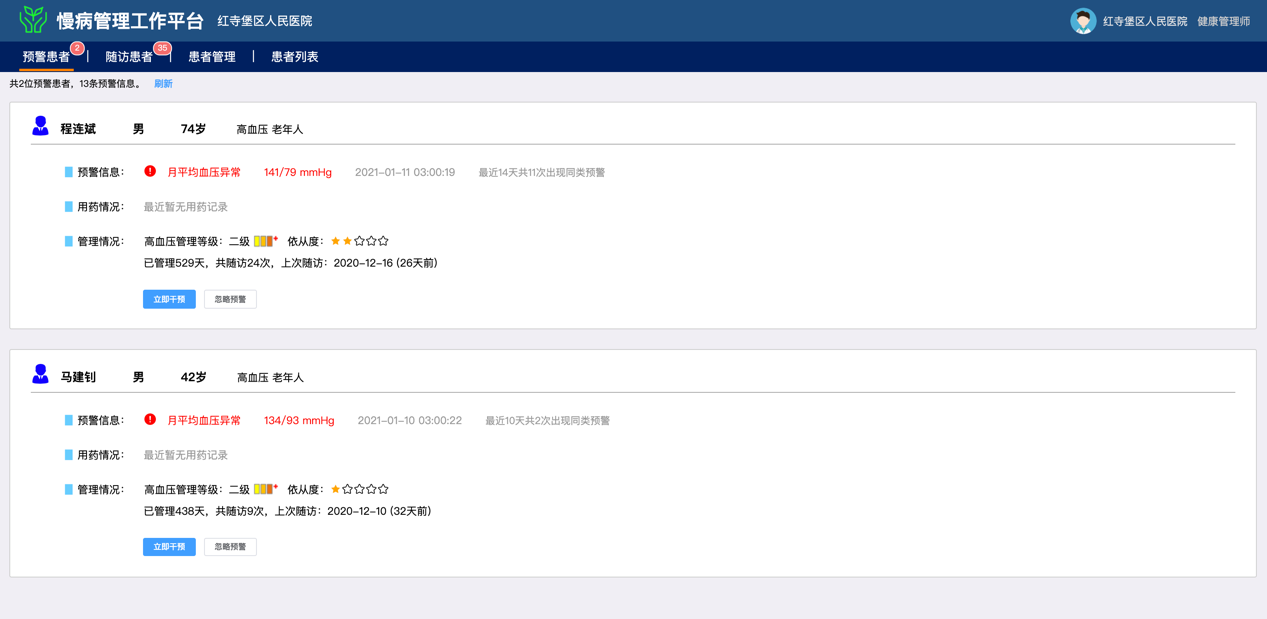


**Patient Follow-up**


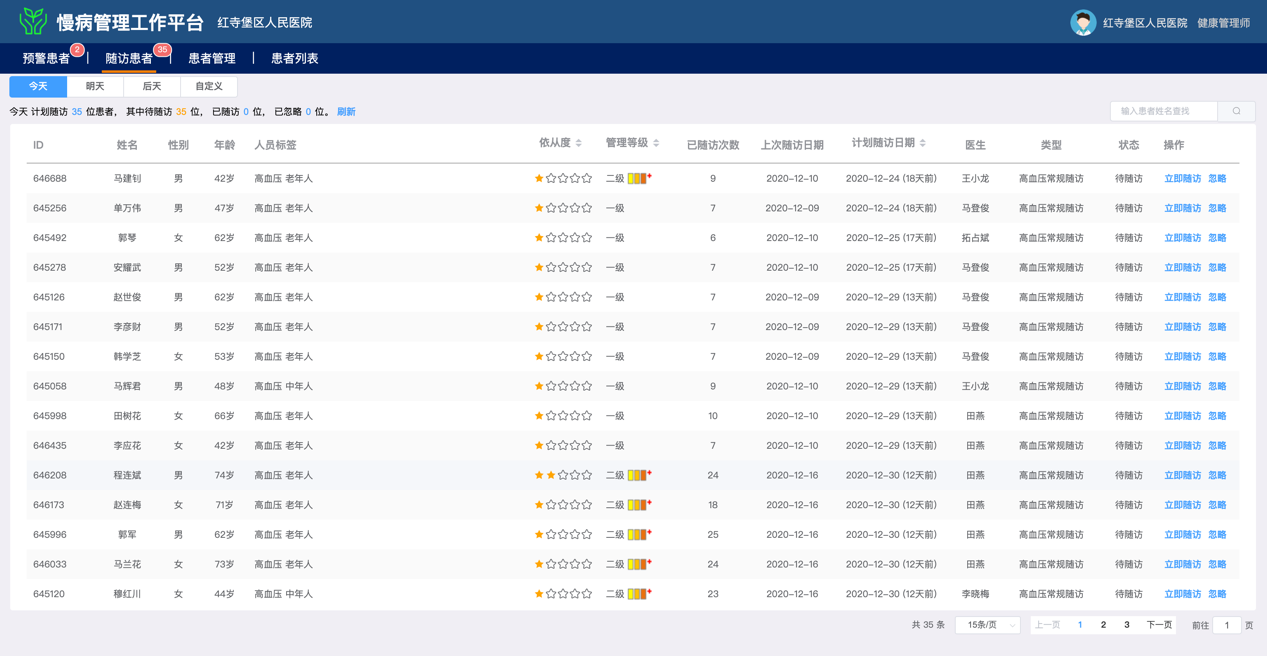


**Patient List**


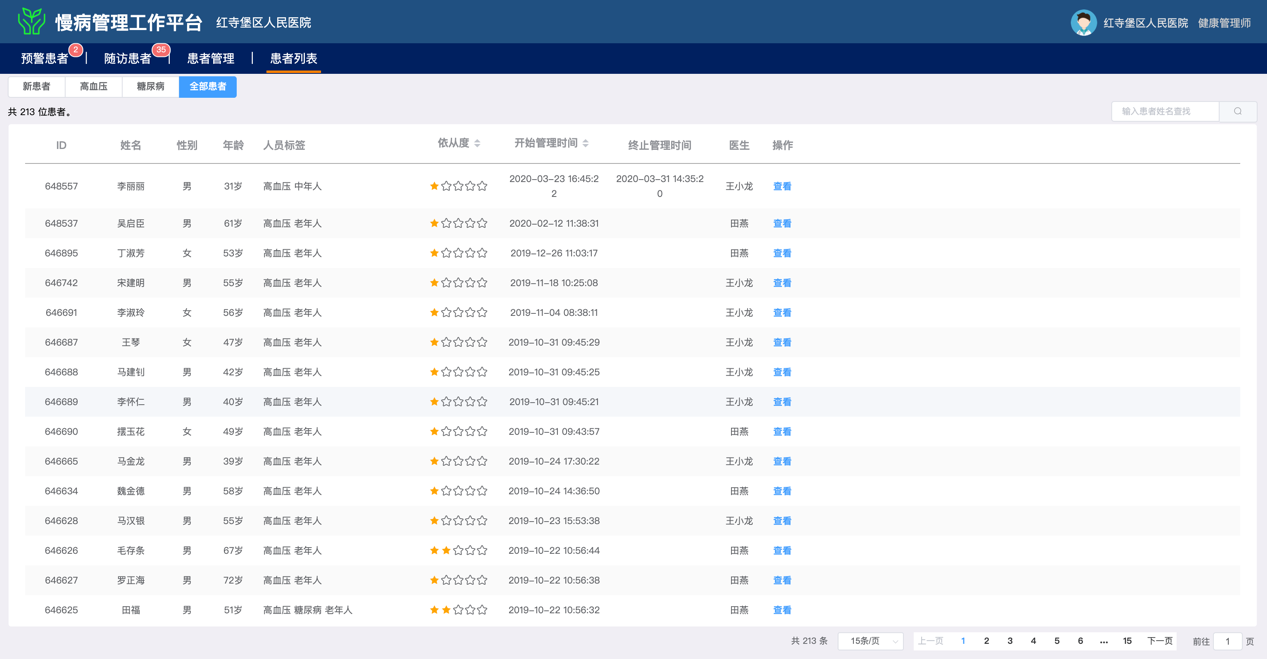


**Patient Information & Intervention**


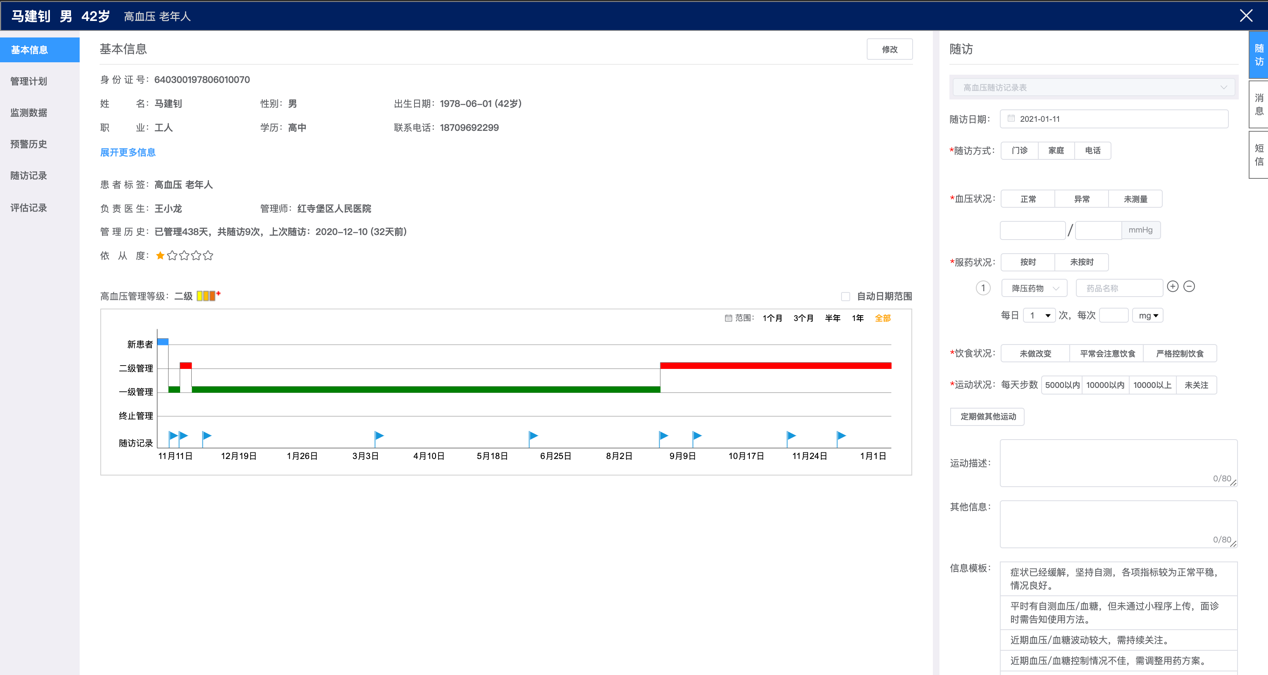


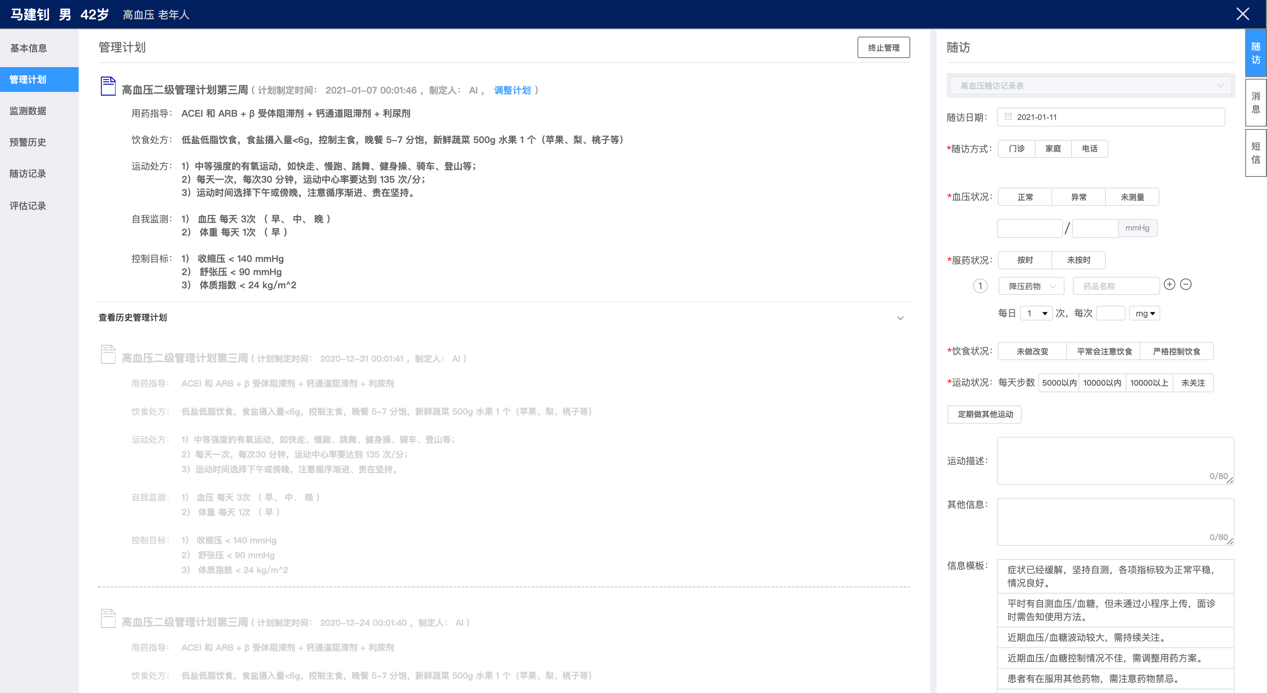


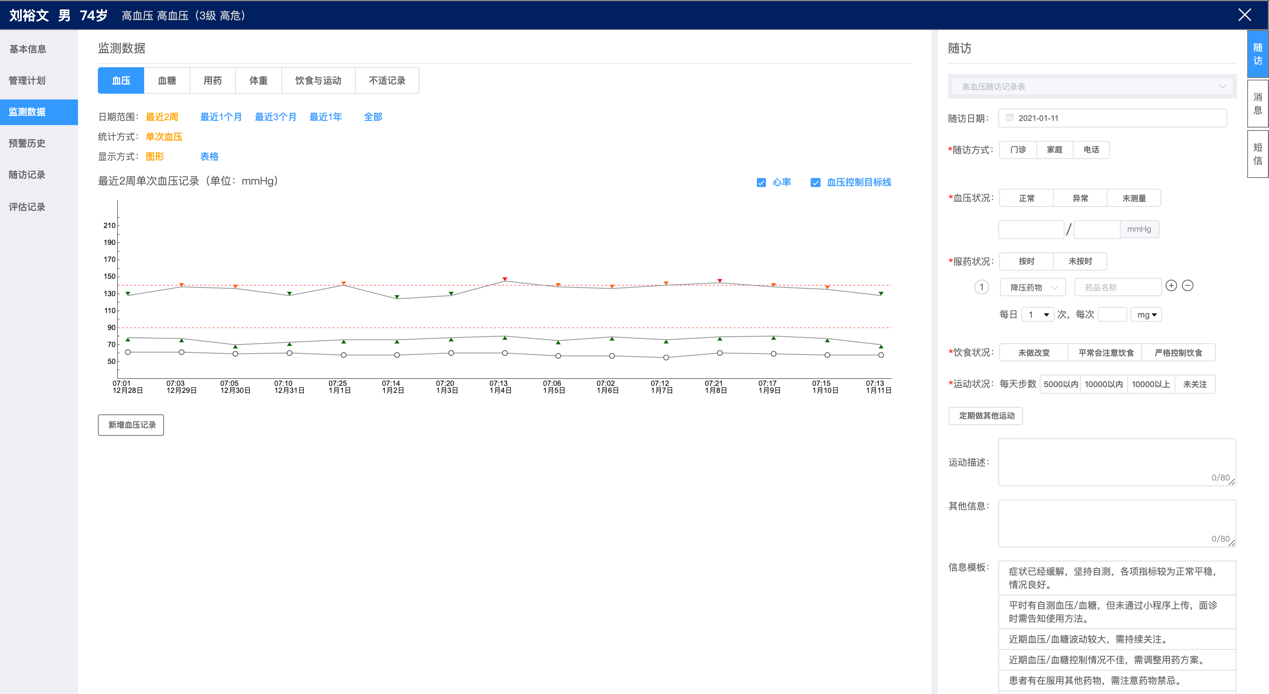


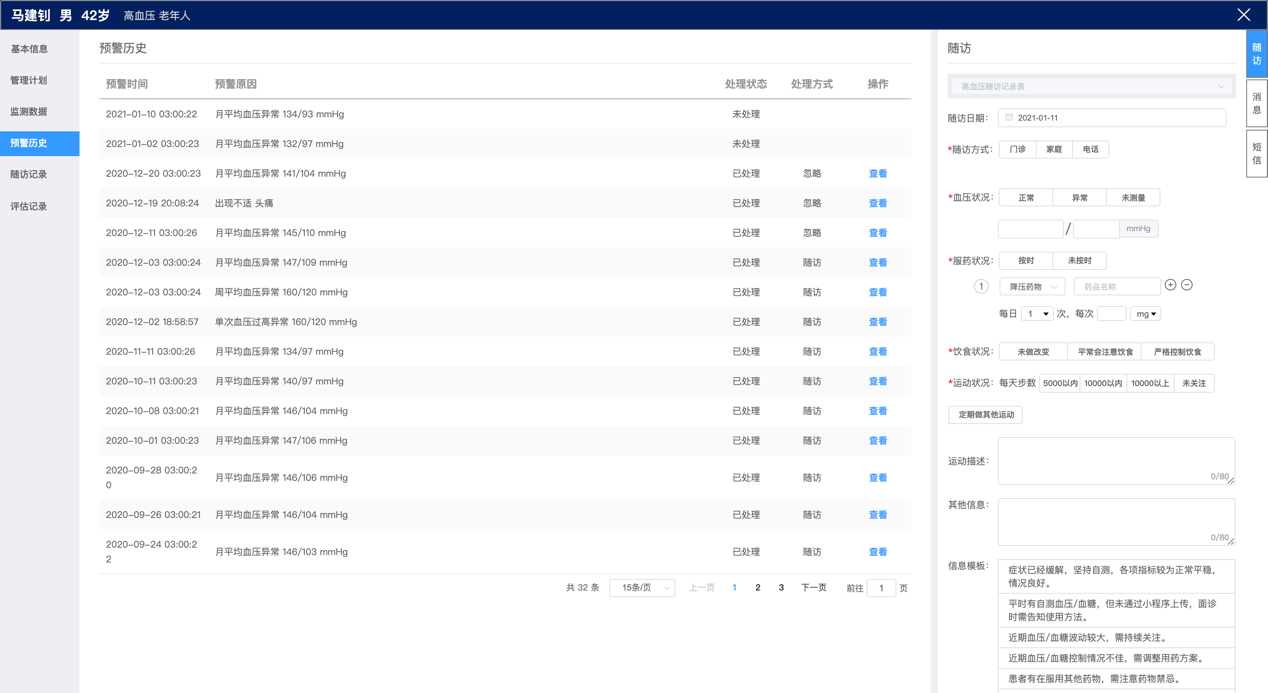


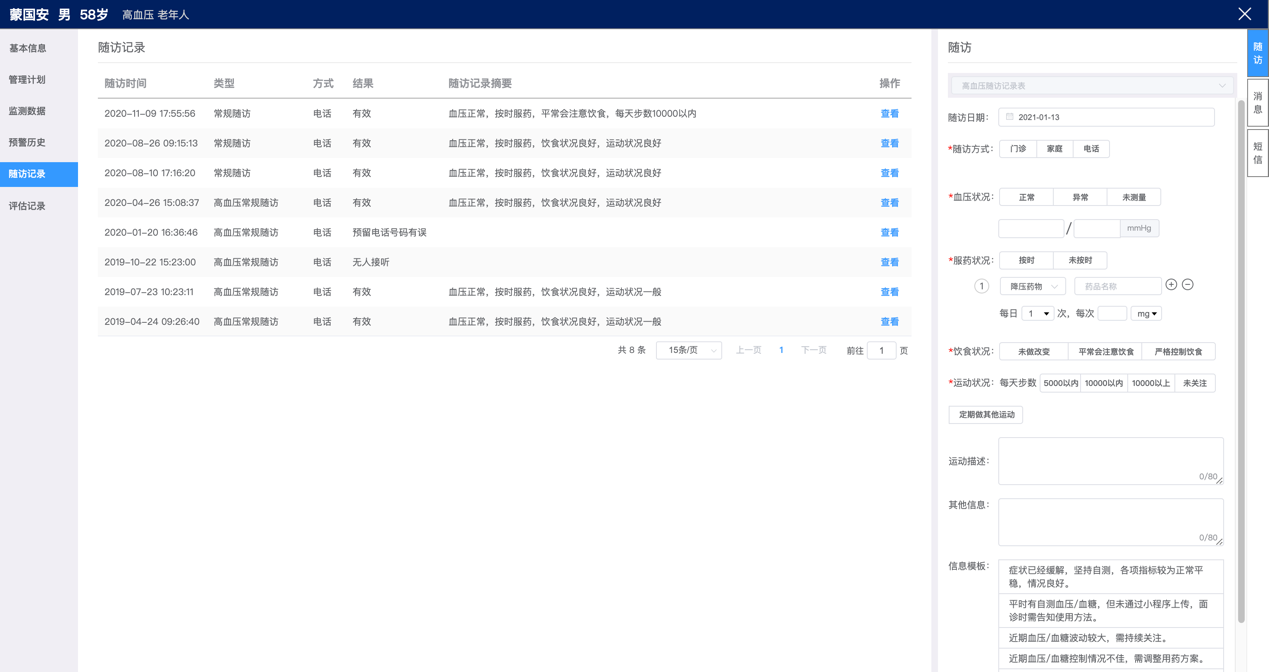


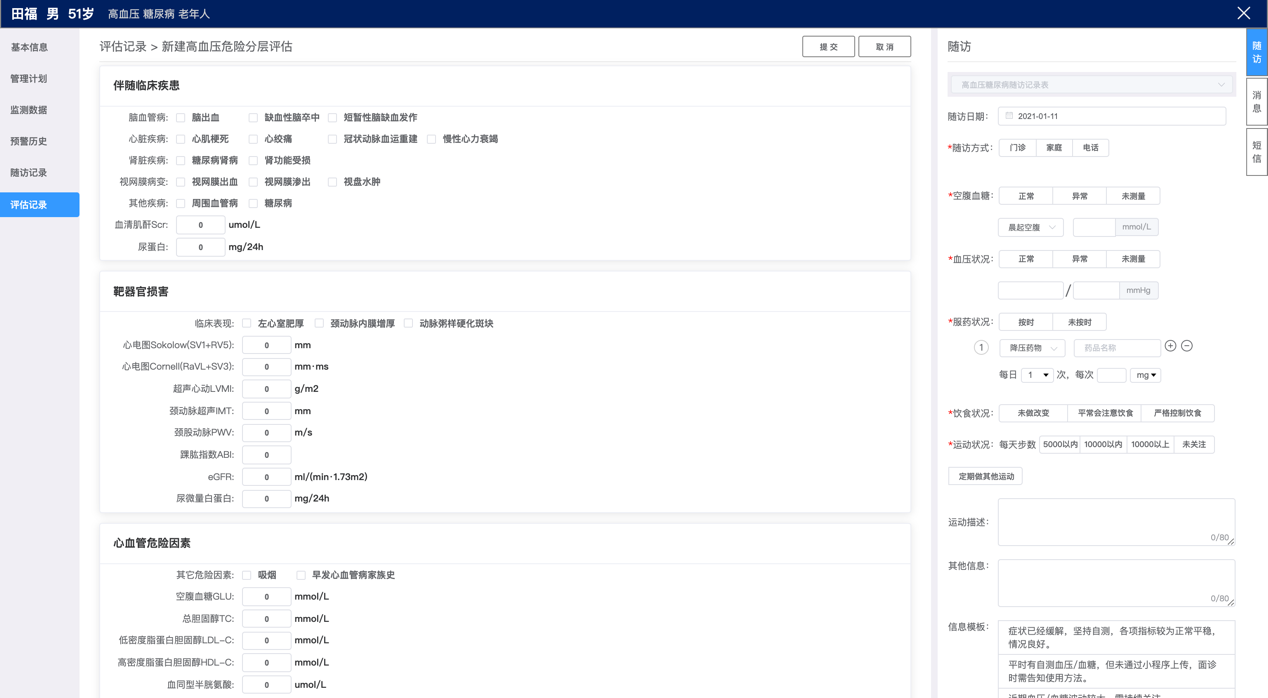

Supplement: Multimedia Appendix 3 [file medinform_v9i5e27228_app3.docx]
