# Supplementary material for: Pathway-Driven Coordinated Telehealth System for Management of Patients With Single or Multiple Chronic Diseases in China: System Development and Retrospective Study
Source: JMIR Med Inform. 2021 May 17;9(5):e27228. doi: 10.2196/27228 (PMC8167615; doi:10.2196/27228)
Supplement: Multimedia Appendix 4 [file medinform_v9i5e27228_app4.docx]

**Detailed screenshots of the mobile app for patients**

In this supplementary material, we present the detailed screenshots of the mobile app for patients. As mentioned in the main text, due to the distinction between HTN & T2DM care pathway and COPD care pathway, we applied different user interface design to the app for HTN & T2DM and the app for COPD. The two apps shared the same underlying framework and provided similar functional modules.

**Client for HTN & T2DM**

**Management Plan & Self-monitoring**


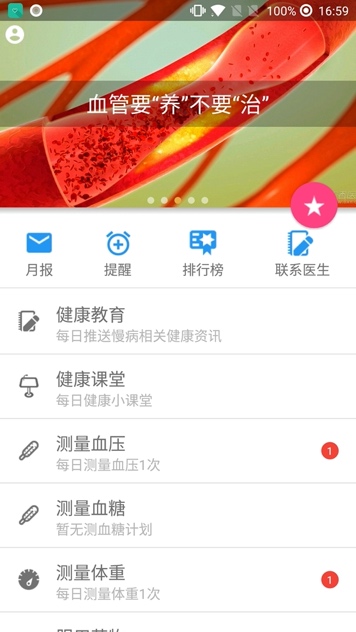

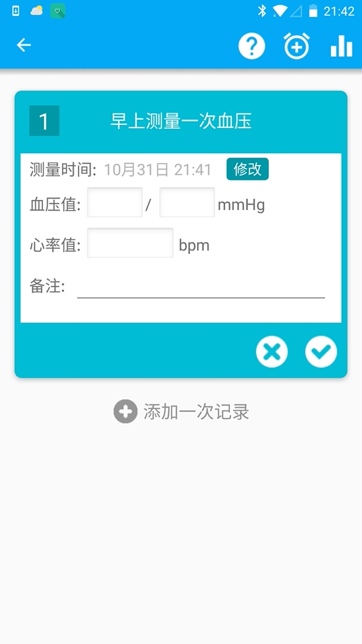

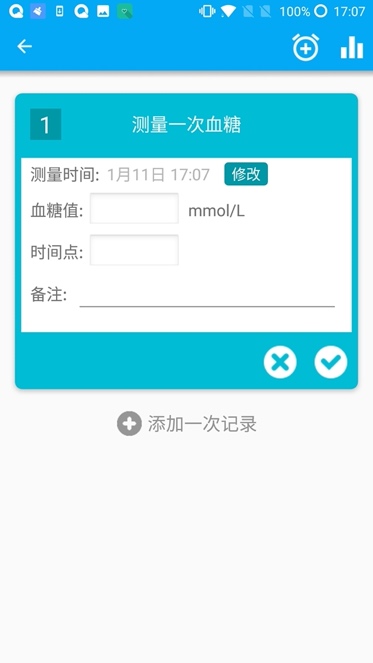


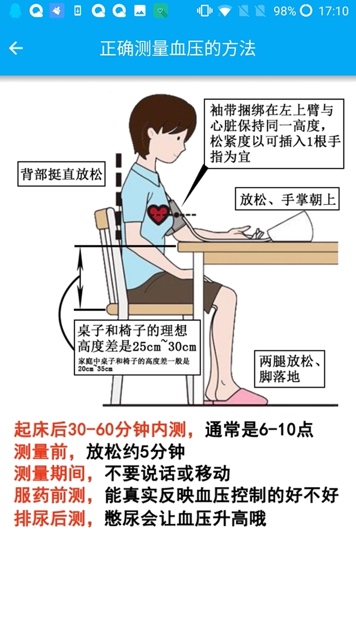

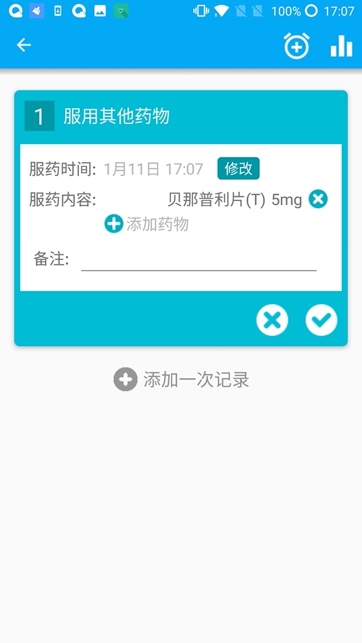

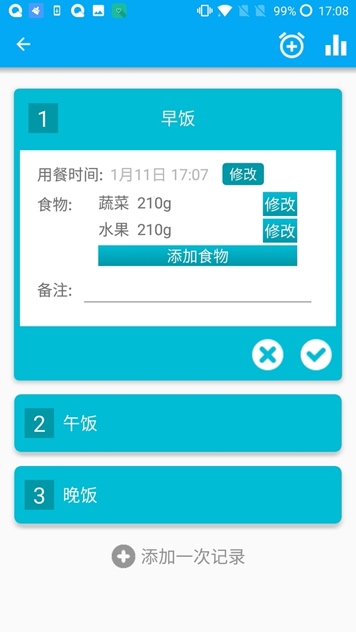


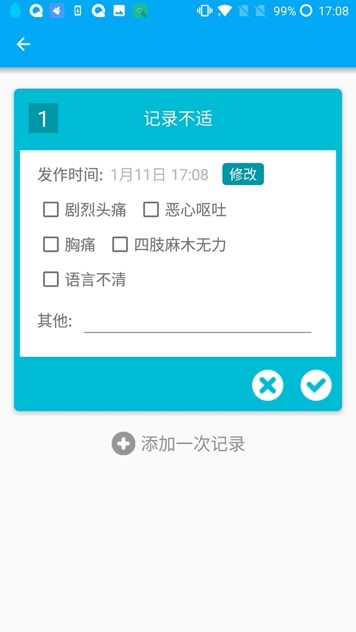

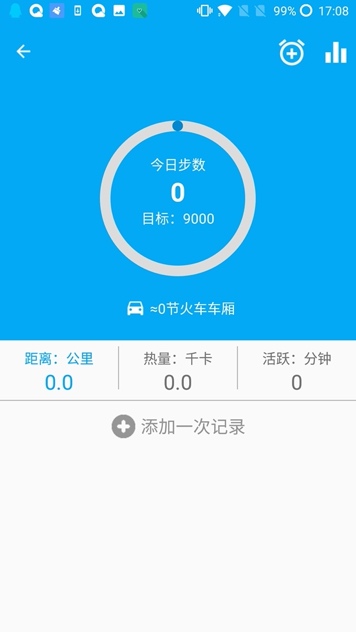


**Health Check-up**

**
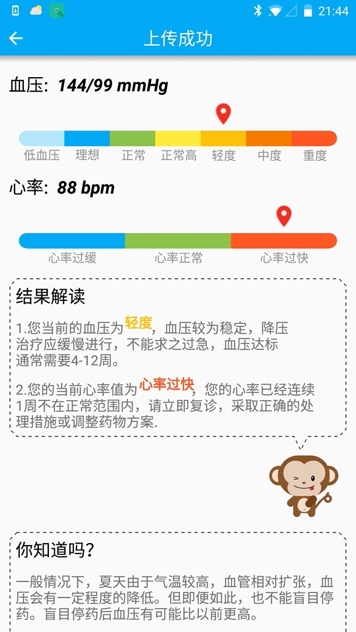
**

**Health Report**

**
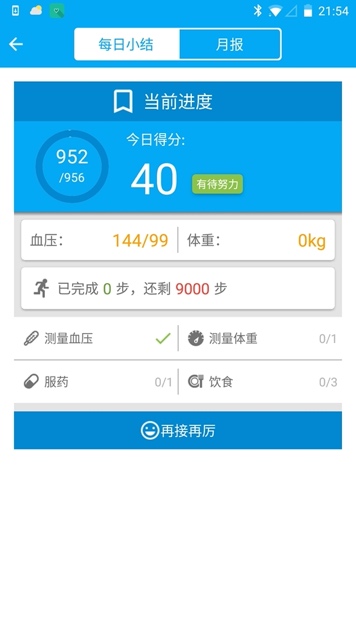

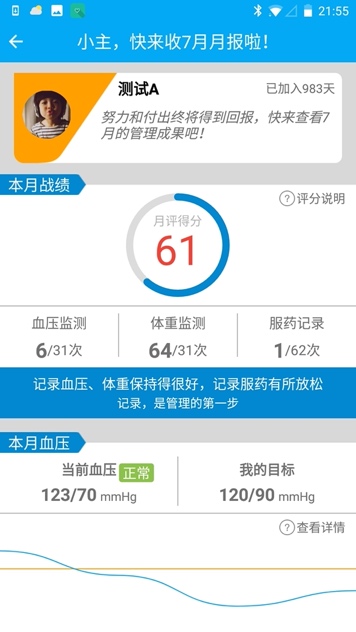

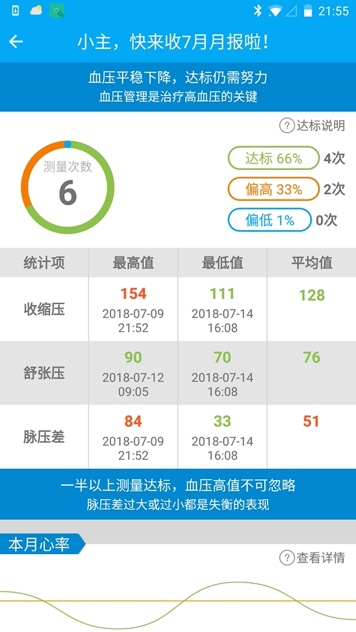
**

**
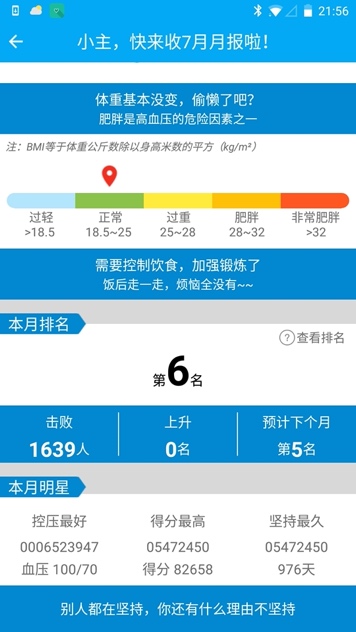
**

**Reminder Service**

**
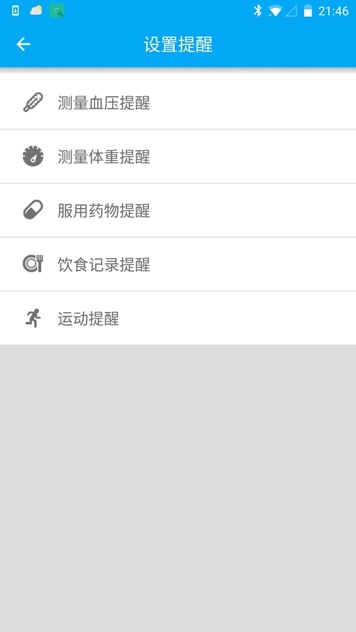
**

**Health Education**

**
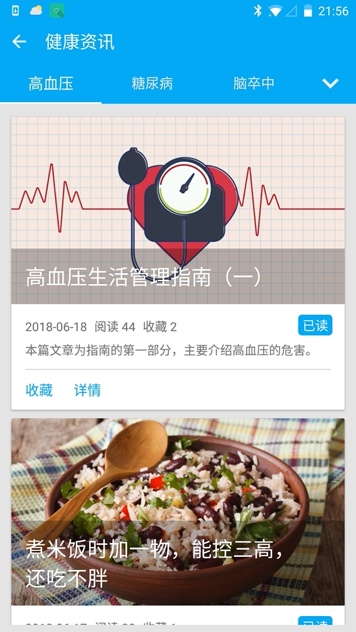

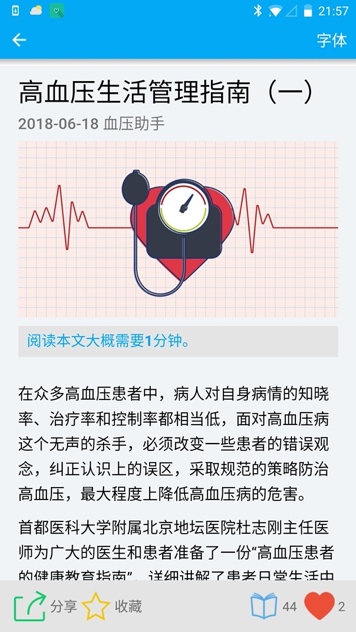

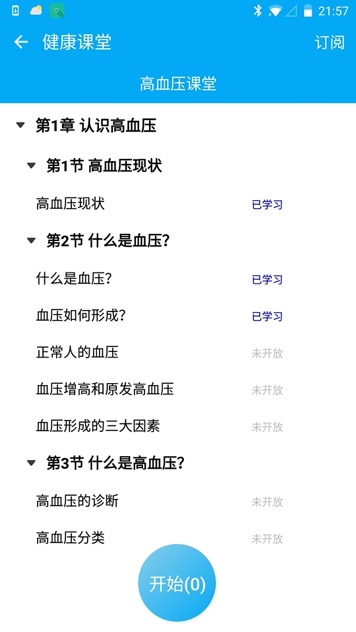
**

**Client for COPD**

**Management Plan & Self-monitoring**

**
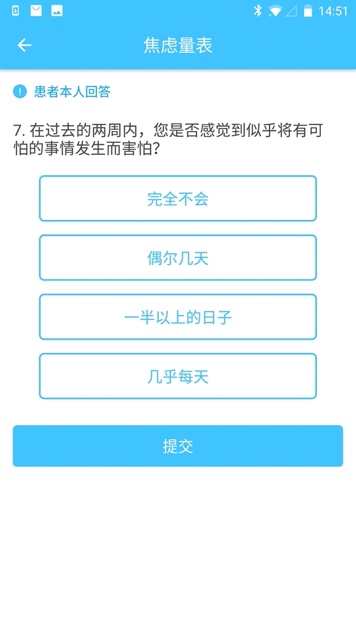

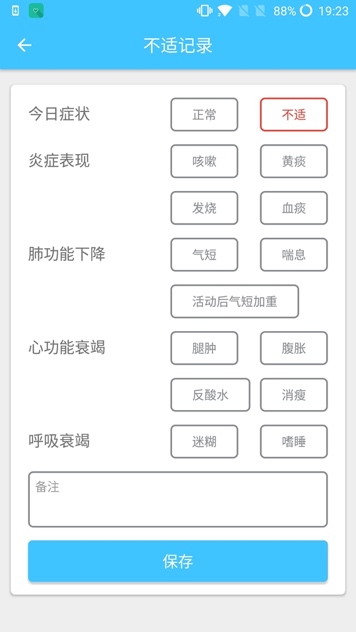

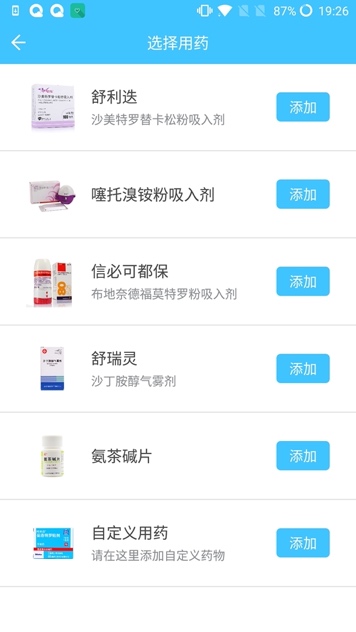

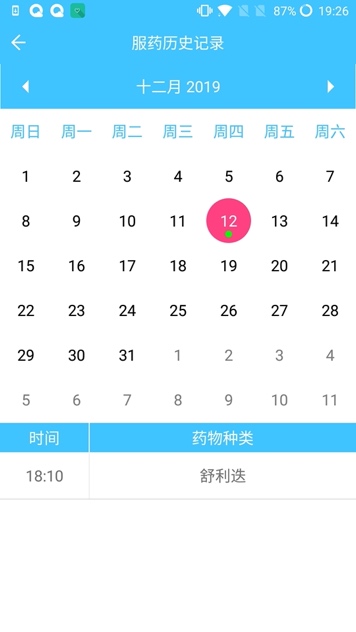

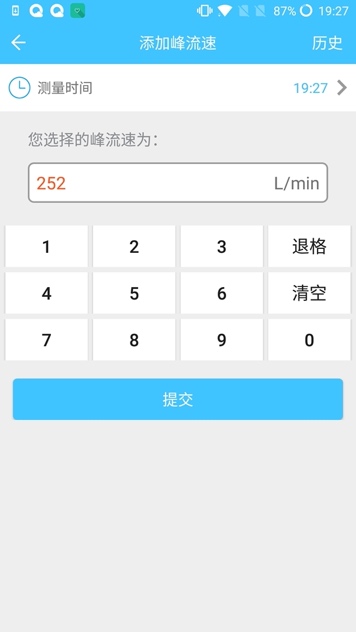

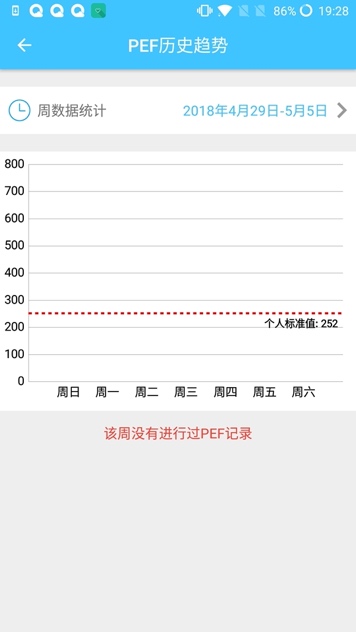

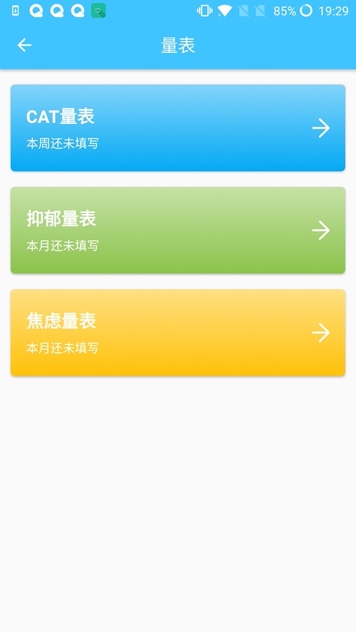
**

**Health Check-up**

**
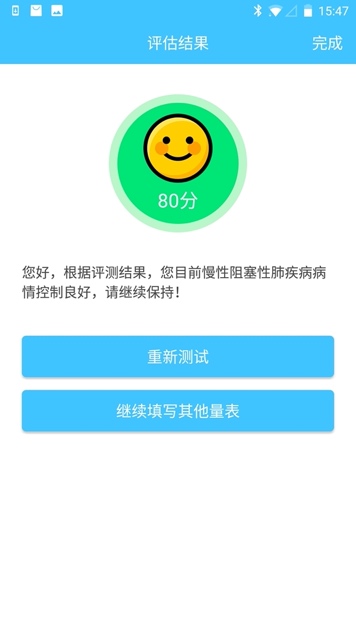
**

**Health Report**

**
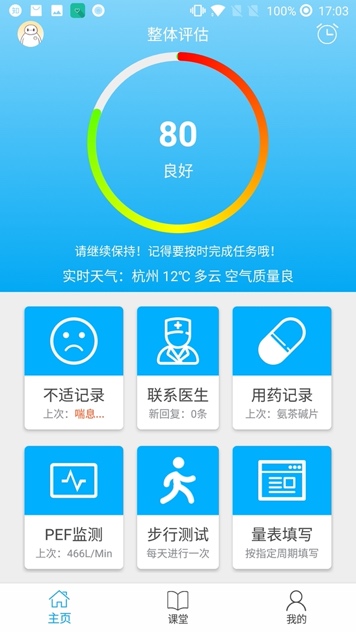
**

**Reminder Service**

**
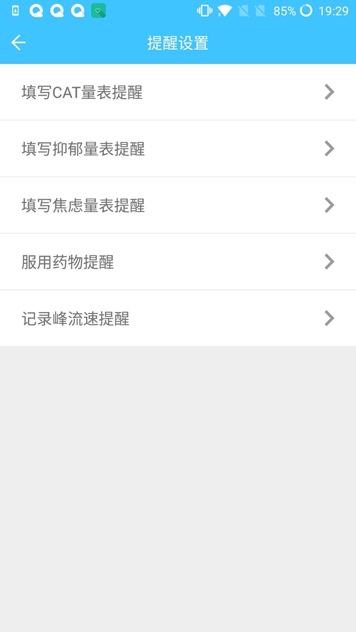
**

**Health Education**

**
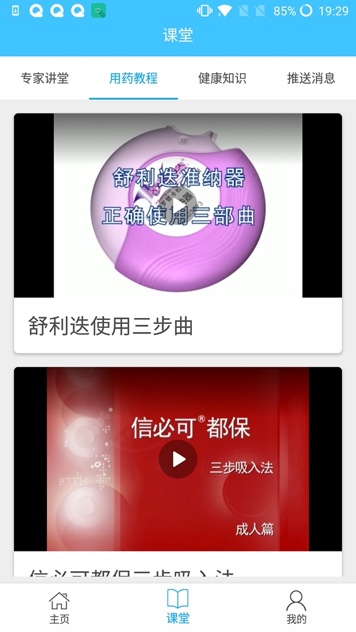
**
