# Supplementary material for: Pathway-Driven Coordinated Telehealth System for Management of Patients With Single or Multiple Chronic Diseases in China: System Development and Retrospective Study
Source: JMIR Med Inform. 2021 May 17;9(5):e27228. doi: 10.2196/27228 (PMC8167615; doi:10.2196/27228)
Supplement: Multimedia Appendix 5 [file medinform_v9i5e27228_app5.docx]

**Subgroup analysis on comparison of patient outcomes**

In this supplementary material, we provide a detailed subgroup analysis on comparison of patient outcomes over different time spans. Concretely, we conducted three types of subgroup analyses as follows:

1. patients with different diseases (single disease and multiple chronic conditions) for the systolic blood pressure (SBP) and fast blood glucose (FBG).

2. patients in different gender for SBP, FBG, and peak expiratory flow (PEF).

3. patients in different age for SBP, FBG, and PEF. For the age, we divided the patients into three categories: young (age <= 45), middle age (45 < age <= 69), and old (age > 69).

**Table 1**. Comparison of patient outcomes over different time spans for patient with different diseases.

| Patient outcome | Disease | Period  (days) | Number of patients  who have records | Mean value  (before) | Mean value  (after) | *P* value |
| --- | --- | --- | --- | --- | --- | --- |
| SBP^a^  (mmHg) | HTN^d^ | 30 | 1,056 | 132.06 | 127.8 | **<0.001** |
|  | HM^e^ |  | 63 | 132.71 | 130.69 | 0.231 |
|  | HTN | 60 | 808 | 131.68 | 128.16 | **<0.001** |
|  | HM |  | 59 | 132.25 | 130.24 | 0.379 |
|  | HTN | 90 | 937 | 130.36 | 127.8 | **<0.001** |
|  | HM |  | 55 | 132.56 | 133.82 | 0.485 |
|  | HTN | 120 | 678 | 132.4 | 128.59 | **<0.001** |
|  | HM |  | 33 | 136.82 | 135.52 | 0.649 |
|  | HTN | 150 | 416 | 130.57 | 126.98 | **<0.001** |
|  | HM |  | 20 | 136.22 | 140.78 | 0.333 |
|  | HTN | 180 | 380 | 130.6 | 127.4 | **<0.001** |
|  | HM |  | 17 | 139.25 | 143.61 | 0.366 |
| FBG^b^  (mmol/L) | T2DM^f^ | 30 | 32 | 6.74 | 6.88 | 0.672 |
|  | HM |  | 37 | 7.07 | 6.64 | 0.116 |
|  | T2DM | 60 | 414 | 4.93 | 4.73 | **<0.001** |
|  | HM |  | 39 | 6.89 | 6.48 | 0.165 |
|  | T2DM | 90 | 26 | 6.8 | 6.68 | 0.698 |
|  | HM |  | 27 | 6.63 | 6.37 | 0.112 |
|  | T2DM | 120 | 12 | 7.74 | 7.11 | 0.404 |
|  | HM |  | 10 | 7.03 | 6.7 | 0.225 |
|  | T2DM | 150 | 6 | 6.4 | 7.43 | 0.473 |
|  | HM |  | 4 | 7.93 | 7.81 | 0.795 |
|  | T2DM | 180 | 4 | 6.26 | 6.92 | 0.636 |
|  | HM |  | 3 | 8.49 | 7.23 | 0.125 |
| PEF^c^  (L/min) | COPD^g^ | 30 | 55 | 315.78 | 320.59 | 0.673 |
|  |  | 60 | 47 | 320.51 | 323.13 | 0.841 |
|  |  | 90 | 44 | 325.77 | 310.69 | 0.977 |
|  |  | 120 | 45 | 326.61 | 327.76 | 0.951 |
|  |  | 150 | 40 | 316.67 | 309.55 | 0.732 |
|  |  | 180 | 41 | 314.81 | 311.16 | 0.855 |

^a^SBP: systolic blood pressure.

^b^FBG: fast blood glucose.

^c^PEF: peak expiratory flow.

^d^HTN: hypertension.

^e^HM: Hypertension with type 2 diabetes mellitus.

^f^T2DM: type 2 diabetes mellitus.

^g^COPD: chronic obstructive pulmonary disease.

**Table 2**. Comparison of patient outcomes over different time spans for different age groups.

| Patient outcome | Age | Period  (days) | Number of patients  who have records | Mean value  (before) | Mean value  (after) | *P* value |
| --- | --- | --- | --- | --- | --- | --- |
| SBP^a^  (mmHg) | Young | 30 | 199 | 131.63 | 126.92 | **<0.001** |
|  | Middle |  | 829 | 131.89 | 127.85 | **<0.001** |
|  | Old |  | 107 | 134.33 | 131.01 | **0.004** |
|  | Young | 60 | 147 | 129.19 | 125.29 | **<0.001** |
|  | Middle |  | 894 | 129.17 | 126.11 | **<0.001** |
|  | Old |  | 219 | 126.71 | 125.28 | **0.037** |
|  | Young | 90 | 128 | 128.86 | 125.11 | **<0.001** |
|  | Middle |  | 672 | 130.68 | 127.62 | **<0.001** |
|  | Old |  | 205 | 130.83 | 131.81 | 0.174 |
|  | Young | 120 | 96 | 130.28 | 125.82 | **<0.001** |
|  | Middle |  | 510 | 132.72 | 128.57 | **<0.001** |
|  | Old |  | 114 | 133.48 | 132.82 | 0.585 |
|  | Young | 150 | 72 | 130.09 | 125.54 | **0.009** |
|  | Middle |  | 318 | 130.78 | 127.16 | **<0.001** |
|  | Old |  | 55 | 131.96 | 132.94 | 0.593 |
|  | Young | 180 | 57 | 132.01 | 127.81 | **0.011** |
|  | Middle |  | 293 | 130.43 | 127.01 | **<0.001** |
|  | Old |  | 52 | 132.74 | 135.3 | 0.181 |
| FBG^b^  (mmol/L) | Young | 30 | 11 | 6.55 | 6.35 | 0.58 |
|  | Middle |  | 52 | 7.02 | 6.87 | 0.57 |
|  | Old |  | 13 | 6.51 | 6.43 | 0.631 |
|  | Young | 60 | 22 | 5.62 | 6.17 | 0.506 |
|  | Middle |  | 295 | 5.19 | 4.93 | **<0.001** |
|  | Old |  | 140 | 4.87 | 4.64 | **<0.001** |
|  | Young | 90 | 4 | 6.4 | 7.35 | 0.116 |
|  | Middle |  | 43 | 6.76 | 6.5 | 0.174 |
|  | Old |  | 11 | 6.69 | 6.59 | 0.706 |
|  | Young | 120 | 2 | 6.57 | 6.8 | 0.612 |
|  | Middle |  | 22 | 7.13 | 6.75 | 0.36 |
|  | Old |  | 4 | 7.4 | 6.83 | 0.233 |
|  | Young | 150 | 2 | 5.85 | 9.3 | 0.517 |
|  | Middle |  | 8 | 6.67 | 6.39 | 0.556 |
|  | Old |  | 2 | 9.09 | 9.44 | 0.481 |
|  | Young | 180 | 2 | 6.57 | 8.58 | 0.553 |
|  | Middle |  | 6 | 6.7 | 5.87 | 0.12 |
|  | Old |  | 2 | 9.09 | 7.7 | 0.181 |
| PEF^c^  (L/min) | Young | 30 | 1 | 410 | 517.8 | NA^d^ |
|  | Middle |  | 46 | 326.7 | 328.26 | 0.907 |
|  | Old |  | 8 | 241.2 | 251.84 | 0.314 |
|  | Young | 60 | 0 | NA | NA | NA |
|  | Middle |  | 41 | 329.25 | 334.89 | 0.697 |
|  | Old |  | 6 | 260.85 | 242.74 | 0.544 |
|  | Young | 90 | 0 | NA | NA | NA |
|  | Middle |  | 38 | 338.65 | 324.06 | 0.448 |
|  | Old |  | 6 | 244.9 | 226.05 | 0.563 |
|  | Young | 120 | 1 | 410 | 488.5 | NA |
|  | Middle |  | 38 | 334.53 | 338.6 | 0.848 |
|  | Old |  | 6 | 262.52 | 232.29 | 0.458 |
|  | Young | 150 | 0 | NA | NA | NA |
|  | Middle |  | 34 | 326.23 | 323.78 | 0.917 |
|  | Old |  | 6 | 262.52 | 228.93 | 0.419 |
|  | Young | 180 | 1 | 410 | 421 | NA |
|  | Middle |  | 34 | 321.23 | 319 | 0.923 |
|  | Old |  | 6 | 262.52 | 248.44 | 0.733 |

^a^SBP: systolic blood pressure.

^b^FBG: fast blood glucose.

^c^PEF: peak expiratory flow.

^d^NA: not applicable.

**Table 3**. Comparison of patient outcomes over different time spans for male and female patients.

| Patient outcome | Gender | Period  (days) | Number of patients  who have records | Mean value  (before) | Mean value  (after) | *P* value |
| --- | --- | --- | --- | --- | --- | --- |
| SBP^a^  (mmHg) | Male | 30 | 721 | 132.26 | 128.54 | **<0.001** |
|  | Female |  | 419 | 131.68 | 126.96 | **<0.001** |
|  | Male | 60 | 736 | 128.76 | 126.14 | **<0.001** |
|  | Female |  | 527 | 128.73 | 125.46 | **<0.001** |
|  | Male | 90 | 603 | 130.26 | 127.64 | **<0.001** |
|  | Female |  | 405 | 130.78 | 128.91 | **<0.001** |
|  | Male | 120 | 424 | 132.09 | 128.32 | **<0.001** |
|  | Female |  | 301 | 133.09 | 129.64 | **<0.001** |
|  | Male | 150 | 298 | 130.65 | 127.13 | **<0.001** |
|  | Female |  | 148 | 131.12 | 128.54 | **0.005** |
|  | Male | 180 | 253 | 130.38 | 127.63 | **<0.001** |
|  | Female |  | 150 | 131.88 | 129.1 | **0.028** |
| FBG^b^  (mmol/L) | Male | 30 | 51 | 6.76 | 6.51 | 0.246 |
|  | Female |  | 25 | 7.08 | 7.16 | 0.844 |
|  | Male | 60 | 244 | 5.2 | 4.95 | **<0.001** |
|  | Female |  | 213 | 5.01 | 4.84 | **0.05** |
|  | Male | 90 | 31 | 6.78 | 6.65 | 0.592 |
|  | Female |  | 27 | 6.66 | 6.49 | 0.381 |
|  | Male | 120 | 17 | 7.3 | 6.86 | 0.401 |
|  | Female |  | 11 | 6.86 | 6.62 | 0.361 |
|  | Male | 150 | 5 | 6.84 | 6.55 | 0.693 |
|  | Female |  | 7 | 7 | 7.98 | 0.387 |
|  | Male | 180 | 6 | 6.74 | 6.82 | 0.939 |
|  | Female |  | 4 | 7.77 | 6.72 | 0.082 |
| PEF^c^  (L/min) | Male | 30 | 49 | 317.64 | 322.81 | 0.679 |
|  | Female |  | 6 | 300.82 | 302.46 | 0.943 |
|  | Male | 60 | 42 | 322.25 | 329.42 | 0.615 |
|  | Female |  | 5 | 305.95 | 270.27 | 0.249 |
|  | Male | 90 | 41 | 329.47 | 319.38 | 0.568 |
|  | Female |  | 3 | 275.25 | 192.05 | 0.247 |
|  | Male | 120 | 40 | 328.14 | 330.23 | 0.92 |
|  | Female |  | 5 | 314.35 | 308.03 | 0.819 |
|  | Male | 150 | 37 | 320.03 | 317.47 | 0.908 |
|  | Female |  | 3 | 275.25 | 211.88 | 0.252 |
|  | Male | 180 | 37 | 316.21 | 313.41 | 0.898 |
|  | Female |  | 4 | 301.86 | 290.32 | 0.759 |

^a^SBP: systolic blood pressure.

^b^FBG: fast blood glucose.

^c^PEF: peak expiratory flow.
