# Supplementary material for: Pathway-Driven Coordinated Telehealth System for Management of Patients With Single or Multiple Chronic Diseases in China: System Development and Retrospective Study
Source: JMIR Med Inform. 2021 May 17;9(5):e27228. doi: 10.2196/27228 (PMC8167615; doi:10.2196/27228)
Supplement: Multimedia Appendix 6 [file medinform_v9i5e27228_app6.docx]

**Detailed results of refutation in causal inference**

In this supplementary material, we provide the detailed results of refutation for the proposed causal model used in the main text. The refutation will be conducted on different physiological indices (SBP, FBG, and PEF) and different causal inference methods (PSS and PSM) separately. Concretely, we conducted three types of refutation:

1. Adding a random common cause variable (RCCV). The expected result is that the estimate value doesn’t change after we add an independent random variable as a common cause to the dataset.
2. Adding an unobserved common cause variable (UCCV). The expected result is that the estimate value doesn’t show too much sensitivity when we add an additional common cause to the dataset that is correlated with the treatment and the outcome.
3. Replacing treatment with a random (placebo) variable (TRV). The expected result is that the estimate value should go to zero when we replace the true treatment variable with an independent random variable. A *P* value will be provided to compare the mean value of new estimated effect with zero (P > 0.05 demonstrates that there is no significant difference between the mean new effect value and zero.

The results of each index on different causal inference methods are presented in the following tables. According to the refutation results, most of the estimands return an expectable value, except for the RCCV and UCCV of PEF value. A possible explanation for this is that the distribution of outcome and selected confounders among COPD patients had a large discrepancy, which led to different intermediate results when performing stratification and matching. Moreover, certain bias might exist in the extraction strategy itself for causal data. Overall, we believe that the causality results provided in the main text have an acceptable level of credibility.

Table 1. Refutation results for SBP.

| Refutation strategy | Causal inference methods | Estimated Effect | New Effect | *P* Value (for TRV) |
| --- | --- | --- | --- | --- |
| RCCV | PSM | -5.24 | -5.53 | NA |
|  | PSS | -5.51 | -5.53 | NA |
| UCCV | PSM | -5.24 | -4.79 | NA |
|  | PSS | -5.51 | -5.40 | NA |
| TRV | PSM | -5.24 | 0.08 | 0.42 |
|  | PSS | -5.51 | 0.01 | 0.43 |

Table 2. Refutation results for FBG.

| Refutation strategy | Causal inference methods | Estimated Effect | New Effect | *P* Value (for TRV) |
| --- | --- | --- | --- | --- |
| RCCV | PSM | -1.82 | -1.73 | NA |
|  | PSS | -1.27 | -1.28 | NA |
| UCCV | PSM | -1.82 | -1.83 | NA |
|  | PSS | -1.27 | -1.21 | NA |
| TRV | PSM | -1.82 | 0.03 | 0.33 |
|  | PSS | -1.27 | 0.02 | 0.47 |

Table 3. Refutation results for PEF.

| Refutation strategy | Causal inference methods | Estimated Effect | New Effect | *P* Value (for TRV) |
| --- | --- | --- | --- | --- |
| RCCV | PSM | 10.08 | 5.56 | NA |
|  | PSS | 2.36 | 9.33 | NA |
| UCCV | PSM | 10.08 | 15.94 | NA |
|  | PSS | 2.36 | 30.55 | NA |
| TRV | PSM | 10.08 | 1.42 | 0.44 |
|  | PSS | 2.36 | -0.04 | 0.43 |
